# Folding and Duplex Formation in Sequence-Defined Aniline Benzaldehyde Oligoarylacetylenes

**DOI:** 10.1021/jacs.2c06268

**Published:** 2022-09-29

**Authors:** Kyle R. Strom, Jack W. Szostak

**Affiliations:** Howard Hughes Medical Institute, Department of Molecular Biology, and Center for Computational and Integrative Biology, Massachusetts General Hospital, Boston, Massachusetts 02114, United States

## Abstract

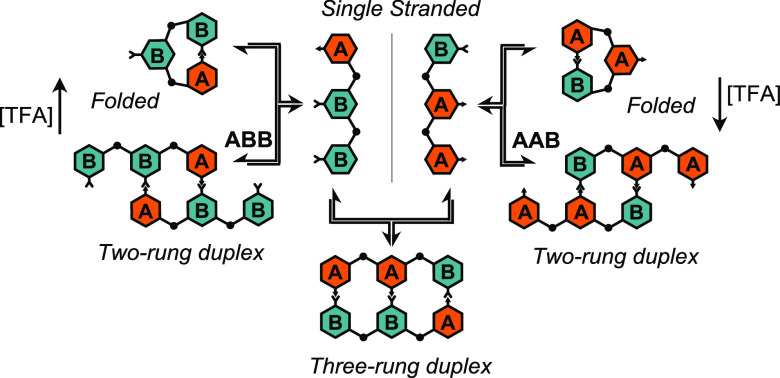

In all known genetic polymers, molecular recognition via hydrogen bonding between complementary subunits underpins their ability to encode and transmit information, to form sequence-defined duplexes, and to fold into catalytically active forms. Reversible covalent interactions between complementary subunits provide a different way to encode information, and potentially function, in sequence-defined oligomers. Here, we examine six oligoarylacetylene trimers composed of aniline and benzaldehyde subunits. Four of these trimers self-pair to form two-rung duplex structures, and two form macrocyclic 1,3-folded structures. The equilibrium proportions of these structures can be driven to favor each of the observed structures almost entirely depending upon the concentration of trimers and an acid catalyst. Quenching the acidic trimer solutions with an organic base kinetically traps all species such that they can be isolated and characterized. Mixtures of complementary trimers form exclusively sequence-specific 3-rung duplexes. Our results suggest that reversible covalent bonds could in principle guide the formation of more complex folded conformations of longer oligomers.

## Introduction

Nucleic acid polymers play a central role in the biochemistry of all known life. Their structures underlie the mechanism by which evolution sculpts macromolecular catalysts within a vast sequence space. Arguably, the essential feature of these polymers is the molecular recognition between complementary nucleobase subunits that enables base-pairing. It is base-pairing that allows for the transfer of information through templated polymerization and the folding of nucleic acid polymers into catalytically active structures.

The RNA-world hypothesis suggests that RNA innately possesses the means to initiate the transition from chemistry to biology without the complex biomachinery of extant biology.^1^ In support of this, both information transfer^2^ and the assembly of catalytically active ribozymes have been demonstrated in nonenzymatic RNA systems.^3^ However, many alternative RNA-like polymers, collectively referred to as XNAs, are also capable of both information transfer and function.^4^ Thus, at least some structural variation can be tolerated while maintaining these core properties. We are interested in whether synthetic sequence-defined polymers with fundamentally different molecular designs might also exhibit the properties of information transfer and function.

To fully mirror the evolutionary behavior of nucleotide polymers, synthetic polymers must be able to store and propagate information, and either directly through folding (e.g., ribozymes) or indirectly through translation (e.g., proteins), the information they store must encode structures with varying fitness. For a synthetic polymer, this requires overcoming challenges in building monodisperse sequence-defined polymers that self-assemble into chemically active configurations (e.g., foldamers) and that can undergo template-directed polymerization. Compounding these challenges, the presence of the complementary recognition subunits required for templated polymerization means that genetic polymers will contain mutually reactive subunits with the potential to self-associate and fold in a multitude of ways. Despite these challenges, significant strides have been made in recent decades in the synthesis of polymers that mimic biomolecules:^5^ information storing sequence-defined polymers can now be made to considerable lengths,^6^ polymers that mimic the folding behavior of enzymes and ribozymes are built with ever-increasing sophistication,^7^ and many synthetic sequence-dependent duplex forming structures have been reported.^8^

Reversible covalent bonds have been remarkably useful in producing a range of life-like complex behaviors in synthetic systems. Two notable examples are the self-replication of disulfide-bound macrocycles reported by Otto and co-workers^9^ and the dynamic templated self-assembly of helices by Nitschke and co-workers.^10^ In contrast to the chemistry developed herein, these systems do not utilize molecules that store sequence information. As such, they do not possess an obvious mechanism for the transfer of information like the nonenzymatic-templated polymerization observed for nucleobase polymers.

Recently, we reported that reversible covalent imine bonds can template sequence information transfer in macrocyclic dimers.^11^ To our knowledge, this is one of the two examples of sequence information transfer using reversible covalent bonds.^12^ To expand our system to longer oligomers, we then developed a solid-phase synthesis of sequence-defined aniline benzaldehyde oligoarylacetylenes (ABOs).^13^ During that work, it became apparent that controlling the interactions of the reactive aniline **A** and benzaldehyde **B** subunits was essential to increasing the complexity of these systems.

**Scheme 1:**
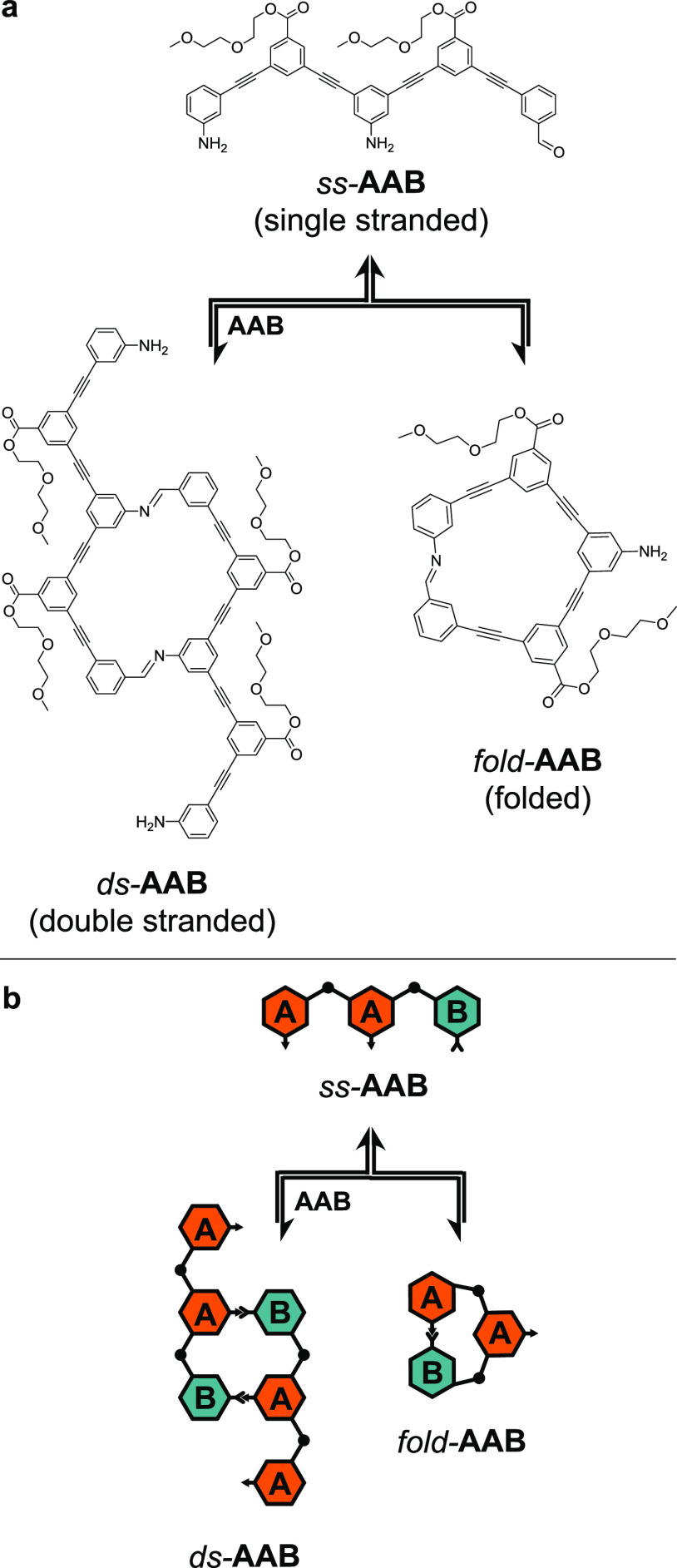
Dynamic Folding and Duplex Formation in an ABO Trimer with the Sequence **AAB**^a^ (a) Skeletal structures of *ss-*, *ds*-, and *fold*-**AAB**. (b) Cartoon structures of *ss*-, *ds*-, and *fold*-**AAB**.

To that end, we have been exploring sequence-defined ABO trimers (Scheme 1). ABOs are intentionally very different from genetic biopolymers. They are insoluble in water and use reversible covalent imine bonds as recognition subunits. Herein, we describe the dynamic behavior of a complete set of ABO trimers. We were pleased to find a range of behaviors even in this small sequence space. Scheme 1 depicts one of the key transformations we observed: when treated with an acid catalyst, a single-stranded trimer with the sequence **AAB** (*ss*-**AAB**) formed a two-rung duplex with unpaired sticky ends (*ds*-**AAB**) and folded into a macrocycle with an unpaired internal monomer (*fold*-**AAB**).

Self-association and folding behavior have been explored extensively in synthetic sequence-defined oligomers with noncovalent H-bonding recognition subunits,^8d,8e,8j^ but, to the best of our knowledge, these interactions have not been described in reversible covalent chemistry systems with sequence-defined oligomers. Aniline benzaldehyde oligomers with a peptoid backbone have been shown to self-sort into sequence-encoded polyimine duplexes in peptoid pentamers by the Scott laboratory.^8i^ While self-association and folding behavior were not the focus of their report, the authors mention that they suspect some amount of folding likely occurred under the conditions they studied.

As imine bonds are only conditionally reversible, they offer some advantages over H-bonding systems; their covalent base-paring interactions do not rely on association constants but can be driven to completion by the removal of water; furthermore, aromatic imine bonds do not typically equilibrate if they are protected from catalysts, water, and harsh conditions.^14^

Notably, these molecules differ from biopolymers and many synthetic informational polymers in that they lack a directional polarity. This greatly simplifies their dynamic behavior as duplexes do not have parallel or antiparallel geometries. Likewise, the lack of directionality restricts the size of the sequence space due to symmetry considerations (e.g., **AAB** is the same as **BAA**).

## Results and Discussion

### Synthesis of Trimers

**Figure 1:**
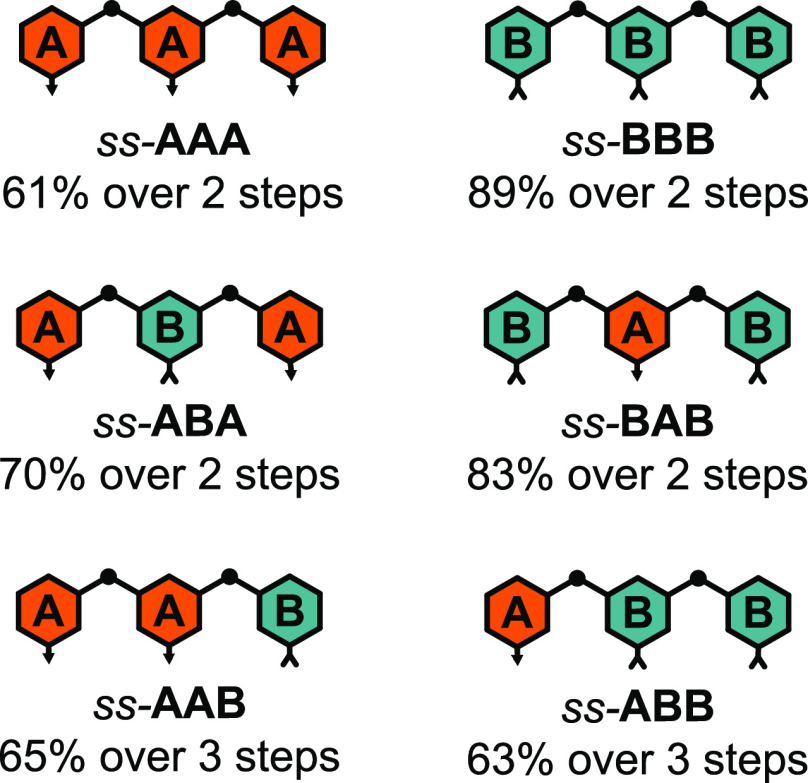
*ss*-ABO trimers. Isolated yields over the number of steps indicated.

The entire sequence space of ABO trimers consists of the six sequences depicted in Figure 1. Single-stranded trimers were synthesized via Sonogashira coupling^13,15^ in high yields in two or three steps as noted in Figure 1 and described in detail in the Supporting Information. If the trimers were protected from acid, no premature imine bond formation between **A** and **B** subunits was observed, save a few percent when solutions were concentrated to dryness.

As they are homopolymers, **AAA** and **BBB** do not contain mutually reactive subunits, so no self-association behavior from imine bond formation was expected or observed. Conversely, as the remaining trimers do contain mutually reactive **A** and **B** subunits, we expected that these molecules would assemble into higher order structures under conditions favoring imine bond formation. In order to investigate this behavior, we conducted a series of NMR experiments in the presence of trifluoracetic acid (TFA) as an imine bond-forming acid catalyst. The results of these experiments are presented in the next section.

### Folding and Duplex Formation of ABO Trimers

**Scheme 2:**
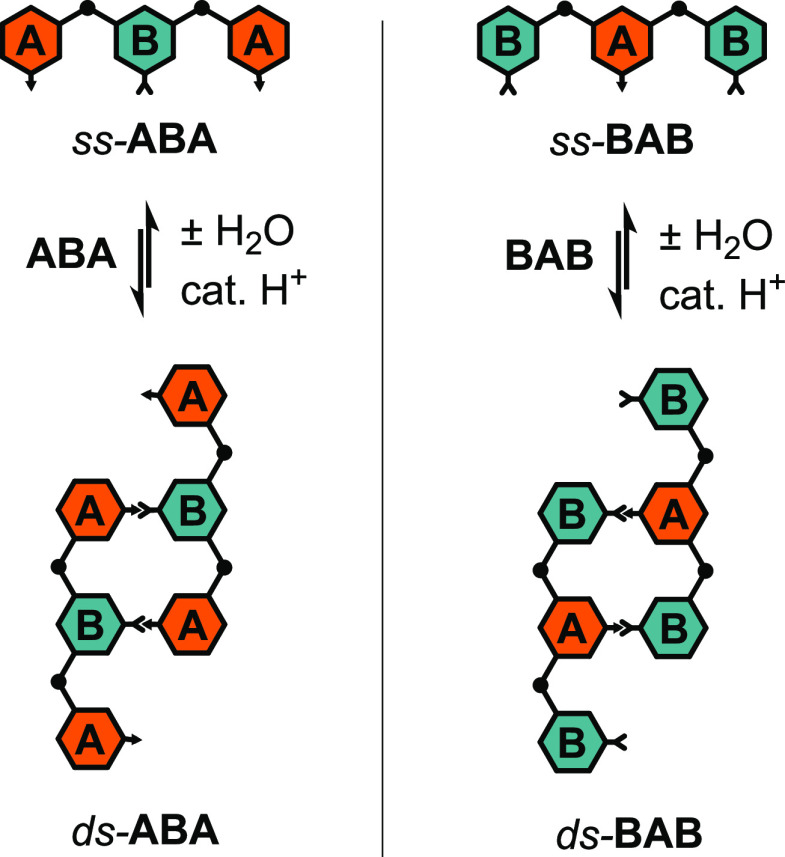
Reversible Acid-Catalyzed Condensation of *ss*-**ABA** and *ss*-**BAB** to *ds*-**ABA** and *ds*-**BAB**

For the isolated ABO trimers **ABA** and **BAB**, we found that formation of the partially double-stranded duplex structures *ds*-**ABA** and *ds*-**BAB** (Scheme 2) was straightforward and could be accomplished nearly quantitatively under optimized conditions. As we observed no major differences in the behaviors of **ABA** and **BAB**, we will limit our discussion in this section to the **ABA** trimer and refer the reader to the Supporting Information for the analogous data on the **BAB** trimer (Figures S1 and S2).

**Figure 2:**
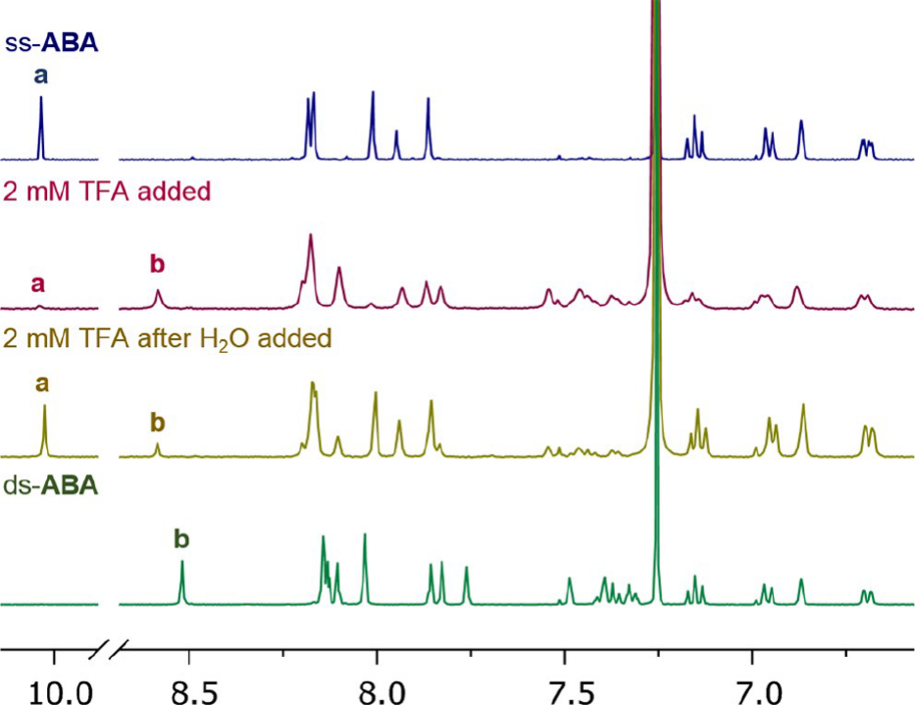
Portion of the ^1^H NMR spectra of **ABA** (3.5 mM in CDCl_3_) under different conditions as labeled. Relevant ^1^H resonances labeled: (a) aldehyde *ss*-**ABA** and (b) imine *ds*-**ABA**.

The aromatic region of the ^1^H NMR spectrum of ss-**ABA** (3.5 mM in CDCl_3_) is shown in Figure 2. Immediately, after the addition of a small amount of TFA, the downfield aldehyde resonance greatly decreased in intensity, and new peaks consistent with the imine protons and aromatic protons of *ds*-**ABA** appeared (Figure 2, second spectrum). Only a small amount of *ss*-**ABA** remained, as evidenced by the small aldehyde peak at δ 10.04 ppm. NMR spectra taken after allowing the solution to sit for several hours showed no change from the initial spectrum, suggesting that the reaction had reached equilibrium within the few minutes required to acquire the initial ^1^H NMR spectrum. The integrals of the aldehyde peak relative to that of the imine peak show that under these conditions (2 mM TFA), *ds*-**ABA** is favored over *ss*-**ABA** by 10 > 1. Reactions run at higher concentrations of **ABA** (7.5 and 15 mM) gave a similar ratio of *ds-* to *ss*-**ABA**. At 15 mM, we observed a small amount of an uncharacterized species (Figure S3) which may represent a higher order multiplex structure.

Notably, in the presence of TFA, the NMR spectra showed a significant peak broadening, an upfield shift for the imine resonance, and a downfield shift for the aromatic resonances. This was greatly exacerbated by increasing the concentration of TFA (Figure S4). We suspected that protonation of the basic imine and aniline sites on *ds*-**ABA** promotes the aggregation of these molecules and is responsible for the peak broadening and chemical shift changes.^16^ Accordingly, an NMR spectrum of *ds*-**ABA** with sharp peaks was recovered by quenching the TFA with a 3-fold excess of triethylamine (TEA). Basified solutions were kinetically stable and could be washed with water, concentrated to dryness, or purified on silica gel without any apparent imine hydrolysis or the formation of higher order multiplexes. Conversely, if solutions of *ds*-**ABA** in the presence of TFA were not quenched with TEA, adding water led to immediate hydrolysis to *ss*-**ABA** (Figure 2, third spectrum).

We reasoned that the *ds-* to *ss-*equilibrium could be driven even further to *ds*-**ABA** by removing adventitious water in the solvent as well as water liberated during imine formation. A two-step procedure wherein *ss*-**ABA** (3.5 mM) in dry CDCl_3_ was first treated with TFA (2 mM), concentrated to dryness to remove water, and then redissolved in dry CDCl_3_ to the same concentration (3.5 mM) gave complete conversion to *ds*-**ABA**. Upon quenching with TEA, this procedure gave pure kinetically stable *ds*-**ABA**, which could be isolated and characterized (Figure 2, bottom).

**Figure 3:**
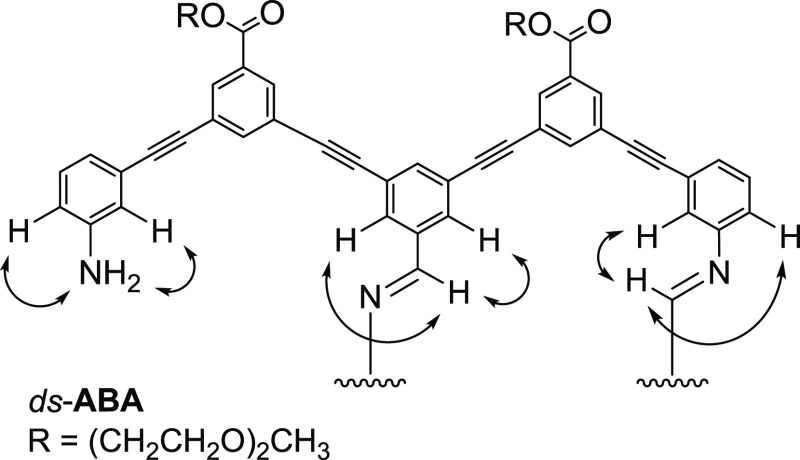
Key ^1^H–^1^H ROSEY correlations observed for *ds*-**ABA**. As *ds*-**ABA** is C2 symmetric, the other strand is magnetically equivalent to the half that is shown.

For *ds*-**ABA**, ^1^H–^1^H 2D-ROSEY NMR correlations are seen between the imine resonance and a downfield shifted spin system with no strong *J*-coupling (consistent with **B**) and a spin system with a singlet, doublet, doublet, triplet pattern (consistent with **A**). In the spectrum, there is another upfield shifted spin system consistent with **A**, which does not have ROSEY correlations to the imine protons but instead correlates to an upfield resonance consistent with N*H*_2_ (Figure 3). Taken together, this strongly supports the structure of *ds*-**ABA** which has the internal **B** paired through an imine bond to an **A** and one unpaired **A**. Structures annotated with NMR peak assignments and ROSEY and COSY correlations and HRMS data are provided in the Supporting Information (Figures S11 and S18).

**Scheme 3:**
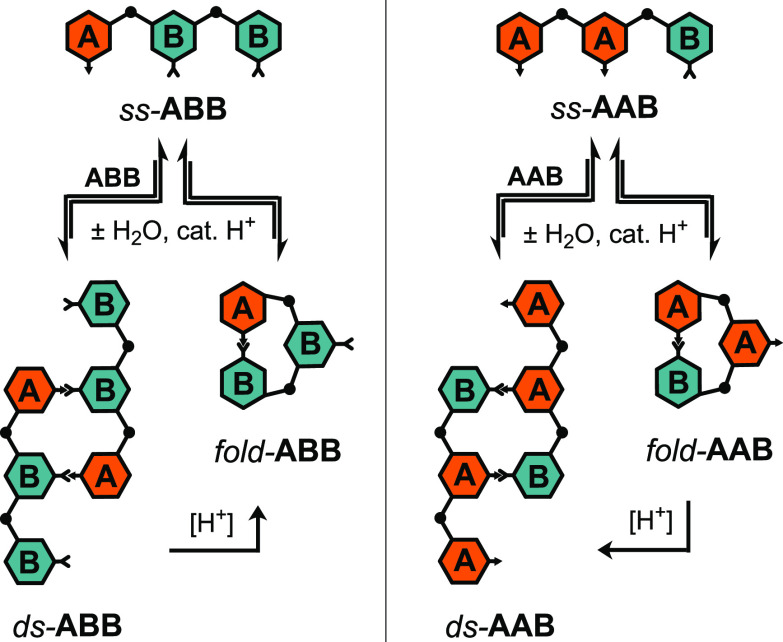
Reversible Acid-Catalyzed Condensation of *ss*-**ABB** and *ss*-**AAB** to *ds*-**ABB** and *ds*-**AAB** or *fold*-**ABB** and *fold*-**AAB**^a^ Increasing [TFA] was found to favor *fold*-**ABB** and *ds*-**AAB**.

The remaining two trimers in the ABO sequence space, **AAB** and **ABB**, proved the most complicated and interesting. Like **BAB** and **ABA**, these trimers can self-associate to form two-rung duplexes with unpaired sicky ends. In addition, since the terminal subunits of the single-stranded trimers are complementary, they can fold into macrocyclic structures with the internal monomer unpaired (Scheme 3).

While these folded structures were initially a surprise, simple MM2 energy minimizations show that the steric stain incurred by this 1,3-folding is relatively low (+5.68 kcal/mol **AAB**) and nearly identical to the strain incurred in forming the double-stranded structures (+5.69 kcal/mol **AAB**). In contrast, minimization of the 1,2-folded **AAB** structure shows significant strain on folding (+52.7 kcal/mol **AAB**), and 1,2-folding was not observed for any sequence in this study.

Hunter and co-workers have observed noncovalent H-bonded 1,2- and 1,3-folding in dimers^8d^ and trimers^8e^ with more flexible backbones than the *m*-phenylene ethynylene backbone used here. Recently, they described an *m*-phenylene ethynylene system which shows folding behavior between H-bonded subunits;^8j^ however, the sequence space they investigated did not contain any 1,3-sequence complementarity, so a direct comparison of these oligomers to those described here is not yet possible.

**Figure 4:**
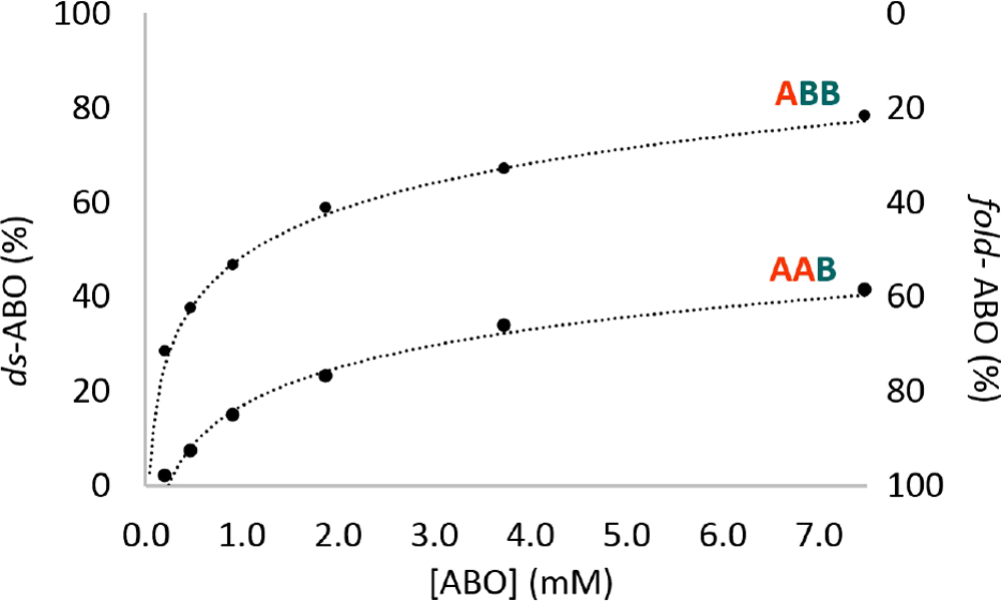
Concentration dependence of *fold*- vs *ds*-ABO in CDCl_3_ with 2 mM TFA.

We expected that the equilibrium ratio of *fold*- to *ds*-ABO would depend on the concentration of the trimers as folding is an intramolecular reaction, while duplex formation is intermolecular. We conducted a series of NMR experiments at different concentrations of trimers for both **AAB** and **ABB** with a constant concentration of TFA (2 mM). The ratio of folded to double-stranded structures was determined from the relative integral of the imine protons of each species. As expected, lower trimer concentrations favor the folded structures and higher concentrations favor the double-stranded structures (Figure 4). At the same concentration, **AAB** is more prone to folding than **ABB**. We subsequently found that these equilibria are sensitive to the concentration of TFA, with different sequences behaving differently (vide infra). The different equilibria may reflect this difference in acid sensitivity.

As was the case with the **ABA** and **BAB** trimers, quenching solutions of **ABB** and **AAB** with an organic base gave kinetically stable solutions, such that the *ds-* and *fold-*trimers could be isolated without re-equilibrating. Using Figure 4 as a guide, larger-scale reactions were run at the appropriate concentrations to favor the desired structure, and after quenching with TEA, these solutions were concentrated and purified on silica gel. The pure materials could be characterized by NMR at high concentrations without difficulty if a small amount of base (0.1% TEA) was employed to scavenge any adventitious acid. HRMS (Figure S18) and 2D NMR experiments (Figures S13–S16) provided strong evidence for the structural assignments of all four structures *ds*-**AAB**, *fold*-**AAB**, *ds*-**ABB**, and *fold*-**ABB**. As described above for *ds*-**ABA**, the analysis uses ^1^H–^1^H ROSEY NMR to identify monomers that are paired through imine bonds. The splitting patterns of those resonances and ^1^H–^1^H COSY NMR were then used to assign those monomers to internal or terminal positions. For all *ds*- and *fold*-ABOs, we observed strong and uniform ^1^H–^1^H ROSEY correlations between the imine proton and all four protons *ortho* to the imine bond. This suggests that rotation around the imine bond is relatively unhindered in these structures; if a single imine rotamer was strongly favored, only two of the four protons would be likely to be in a range to produce a strong ROE.

While investigating the concentration dependence described above, we observed a surprisingly dramatic shift in the downfield ^1^H NMR resonances of *fold-***ABB** as the concentration was lowered. We suspected that this might be the result of increasing acidity at lower trimer concentrations as there is less trimer to buffer the acidity.

**Figure 5:**
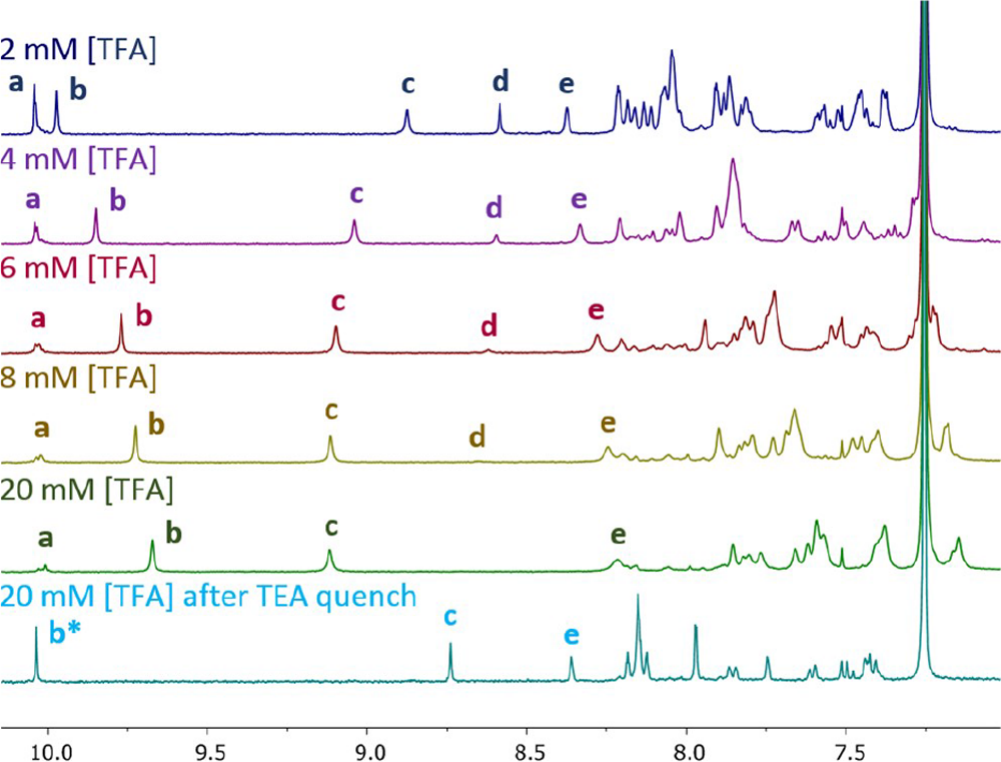
Portion of the ^1^H NMR spectra of **ABB** (550 uM in CDCl_3_) as TFA was titrated into the solution. Relevant ^1^H resonances labeled: (a) aldehyde *ds*-**ABB**, (b) aldehyde *fold*-**ABB**, (c) imine *fold*-**ABB**, (d) imine *ds*-**ABB**, and (e) aromatic proton *meta* to imine *fold*-**ABB**. *The aldehyde resonances of *fold*-**ABB** and *ds*-**ABB** overlap in basified solutions; this assignment is possible because only *fold*-**ABB** is present in this spectrum.

**Figure 6:**
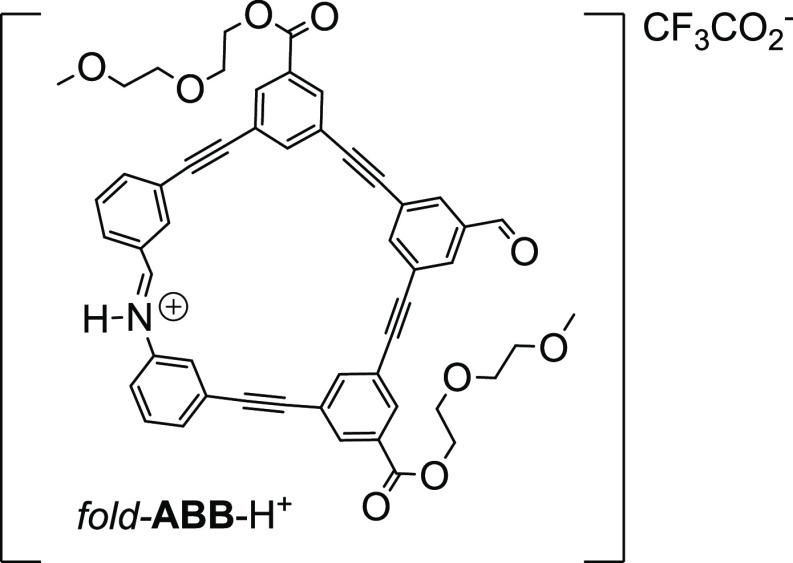
Structure of *fold*-**ABB**-H^+^.

To further investigate this, we ran a series of experiments where we observed the ^1^H NMR spectrum as TFA was titrated into solutions of **ABB**. With increasing acidity, we observed dramatic changes in the chemical shift of all resonances and a shift in the *ds* to *fold* equilibrium to entirely *fold-***ABB**. Figure 5 shows the ^1^H NMR spectra of a solution of **ABB** (0.55 mM in CDCl_3_) as TFA was added to the solution ([TFA] 2 to 20 mM)). The large downfield shift in the imine resonance of *fold-***ABB** (Figure 5c) is expected as this is the most basic site in the molecule and protonation at this site to give **ABB**-H^+^ would likely cause the observed shift (Figure 6). The shifts seen for the other resonances are consistent with the increasing acidity aiding the formation of *fold*-**ABB**-H^+^ aggregates, with π-stacking within the aggregates driving the upfield shift of the resonances.^17^ If this aggregation increases the stability of *fold*-**ABB**-H^+^ compared to that of *ds*-**ABB**, it would explain the shift in the equilibrium as well.

Quenching acidic solutions of **ABB** with a 3-fold excess of TEA gave solutions containing only *fold-***ABB** and reversed the peak broadening and chemical shift changes caused by the acid (Figure 5, bottom). Increasing the concentration of acid drove the equilibrium to nearly entirely *fold-***ABB** even for much more concentrated solutions which strongly favored *ds*-**ABB** at lower TFA concentrations. Both 4.4 and 17.5 mM solutions of **ABB** could be completely converted to *fold*-**ABB** by treatment with excess TFA (40 and 80 mM, respectively), followed by quenching with a 3-fold excess TEA (Figures S5 and S6).

Because many macrocycles have been shown to tightly bind negatively charged ions,^18^ we investigated the potential role of the trifluoroacetate counterion in the formation and aggregation of *fold*-**ABB** and *ds*-**ABB**. Titration experiments with tetraethylammonium trifluoroacetate showed no change in the ^1^H NMR spectra of *fold-***ABB** (Figure S7) or *ds*-**ABB** (Figure S8). This strongly suggests that trifluoroacetate does not bind to the unprotonated molecules in chloroform. Similarly, a titration with tetraethylammonium trifluoroacetate preformed in the presence of TFA showed that additional trifluoroacetate merely acts as a general base, and the results mirror the TFA quenching observed with triethylamine (Figure S9). Notably, the p*K*_a_ of TFA in acetonitrile is reported to be 12.65,^19^ so the ability of trifluoroacetate to function as a competent base in organic solvent is not surprising. Taken together, these titration experiments suggest that protonation is the driving force for the observed behaviors and that trifluoroacetate is a passive bystander.

To see if the equilibrium of *ds-* to *fold-***AAB** is similarly sensitive to the TFA concentration, we repeated the titration experiment with **AAB**. Surprisingly, we found the opposite result as with **ABB**. At higher concentrations of TFA, *ds-***AAB** is favored almost completely over fold-**AAB** (Figure S10). In contrast to **ABB**, dramatic chemical shift changes were not observed in these spectra, and only a peak broadening and loss of the *ds-***ABB** signal were observed in the NMR spectrum as the TFA concentration was increased. Sharp peaks could be recovered by quenching with TEA. We suggest that acid-mediated aggregation is also the driving force for this change in equilibrium with the *ds-***AAB** aggregate being favored over *fold-***AAB**.

### Three-Rung Duplex Formation

**Figure 7:**
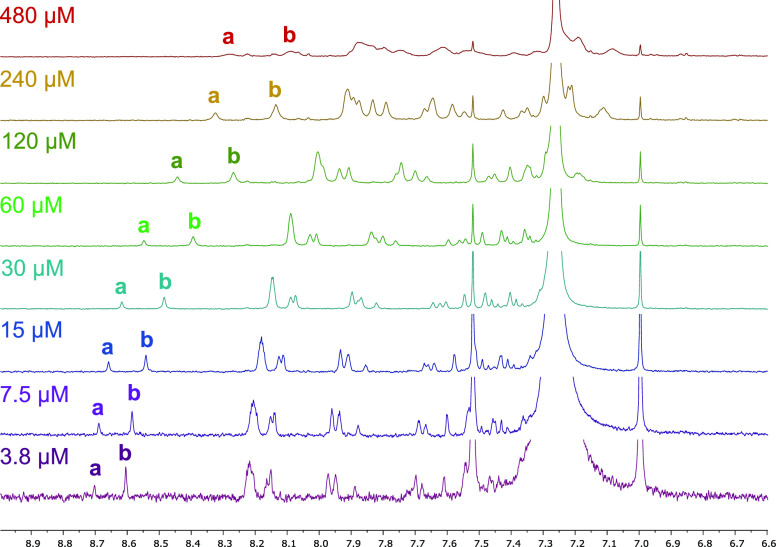
Portion of the ^1^H NMR spectra of *ds*-**AAA**·**BBB** in CDCl_3_ at rt over a dilution series from 480 μm to 3.8 μM. Relevant ^1^H resonances labeled: (a) 1H imine C*H*N from internal base pair and (b) 2H imine C*H*N from terminal base pairs.

**Scheme 4:**
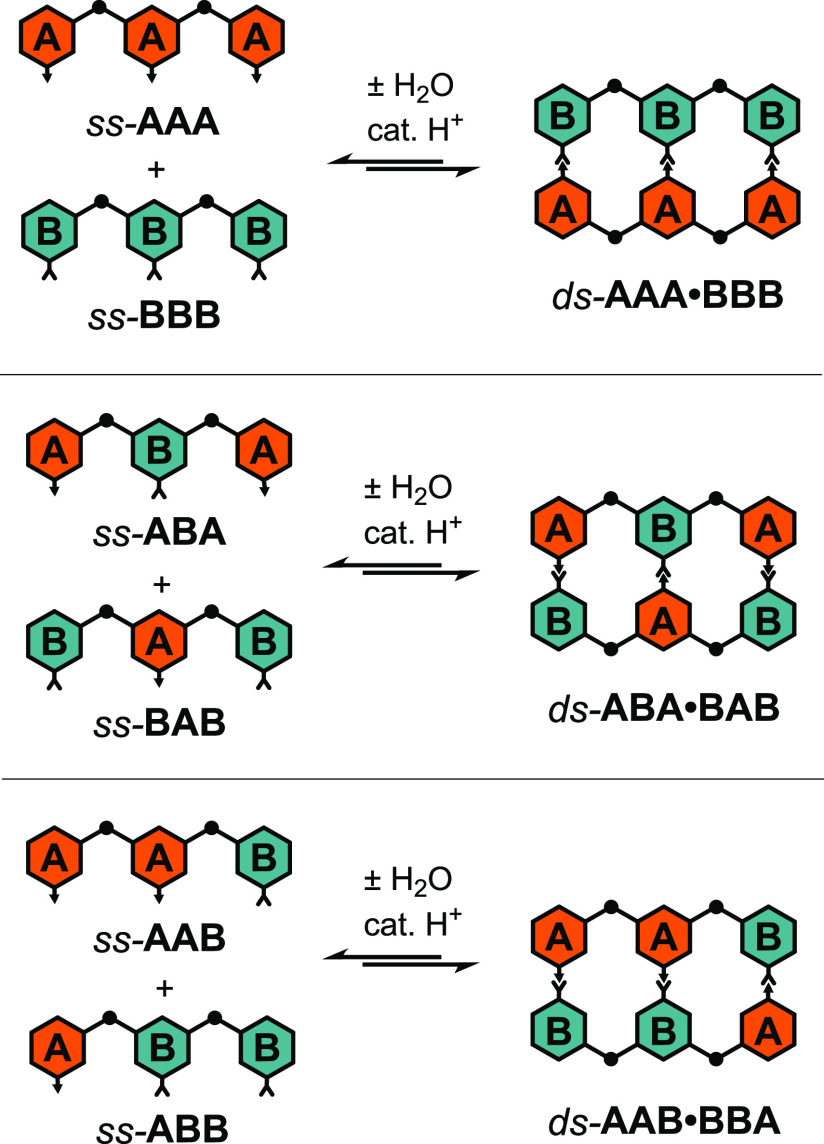
Acid-Catalyzed Condensation of Sequence Complementary Trimers to Form 3-Rung Duplexes

Given the ease with which partial 2-rung duplexes were formed via self-association, we asked whether mixtures of fully complementary strands would form 3-rung duplexes (Scheme 4). We initially assumed that the condensation of *ss*-**AAA** and *ss*-**BBB** to form *ds*-**AAA**·**BBB** would be the most straightforward as these sequences do not have competing self-association or folding behavior, and duplexes of similar polymers have been reported by Moore and co-workers.^20^ However, experiments where an equimolar mixture of *ss*-**AAA** (480 uM) and *ss*-**BBB** (480 uM) in CDCl_3_ was treated with TFA (1 mM) overnight gave NMR spectra with broad uninterpretable resonances, and quenching the TFA with an excess of TEA only had a small effect on sharpening the peaks (Figure 7, top spectrum). Notably, in the aforementioned work by the Moore laboratory, the polyimine duplexes needed to be reduced to amines prior to purification and characterization.

Suspecting that the peak broadening might be the result of aggregates formed through π-stacking interactions of the 3-rung duplexes, we acquired spectra over a dilution series at rt (Figure 7) and 50 °C (Figure S19). We found that both dilution and heat caused a downfield shift and a sharpening of the NMR resonances, consistent with concentration-dependent aggregation through π-stacking.^17a^ The NMR spectra indicate a nearly complete conversion to *ds*-**AAA**·**BBB**; no aldehyde peaks are present nor any upfield peaks from unpaired aniline subunits, and two imine peaks are present in a 1:2 ratio consistent with the three imine protons expected for *ds*-**AAA**·**BBB**. MALDI-TOF HRMS also supports the exclusive formation of the 3-rung duplexes (Figure S24).

For the remaining two mixtures of complementary sequences, *ss*-**ABA** with *ss*-**BAB** and *ss*-**AAB** with *ss*-**ABB** (Scheme 4, middle and bottom, respectively), the formation of 3-rung duplexes competes with the self-association and folding behavior described in the previous section (Schemes 2 and 3). Despite this added complexity, these mixtures behaved similarly to **AAA** and **BBB**, showing nearly complete conversion to *ds*-**ABA**·**BAB** and *ds*-**AAB**·**BBA** under acid-catalyzed imine-forming conditions and a concentration-dependent aggregation (Figures S20–S23). Remarkably, conversion to the 3-rung duplexes did not require the starting materials to be in their single-stranded states. A mixture of *ds*-**ABA**, *ss*-**ABA**, *ds*-**BAB**, and *ss*-**BAB** gave complete conversion to *ds*-**ABA**·**BAB** under the imine formation conditions, and a mixture of *ds*-**AAB**, *ss*-**AAB**, *fold*-**AAB**, *ds*-**ABB**, *ss*-**ABB**, and *fold*-**ABB** gave complete conversion to *ds*-**AAB**·**BBA**. This strongly suggests that imine exchange reactions occur quickly in the presence of TFA and the 3-rung products are thermodynamically favored to the exclusion of the 2-rung partial duplexes and folded structures. The ^1^H NMR spectrum for *ds*-**ABA**·**BAB** shows a small amount (approx. 10% by integration) of broad peaks of unknown species. It is possible these are the result of higher order polymers; however, the MADLI-TOF MS data only shows peaks consistent with the 3-rung duplexes.

**Figure 8:**
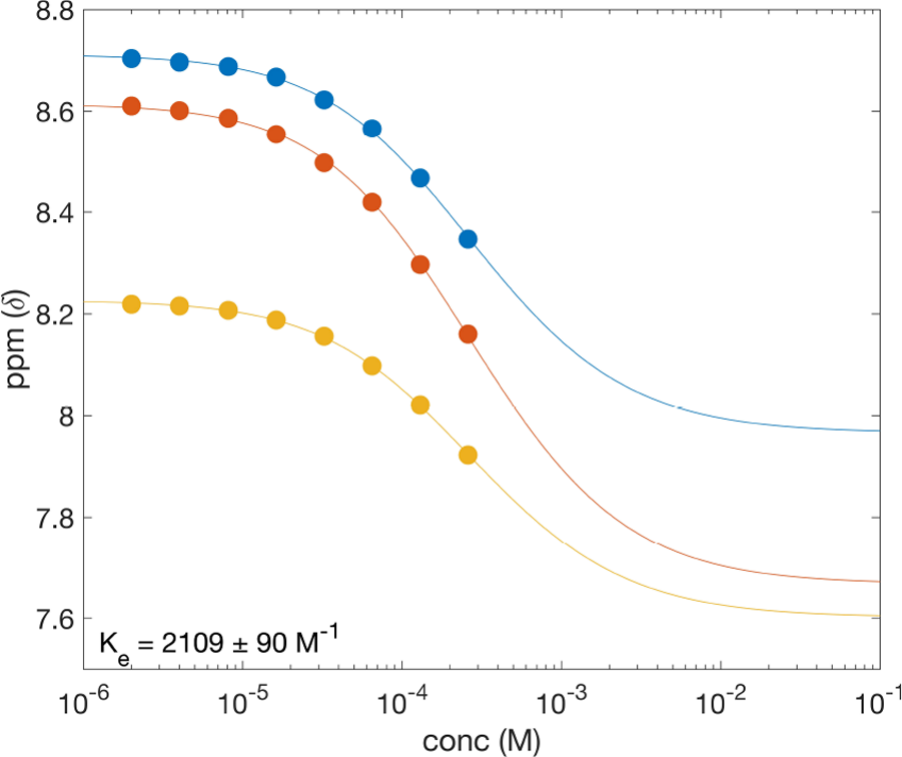
Concentration dependence of the ^1^H NMR chemical of shift of *ds*-**AAB**·**BBA** in CDCl_3_ at rt for three resonances: blue, the internal imine (N*H*C); red, the terminal imines (N*H*C); and yellow, the aromatic protons *meta* to the ester functional groups. Curves represent the line of best fit to the equal *K* model of indefinite association.

To quantitatively assess the 3-rung duplex aggregation behavior for all three species, we fit the chemical shift dependence of the duplex concentration to the equal *K* model of indefinite association.^21^Figure 8 shows the dependence of three ^1^H resonances of *ds*-**AAB**·**ABB** acquired in CDCl_3_ at rt. Curves for all three duplexes acquired at rt and 50 °C can be found in the Supporting Information (Figure S25). From the similarities in *K*_e_ (Table 1), we can conclude that sequence does not have a major effect on the tendency of these duplexes to aggregate, and mild heating significantly reduces their overall aggregation.

**Table 1:** Association Constants for 3-Rung ABO Duplexes

duplex	*K*_e_ (M^–1^) rt	*K*_e_ (M^–1^) 50 °C
*ds*-**AAA**·**BBB**	2971 ± 164	694 ± 72
*ds*-**ABA**·**BAB**	2295 ± 262	504 ± 49
*ds*-**AAB**·**BBA**	2109 ± 90	562 ± 54

Control of these macromolecular interactions will likely be essential to handling and studying longer ABO oligomers, and we expect conditions that minimize aggregation will be necessary for templated polymerization of this system. Attempts to assess the solvent dependency of the aggregation behavior were unsuccessful as the 3-rung duplexes were not sufficiently soluble in solvents such as acetonitrile, toluene, dimethyl-formamide, methanol, and benzene.

**Scheme 5:**
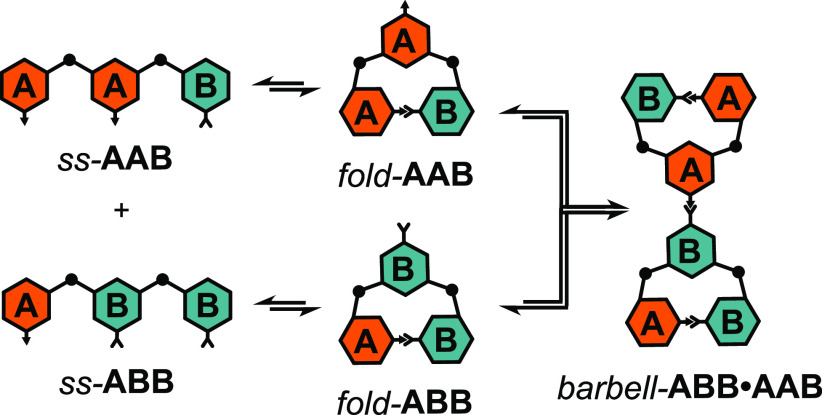
Formation of *barbell*-**AAB**·**BBA** via the Condensation of *fold*-**AAB** and *fold*-**ABB**

Given the propensity for **AAB** and **ABB** to fold under certain conditions, as discussed in the previous section, we were cautious in our assignment of *ds*-**AAB**·**BBA**. A hypothetical scenario where *fold*-**AAB** and *fold*-**ABB** condensed via a single imine bond would give *barbell*-**AAB**·**BBA** (Scheme 5), whose mass is identical to that of *ds*-**AAB**·**BBA**. Given the similarity of the *ds*-**AAB**·**BBA** NMR spectrum to *ds*-**ABA**·**BAB** and *ds*-**ABA**·**BAB**, we are confident in the exclusive formation of the 3-rung ladder structure in the presence of low [TFA] (1 mM). Interestingly, under higher [TFA] (20 mM), the NMR shows a minor product consistent with *barbell*-**AAB**·**BBB**; both *fold*-**AAB** and *fold*-**ABB** have a downfield shifted aromatic proton at 8.3 ppm which is not present in the ladder or partial ladder spectra. It is likely that these downfield shifted resonances would be present in *barbell*-**AAB**·**BBA** as we observe under high acid conditions (20 mM TFA). Unfortunately, our efforts to further characterize this minor product have been unsuccessful, potentially due to the ease of hydrolysis of the linking imine bond.

## Conclusions

Understanding and controlling the interactions of complementary recognition units within and between defined sequences are an essential step toward building synthetic polymers that will mimic the properties of genetic polymers. Our work shows that for ABO trimers, not only are both double-stranded and folded structures accessible, but their equilibrium can also be modulated by controlling their sequence and environment, and that mixtures of sequence complementary trimers form sequence paired duplexes.

Of the six higher order *ds* and *fold* structures that we observed in the limited ABO trimer sequence space, four could be generated nearly quantitatively without the need for purification, while the remaining two could be formed in good yield by controlling the concentration of TFA and ABO and then purified to give pure compounds (Table 2).

**Table 2:** Key Reaction Parameters to Favor Each of the *fold* and *ds* Structures Starting from *ss*-ABOs in CDCl_3_

compound	[ABO] mM	[TFA] mM	% yield
*ds-***ABA**	7.5	2	>95^a^
*ds-***BAB**	7.5	2	>95^a^
*ds-***AAB**	7.5	20	>95^a^
*ds-***ABB**	7.5	2	66^b^
*fold-***AAB**	0.1	2	79^b^
*fold-***ABB**	7.5	20	>95^a^

a Isolated yield following aqueous workup.

b Isolated yield following silica gel purification.

The acid dependence of imine equilibrium reactions is a useful feature of this system, allowing simple quenching with TEA to kinetically trap all species. No good analogy to this exists for short oligonucleotides or synthetic oligomers that use H-bonding recognition subunits; solution-phase equilibria are always likely to reassert themselves until oligomers are sufficiently long to become kinetically metastable. In contrast, for reversible covalent imine bonds, a single base pair is sufficient to form a kinetically stable and isolable structure (e.g., *fold*-**AAB** and *fold*-**ABB**). This behavior has important implications for longer oligomers. While covalent base pairs are likely to make longer oligomers more susceptible to inescapable kinetic traps,^22^ they may also confer the ability to exhibit useful and diverse folding behavior in much shorter systems.

For the **AAB** and **BBA** trimers, the formation of partial two-rung duplexes and 1,3-folded structures did not impede the formation of 3-rung duplexes in the presence of the fully complementary strand. This sequence-specific self-sorting matches the behavior of short polyimine peptoids;^8i^ however, it differs from nucleotide polymers of a similar length as a three-base-pair RNA duplex is highly unstable.

Ultimately, the utility of longer oligomers will depend on how easily their many competing interactions can be controlled. The methods described herein offer a necessary level of control and synthetic tractability in mixed sequence oligomers that use reversible covalent bonds as recognition subunits. Further elucidating the behaviors of oligomers in this family of molecules is likely to offer insights into the chemical space of genetic polymers beyond the nucleic acids and provide access to novel synthetic polymers that mimic the behaviors of biopolymers.

## Supporting Information Available

Experimental methods, NMR and HRMS characterization data and spectra, NMR spectroscopic data for TFA and tetraethylammonium trifluoroacetate tritrations, and chemical shift concentration dependence curves (PDF)
