# Supplementary material for: Folding and Duplex Formation in Sequence-Defined Aniline Benzaldehyde Oligoarylacetylenes
Source: J Am Chem Soc. 2022 Sep 29;144(40):18350–8. doi: 10.1021/jacs.2c06268 (PMC9562438; doi:10.1021/jacs.2c06268)

Folding and Duplex Formation in Sequence-Defined Aniline Benzaldehyde Oligoarylacetylenes

Kyle R. Strom and Jack W. Szostak*

*szostak@molbio.mgh.harvard.edu

SUPPORTING INFORMATION

Contents

I. [**BAB**] dependence of *ds*-**BAB** formation............................ S2

II. [TFA] dependence of *ds*-**BAB** formation............................ S3

III. [**ABA**] dependence of *ds*-**ABA** formation.......................... S4

IV. [TFA] dependence of *ds*-**ABA** formation........................... S5

V. [TFA] dependence of *fold*-**ABB** formation.......................... S6

VI. [TEA-TFA] dependence of **ABB** formation……………..…. S7

VII. [TFA] dependence of *fold*-**AAB** formation........................ S10

VIII. ^{1}H-^{1}H ROSEY and ^{1}H-^{1}H COSY NMR correlations.......... S12

IX. ESI-TOF HRMS data for ss-, ds-, and fold-ABOs……..…. S18

X. 3-rung duplex chemical shift concentration dependence…. S20

XI. 3-rung duplex MALDI-TOF MS spectra……………………. S25

XII. Experimental Section... S27

XIII. NMR Spectra.. S40

Figure S1. Relevant section of the ^{1}H NMR spectra showing the conversion of *ss*-**BAB** to *ds*-**BAB** at different [**BAB**] after treatment with TFA (2 mM) and quenching with TEA (6 mM). The C*HN* imine peaks for *ds*-**BAB**, the C*H*O aldehyde peaks for *ds*-**BAB** and *ss*-**BAB**, and the aromatic peaks from the **A** subunit of *ss*-**BAB** are labeled as such.

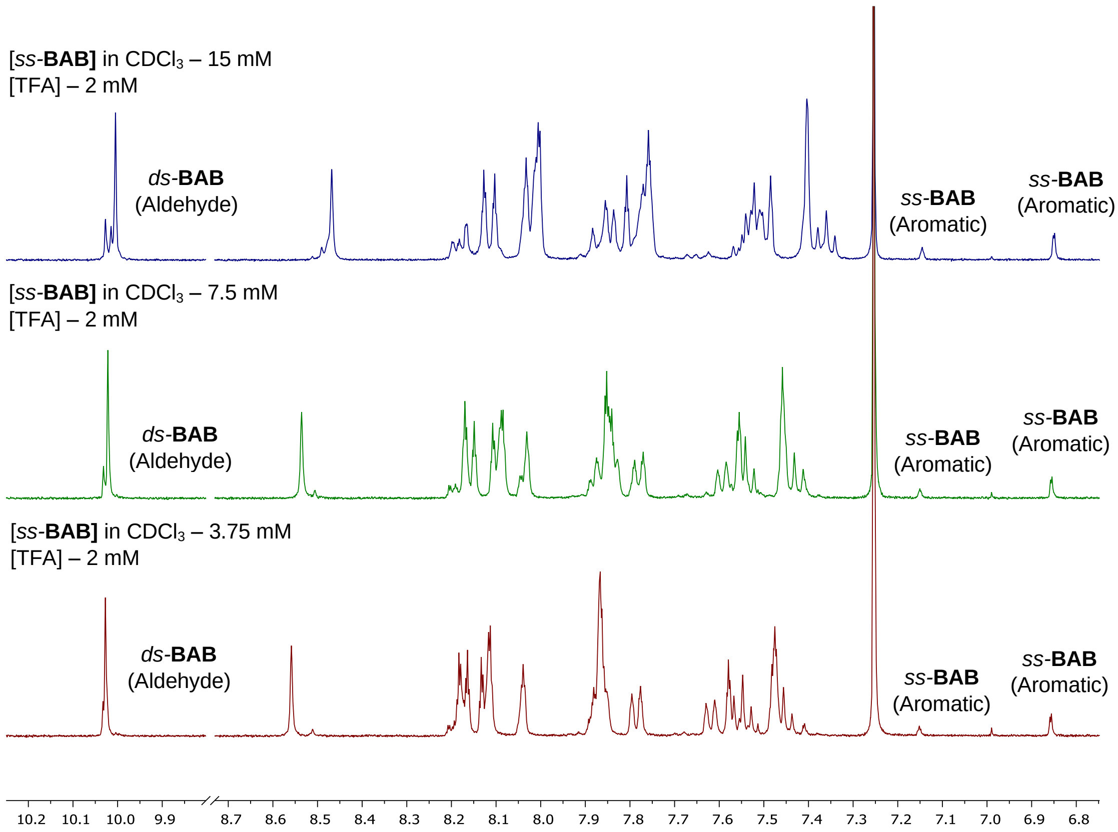

Figure S2. Relevant section of the ^{1}H NMR spectra showing the conversion of *ss*-**BAB** to *ds*-**BAB** after treatment with different amounts of TFA. The C*HN* imine peaks for *ds*-**BAB**, the C*H*O aldehyde peaks for *ds*-**BAB** and *ss*-**BAB**, and the aromatic peaks from the **A** subunit of *ss*-**BAB** are labeled as such.

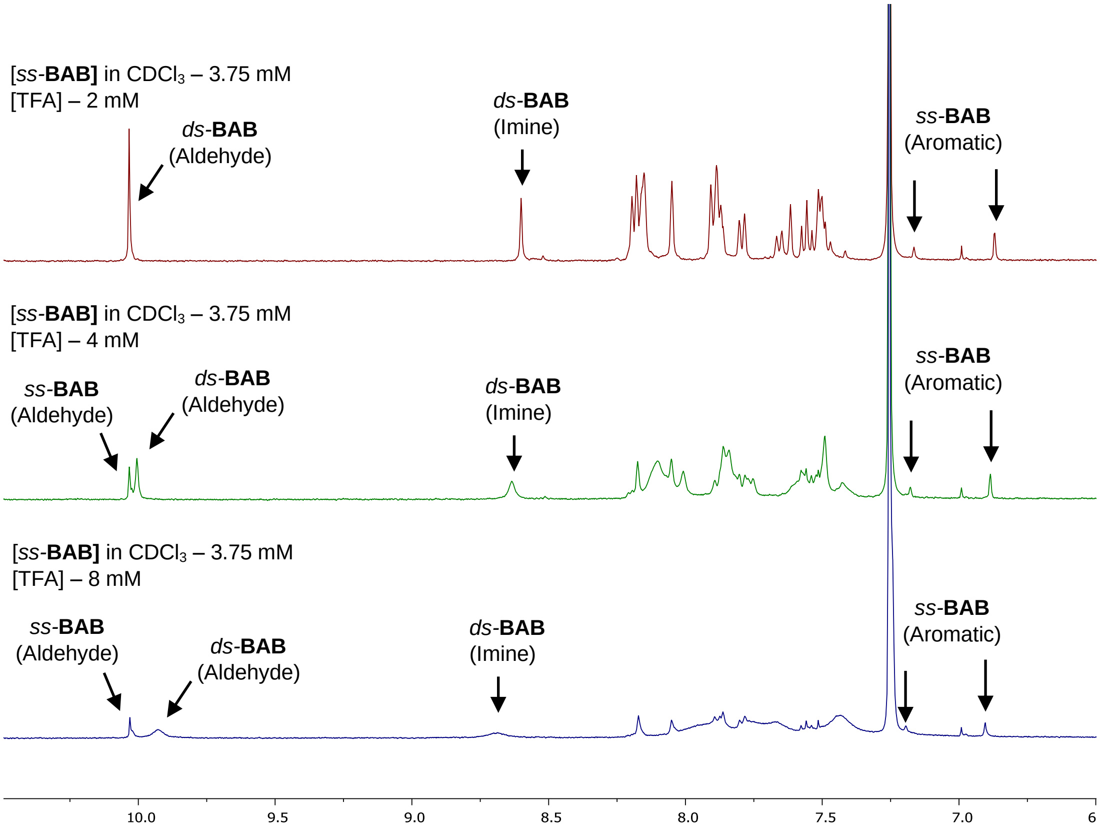

Figure S3. Relevant section of the ^{1}H NMR spectra showing the conversion of *ss*-**ABA** to *ds*-**ABA** at different [**ABA**] after treatment with TFA (2 mM). The C*H*N imine peaks for *ds*-**ABA** and the C*H*O aldehyde peaks for *ss*-**ABA** are labeled as such.

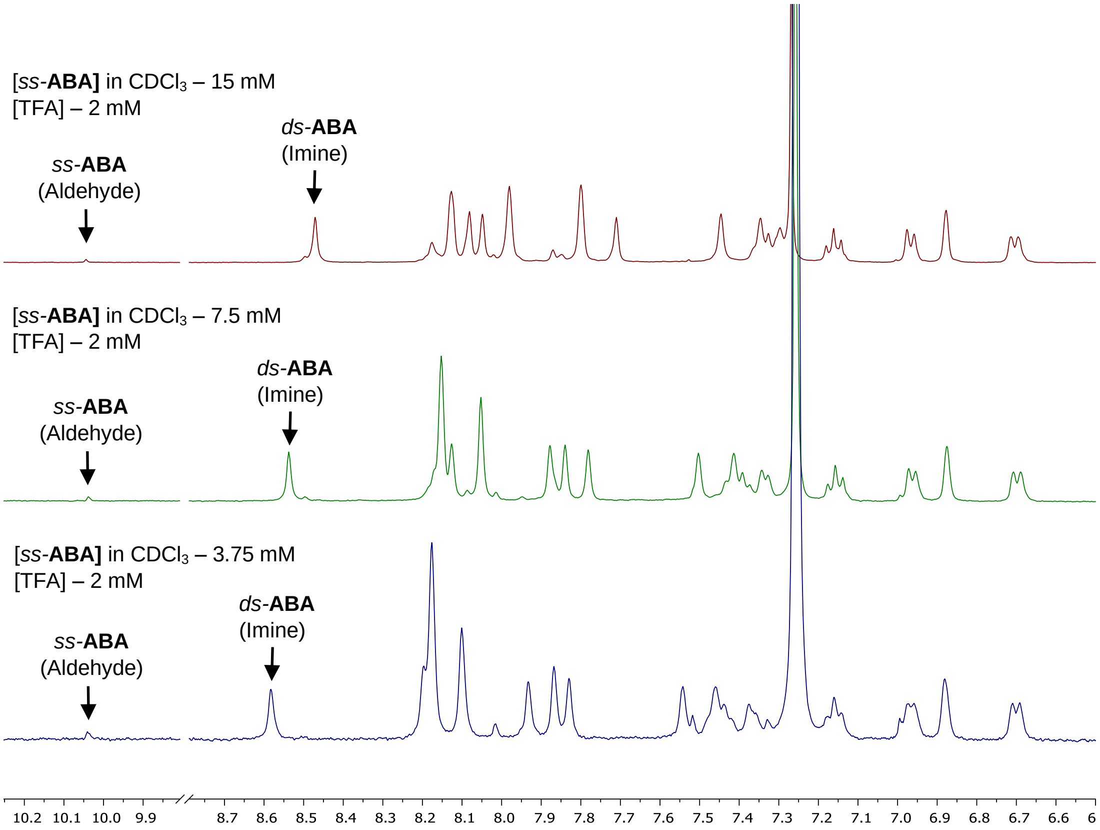

Figure S4. Relevant section of the ^{1}H NMR spectra showing the conversion of *ss*-**ABA** to *ds*-**ABA** after treatment with different amounts of TFA and after quenching the reaction with a 3-fold excess of TEA relative to the [TFA]. The C*H*N imine peaks for *ds*-**ABA** and the C*H*O aldehyde peaks for *ss*-**ABA** are labeled as such.

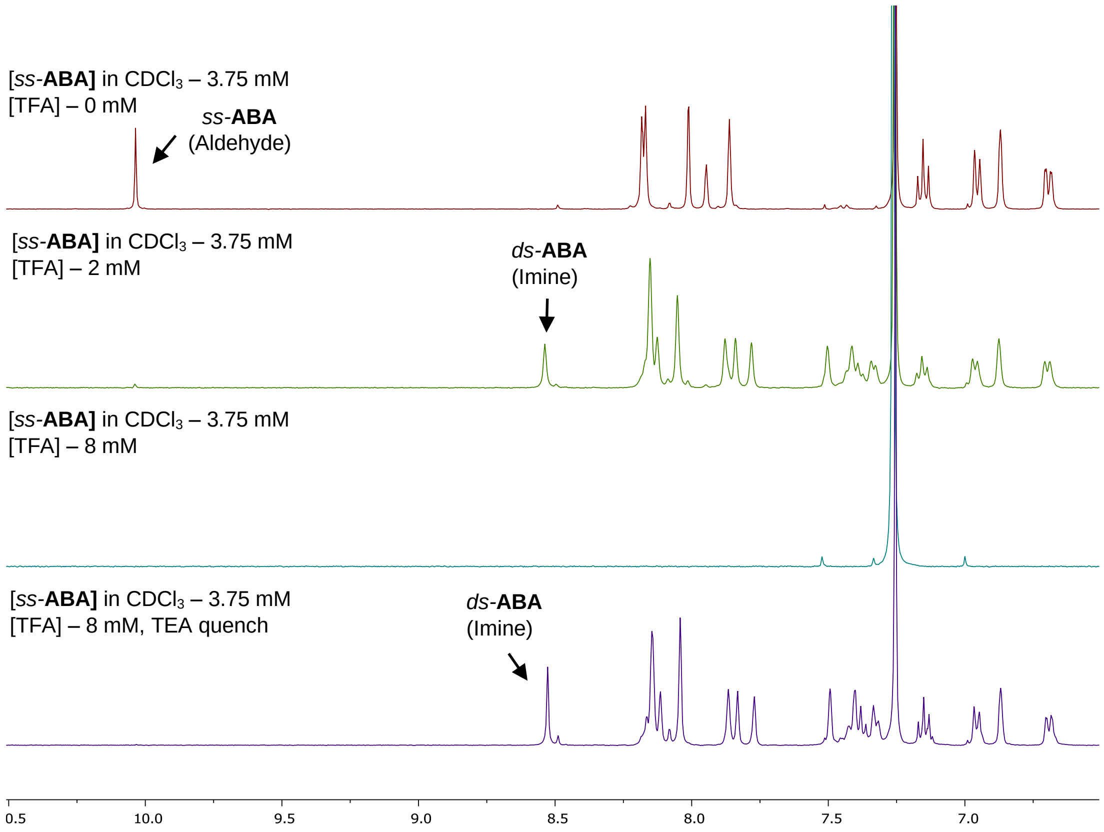

Figure S5. Relevant section of the ^{1}H NMR spectra of **ABB** (4.4 mM) after treatment with different amounts of TFA and after quenching the reaction with a 3-fold excess of TEA relative to the [TFA]. The C*H*N imine peaks for *fold*-**ABB** and *ds*-**ABB** are labeled as such.

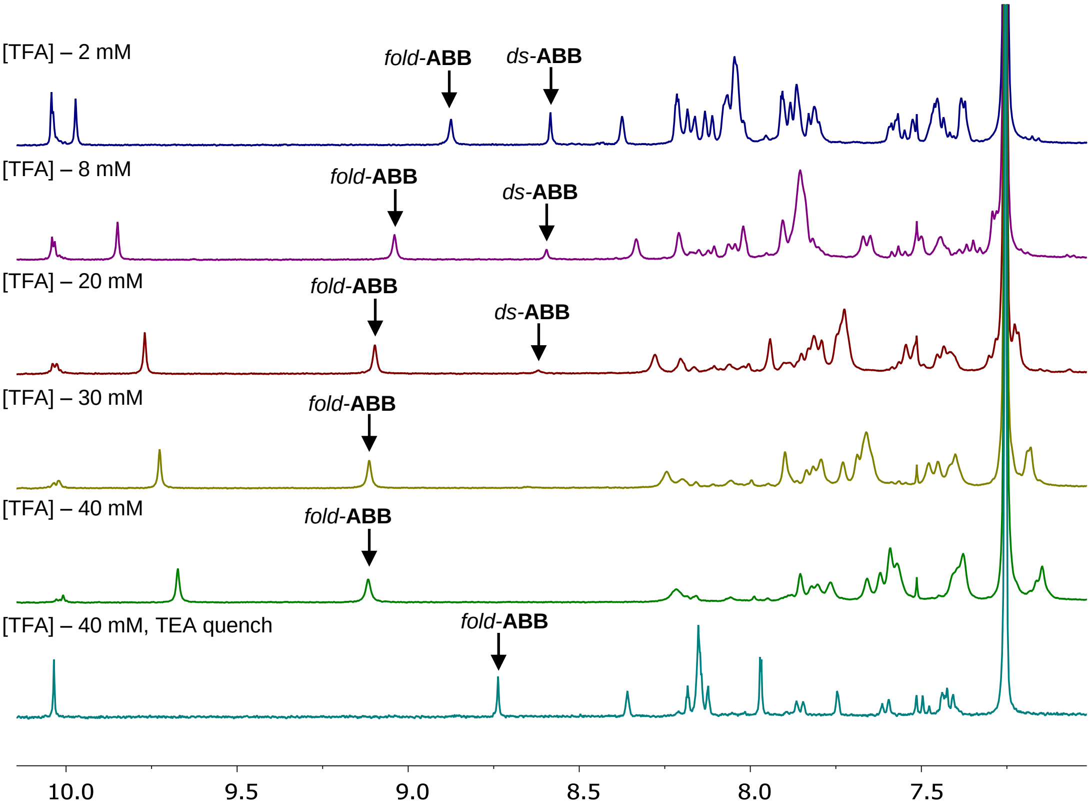

Figure S6. Relevant section of the ^{1}H NMR spectra of **ABB** (17.5 mM) after treatment with different amounts of TFA and after quenching the reaction with a 3-fold excess of TEA relative to the [TFA]. The C*H*N imine peaks for *fold*-**ABB** and *ds*-**ABB** are labeled as such.

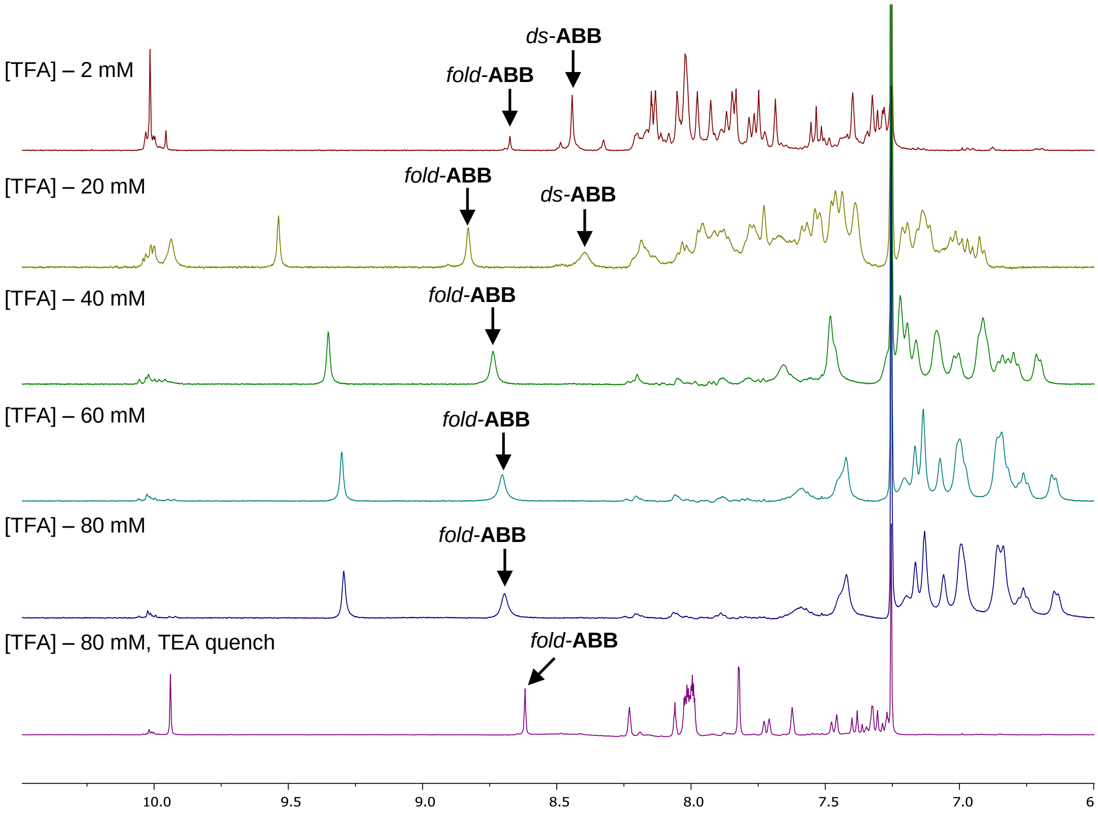

Figure S7. Relevant section of the ^{1}H NMR spectra of *fold*-**ABB** (0.51 mM) in $CDCl_3$ after treatment with different amounts of tetraethylammonium trifluoroacetate (TEA-TFA).

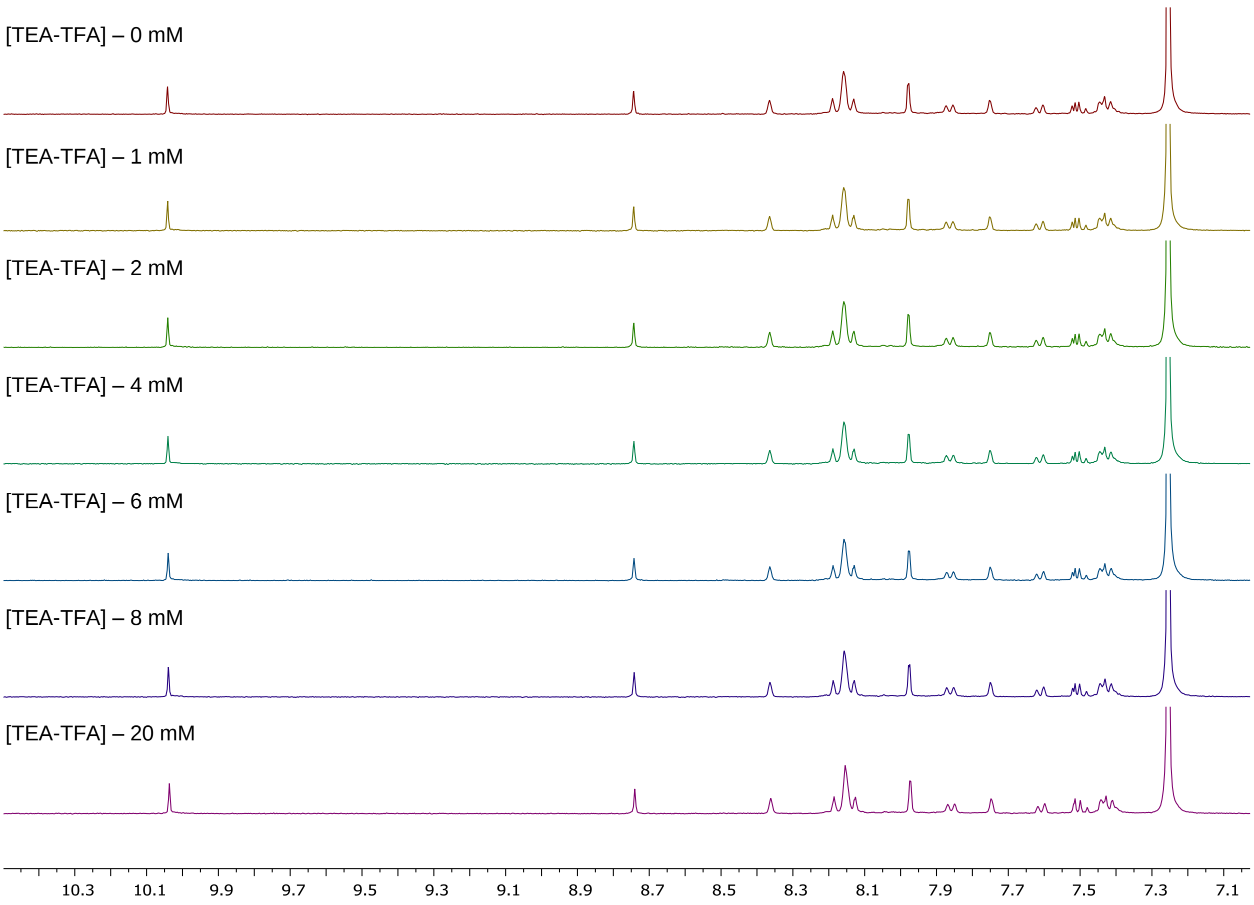

Figure S8. Relevant section of the ^{1}H NMR spectra of *ds*-**ABB** (0.43 mM) in $CDCl_3$ after treatment with different amounts of tetraethylammonium trifluoroacetate (TEA-TFA).

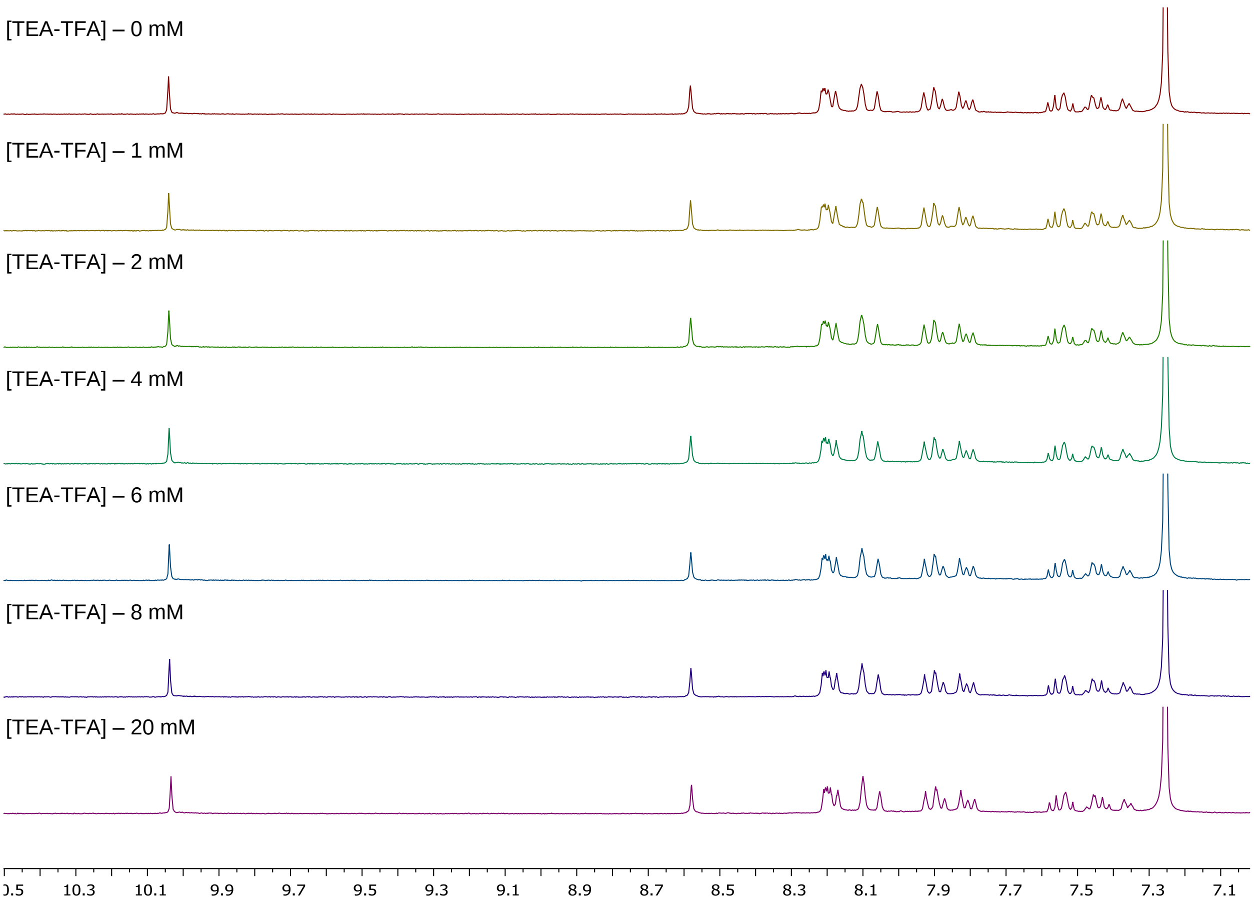

Figure S9. Relevant section of the ^{1}H NMR spectra of **ABB** (0.45 mM) in $CDCl_3$ with 2 mM TFA after treatment with different amounts of tetraethylammonium trifluoroacetate (TEA-TFA). The C*H*N imine peaks for *fold*-**ABB** and *ds*-**ABB** are labeled as such.

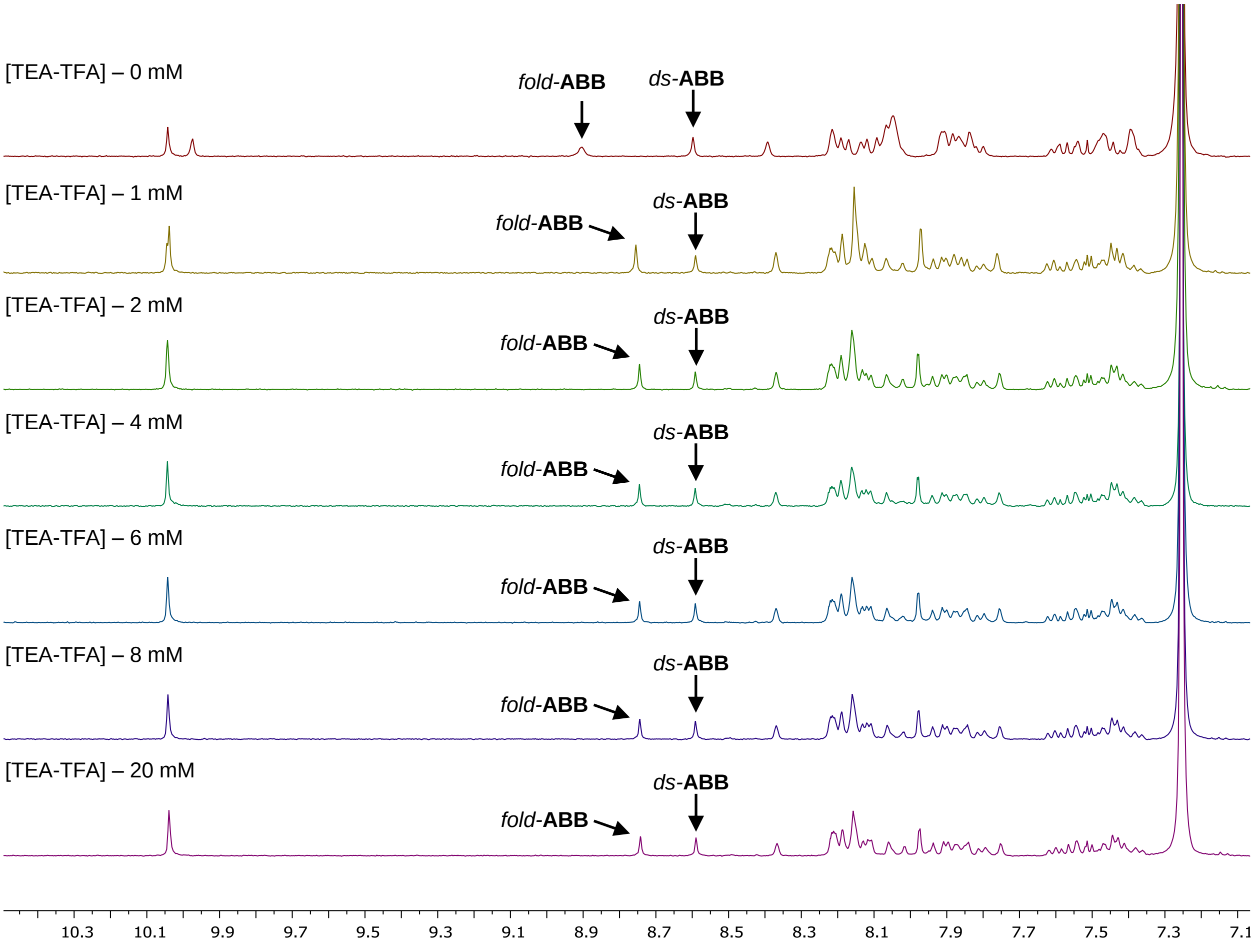

Figure S10. Relevant section of the ^{1}H NMR spectra of **AAB** (3.75 mM) after treatment with different amounts of TFA and after quenching the reaction with a 3-fold excess of TEA relative to the [TFA]. The N*H*C imine peaks for *fold*-**AAB** and *ds*-**AAB** are labeled as such.

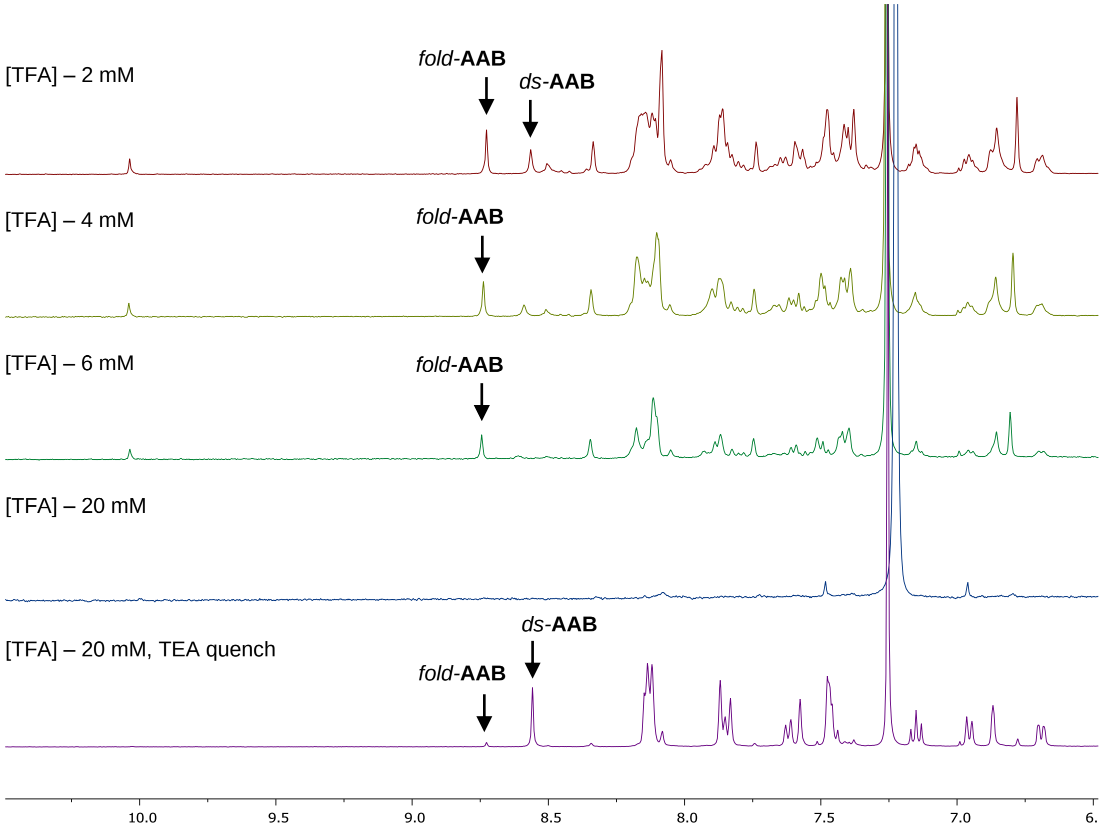

Figure S11. ^{1}H NMR peak assignments and ^{1}H-^{1}H ROSEY and ^{1}H-^{1}H COSY correlations for *ds*-**ABA**. Chemical shifts in ppm are given in parenthesis next to the associated proton. Ambiguous peaks are given as a range where they appear. ^{1}H-^{1}H correlations observed between protons are indicated by a double headed arrow. * denotes peaks where significant overlap occurs in the ^{1}H NMR spectrum, peaks could be assigned with certainty from cross-peaks in the 2D spectra.

^{1}H-^{1}H ROSEY correlations observed for *ds*-**ABA**.

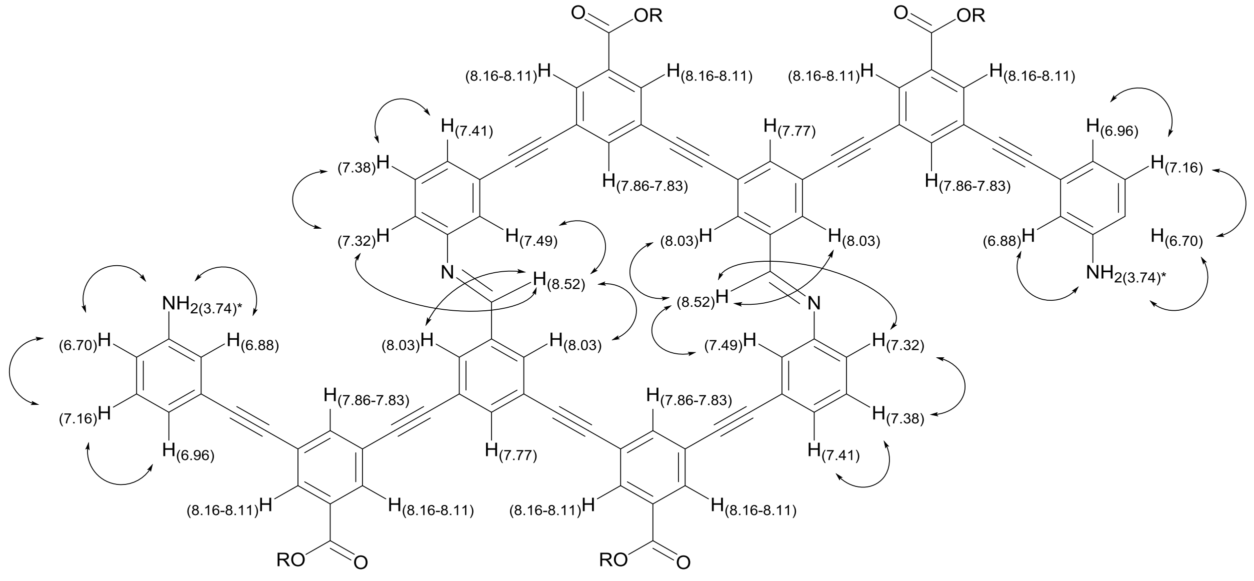

^{1}H-^{1}H COSY correlations observed for *ds*-**ABA**.

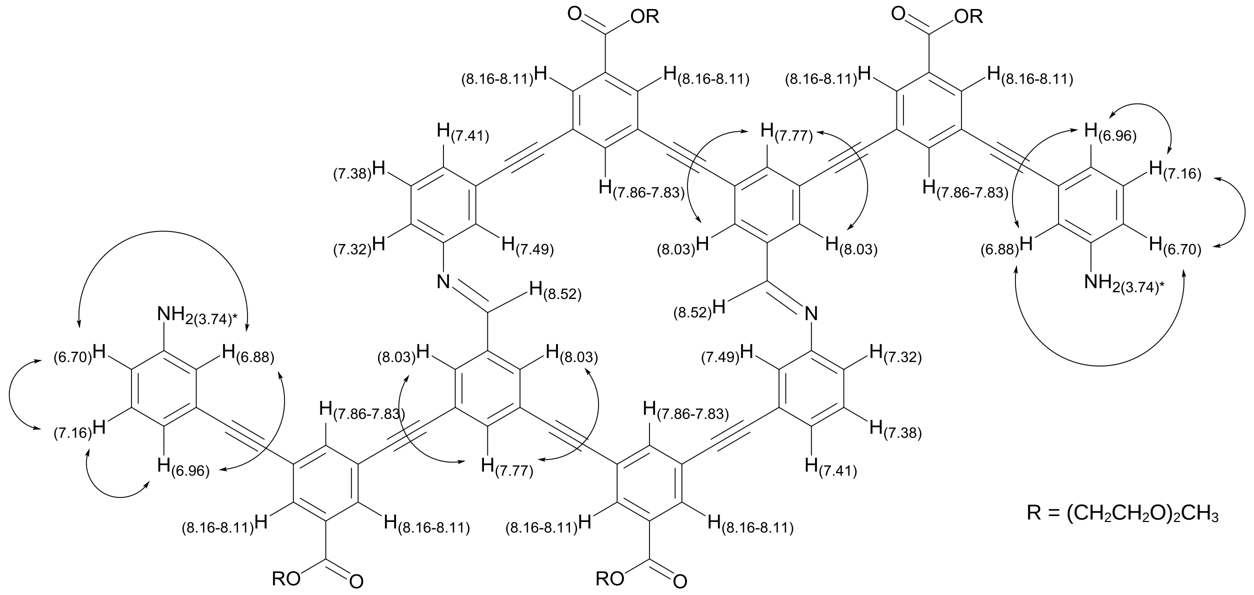

Figure S12. ^{1}H NMR peak assignments and ^{1}H-^{1}H ROSEY and ^{1}H-^{1}H COSY correlations for *ds*-**BAB**. Chemical shifts in ppm are given in parenthesis next to the associated proton. Ambiguous peaks are given as a range where they appear. ^{1}H-^{1}H correlations observed between protons are indicated by a double headed arrow. *denotes peaks where significant overlap occurs in the ^{1}H NMR spectrum, peaks could be assigned with certainty from cross-peaks in the 2D spectra.

^{1}H-^{1}H ROSEY correlations observed for *ds*-**BAB**.

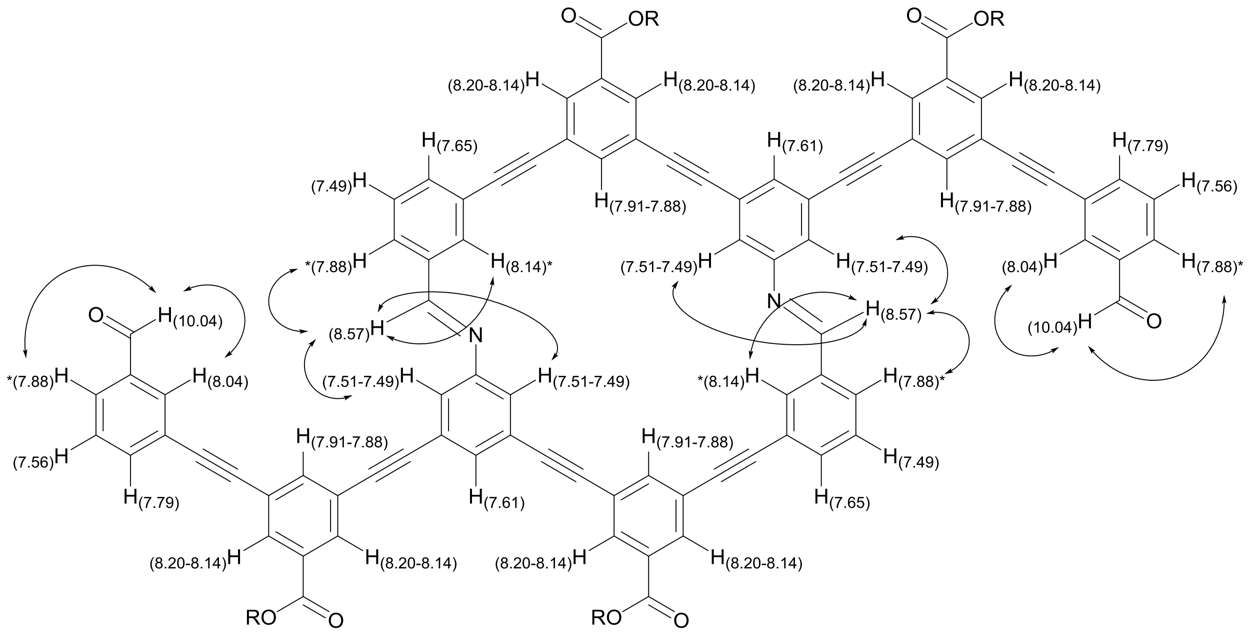

^{1}H-^{1}H COSY correlations observed for *ds*-**BAB**.

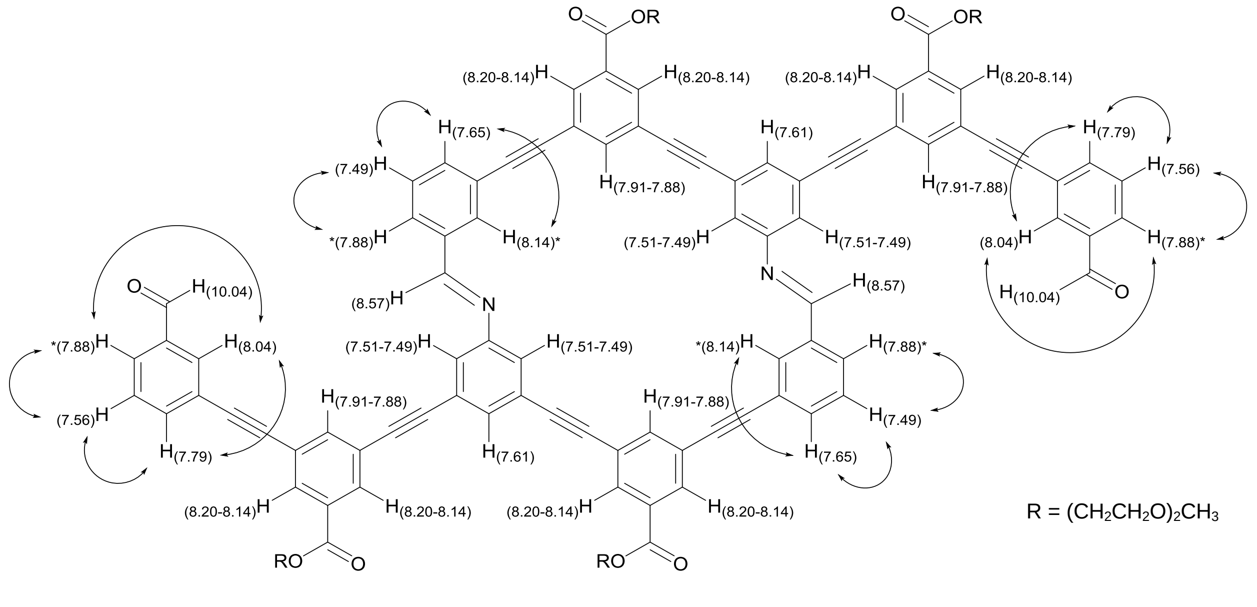

Figure S13. ^{1}H NMR peak assignments and ^{1}H-^{1}H ROSEY and ^{1}H-^{1}H COSY correlations for *ds*-**AAB**. Chemical shifts in ppm are given in parenthesis next to the associated proton. Ambiguous peaks are given as a range where they appear. ^{1}H-^{1}H correlations observed between protons are indicated by a double headed arrow. *denotes peaks where significant overlap occurs in the ^{1}H NMR spectrum, peaks could be assigned with certainty from cross-peaks in the 2D spectra.

^{1}H-^{1}H ROSEY correlations observed for *ds*-**AAB**.

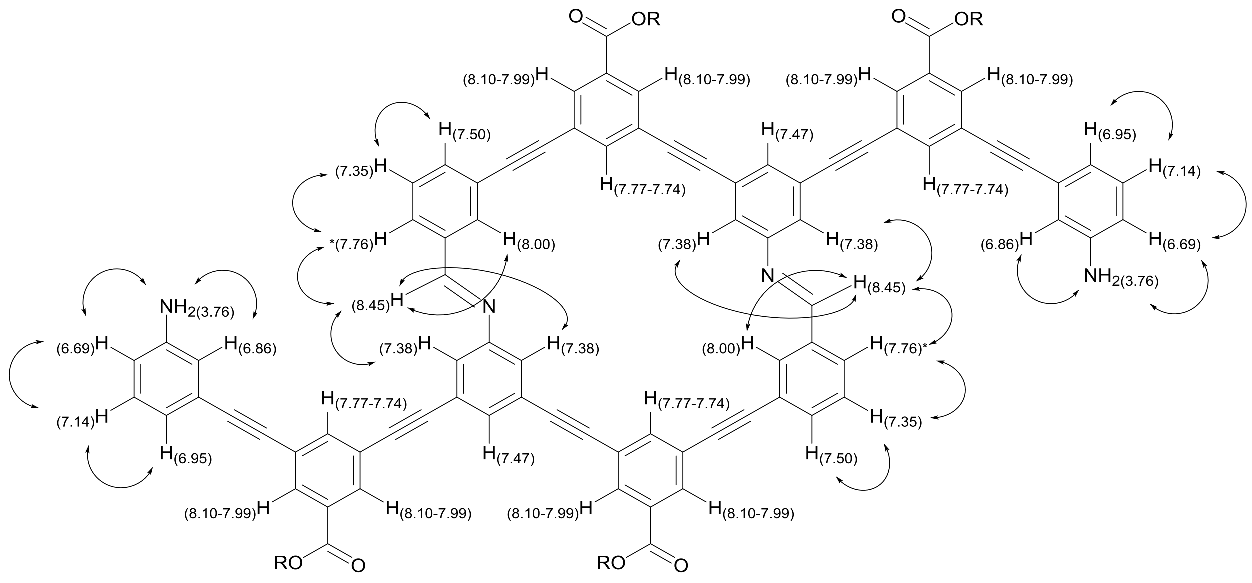

^{1}H-^{1}H COSY correlations observed for *ds*-**AAB**.

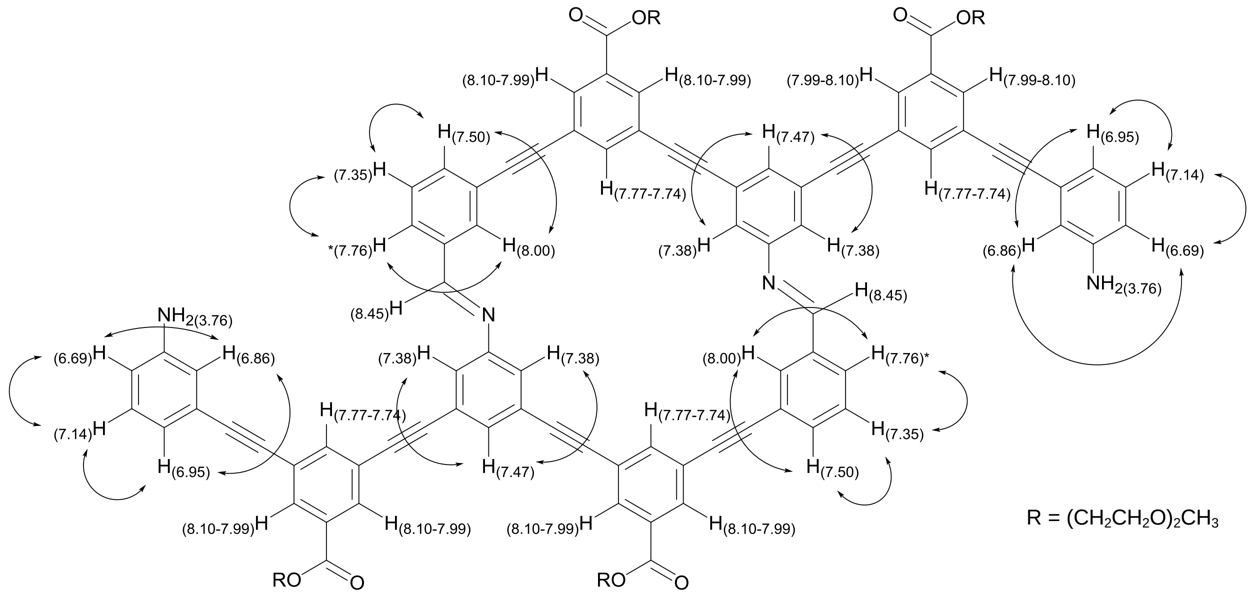

Figure S14. ^{1}H NMR peak assignments and ^{1}H-^{1}H ROSEY and ^{1}H-^{1}H COSY correlations for *ds*-**ABB**. Chemical shifts in ppm are given in parenthesis next to the associated proton. Ambiguous peaks are given as a range where they appear. ^{1}H-^{1}H correlations observed between protons are indicated by a double headed arrow. *denotes peaks where significant overlap occurs in the ^{1}H NMR spectrum, peaks could be assigned with certainty from cross-peaks in the 2D spectra.

^{1}H-^{1}H ROSEY correlations observed for *ds*-**ABB**.

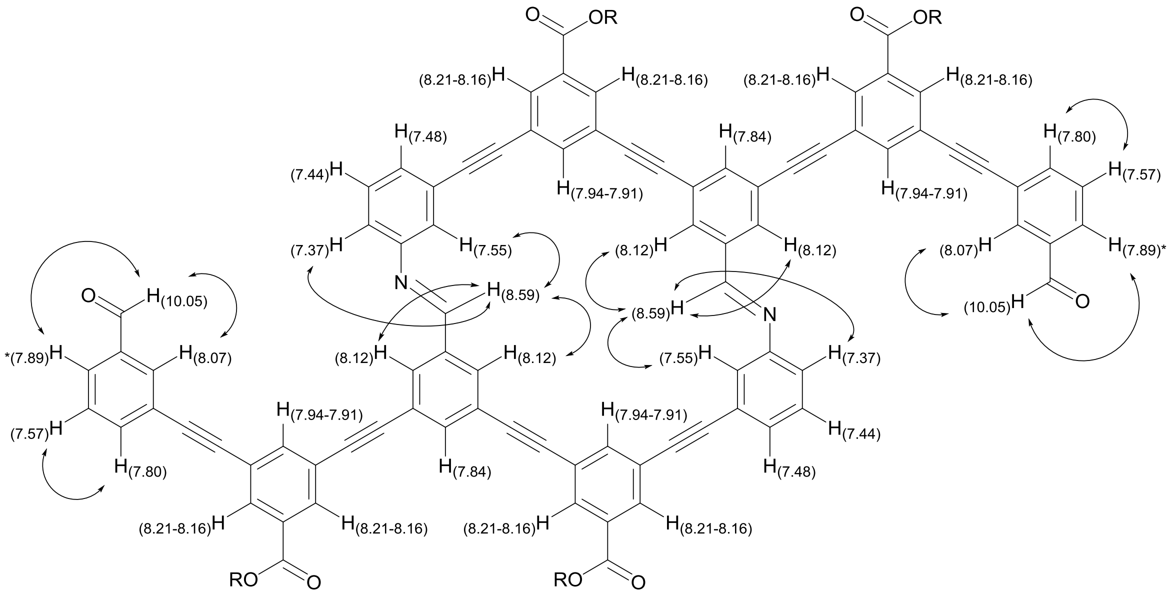

^{1}H-^{1}H COSY correlations observed for *ds*-**ABB**.

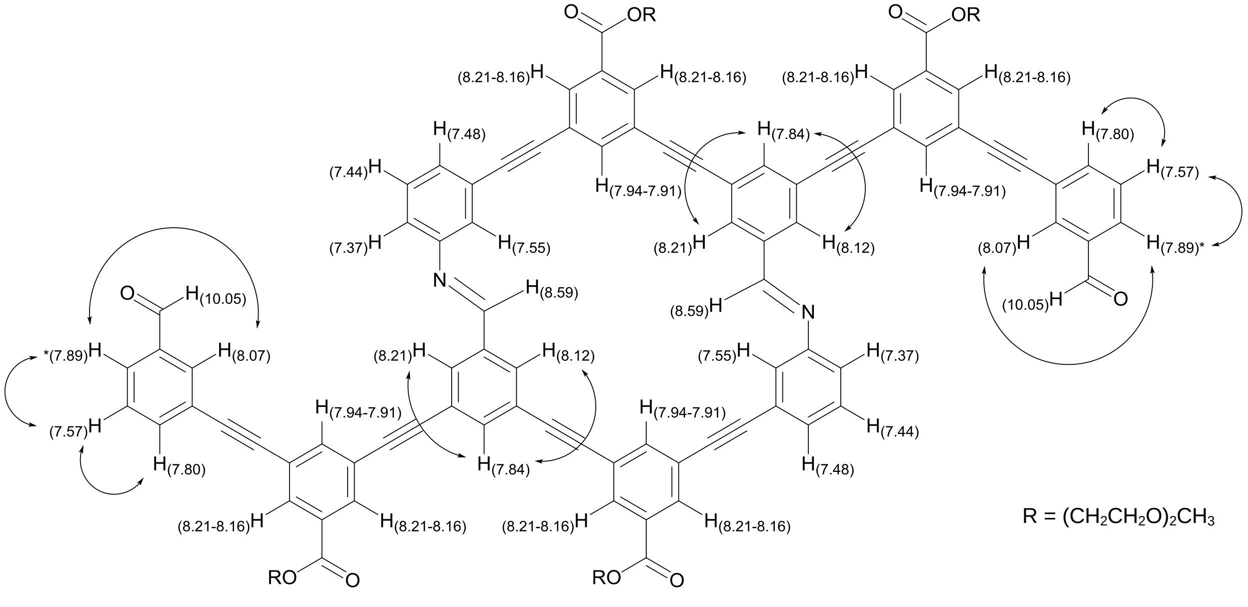

R = $(CH_2CH_2O)_2CH_3$

Figure S15. ^{1}H NMR peak assignments and ^{1}H-^{1}H ROSEY and ^{1}H-^{1}H COSY correlations for *fold*-**AAB**. Chemical shifts in ppm are given in parenthesis next to the associated proton. Overlapping peaks are given as a range where they appear. ^{1}H-^{1}H correlations observed between protons are indicated by a double headed arrow. *denotes peaks where significant overlap occurs in the ^{1}H NMR spectrum, peaks could be assigned with certainty from cross-peaks in the 2D spectra.

^{1}H-^{1}H ROSEY correlations observed for *fold*-**AAB**.

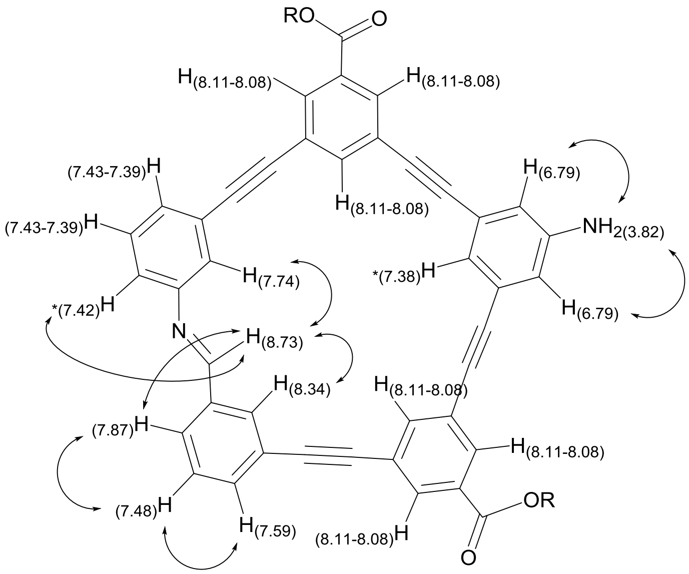

^{1}H-^{1}H COSY correlations observed for *fold*-**AAB**.

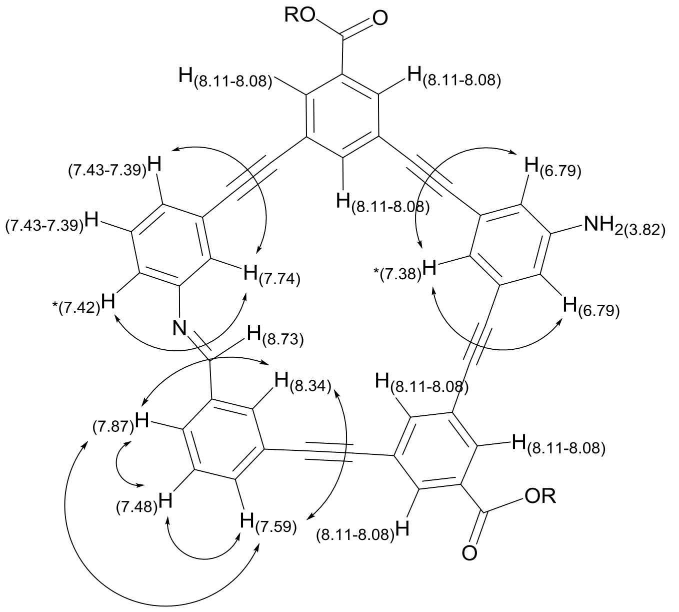

R = $(CH_2CH_2O)_2CH_3$

Figure S16. ^{1}H NMR peak assignments and ^{1}H-^{1}H ROSEY and ^{1}H-^{1}H COSY correlations for *fold*-**ABB**. Chemical shifts in ppm are given in parenthesis next to the associated proton. Overlapping peaks are given as a range where they appear. ^{1}H-^{1}H correlations observed between protons are indicated by a double headed arrow. *denotes peaks where significant overlap occurs in the ^{1}H NMR spectrum, peaks could be assigned with certainty from cross-peaks in the 2D spectra.

^{1}H-^{1}H ROSEY correlations observed for *fold*-**ABB**.

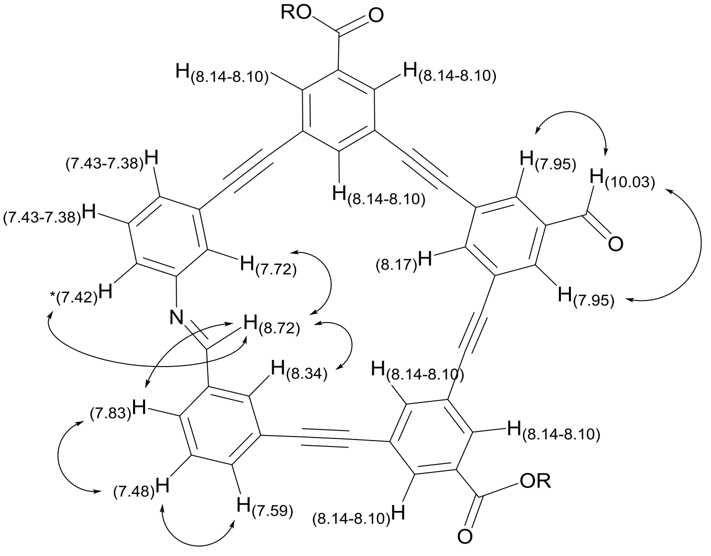

^{1}H-^{1}H COSY correlations observed for *fold*-**ABB**.

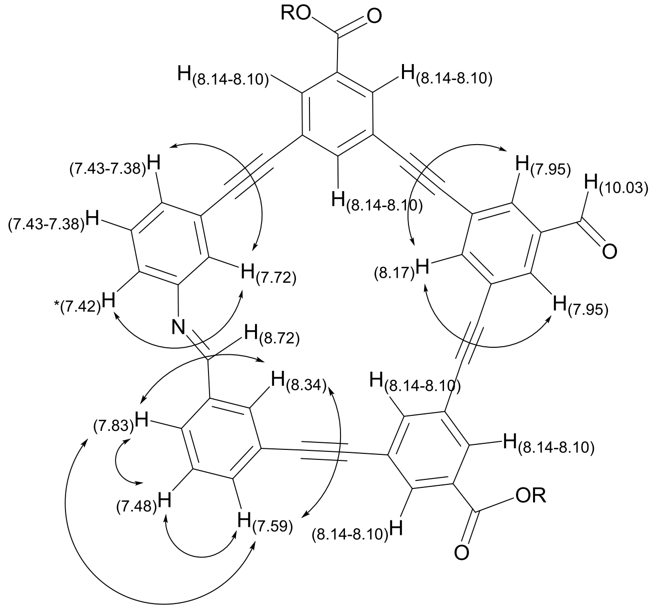

R = $(CH_2CH_2O)_2CH_3$

Figure S17. ESI-TOF Mass spectra for *ss*-ABOs.

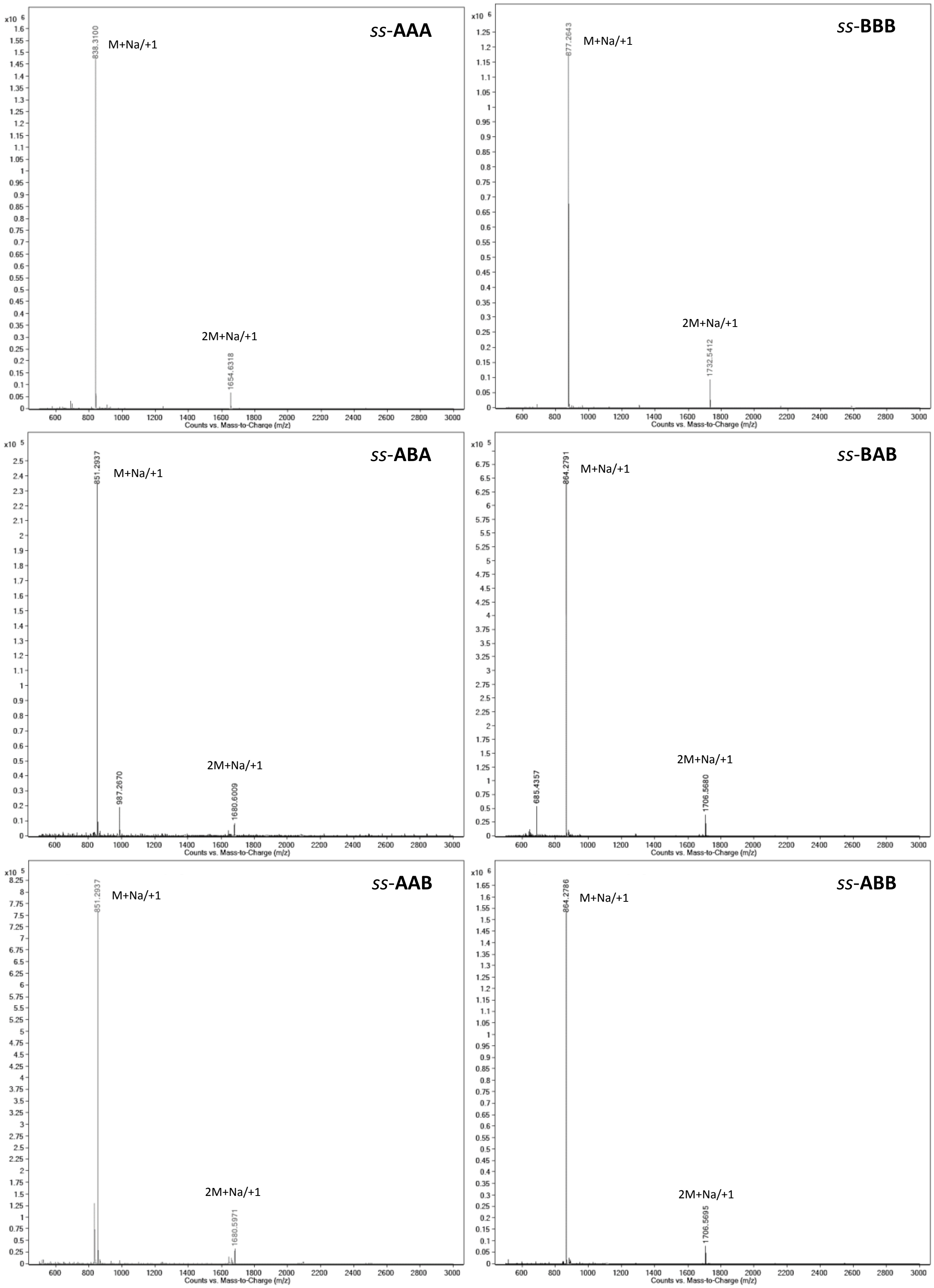

Figure S18. ESI-TOF Mass spectra for *ds*-, and *fold*-ABOs. [a]These peaks are distinguishable from the corresponding *fold*-ABOs by their isotopic ratios indicating a +2 charge state. [b]These peaks are not distinguishable from the M+Na/+1 ions of the corresponding *ds*-ABOs, however NMR data confirms these solutions do not contain significant amounts of *ds*-ABO and so it can be concluded that these peaks arise from 2M+Na/+1 ions of the *fold*-ABO.

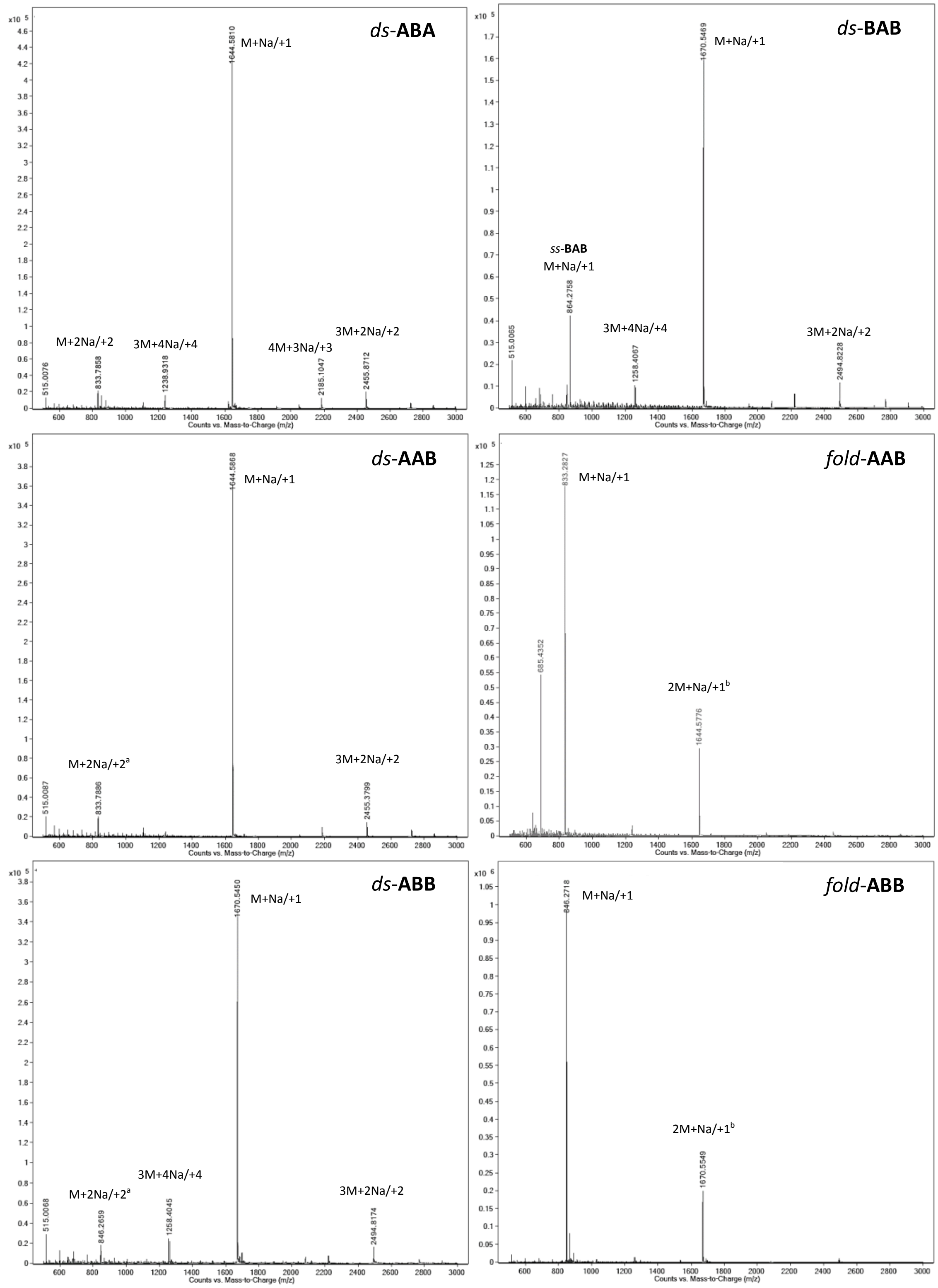

Figure S19. Relevant section of the ^{1}H NMR spectra of *ds*-**AAA·BBB** in $CDCl_3$ over a dilution series from 480 uM to 30 uM. Spectra acquired at 50 °C.

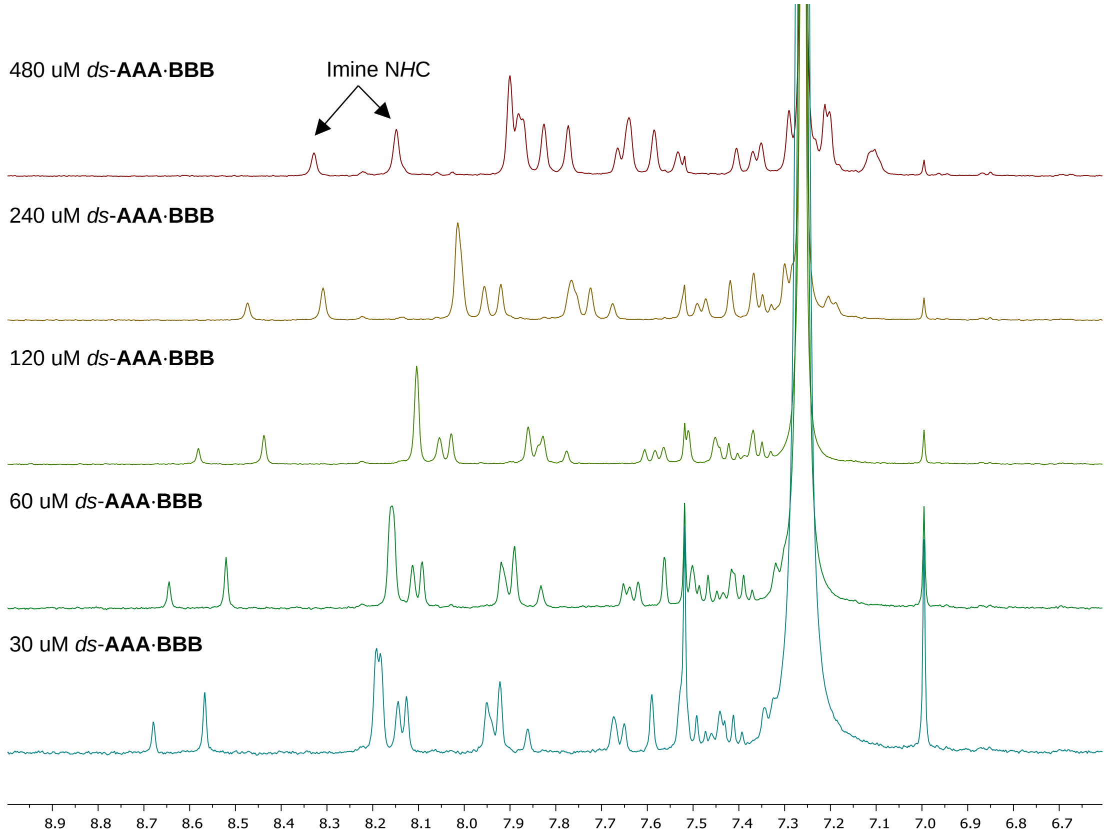

Figure S20. Relevant section of the ^{1}H NMR spectra of *ds*-**ABA·BAB** in $CDCl_3$ over a dilution series from 383 uM to 3 uM. Spectra acquired at rt.

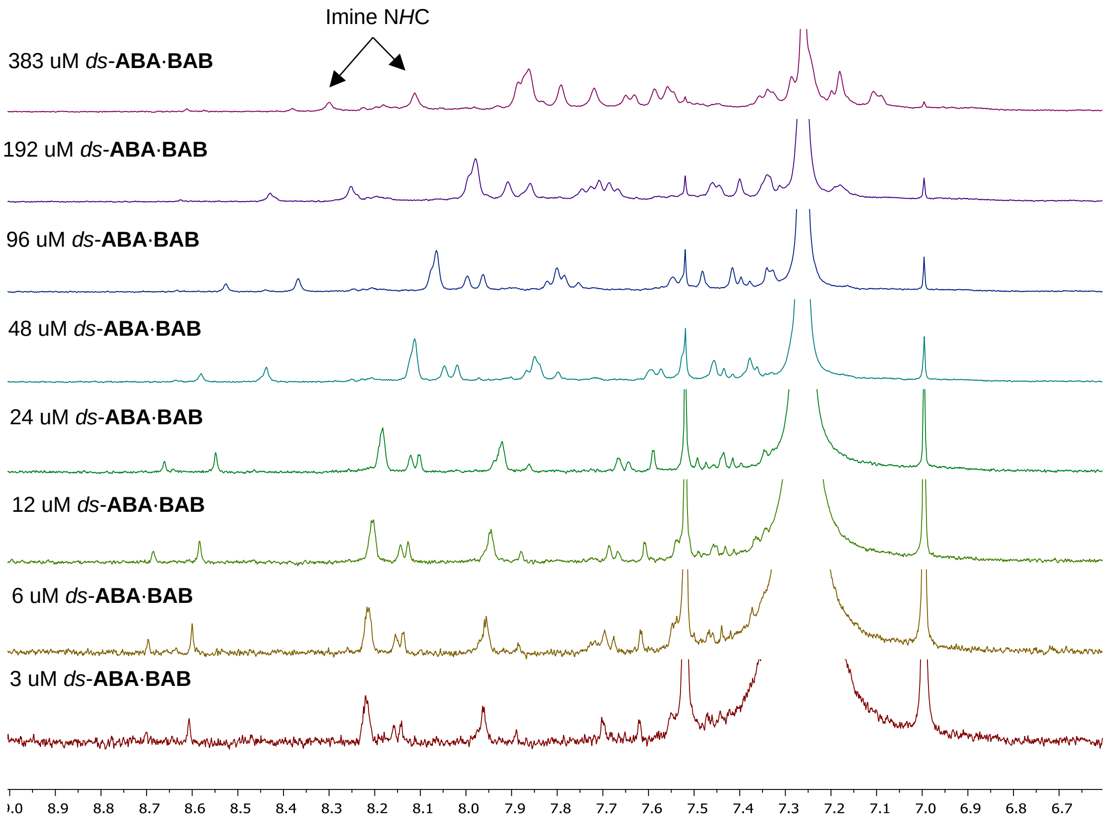

Figure S21. Relevant section of the ^{1}H NMR spectra of *ds*-**ABA·BAB** in $CDCl_3$ over a dilution series from 383 uM to 3 uM. Spectra acquired at 50 °C.

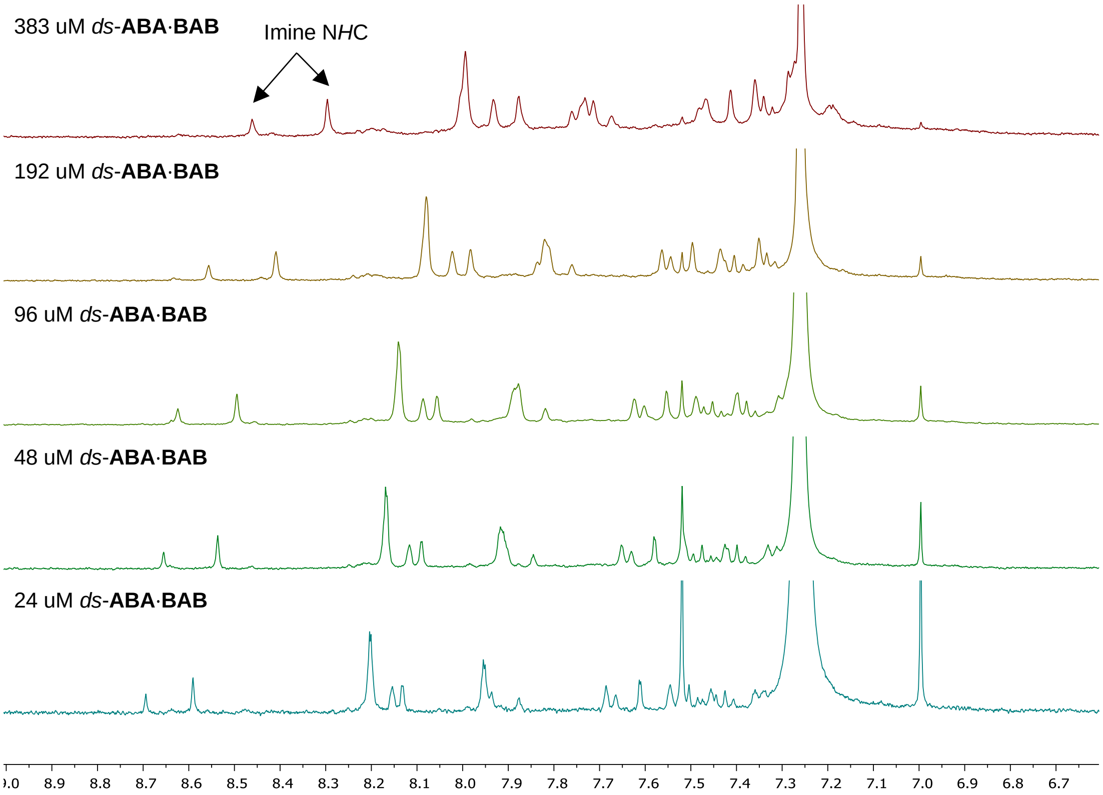

Figure S22. Relevant section of the ^{1}H NMR spectra of *ds*-**AAB·BBA** in $CDCl_3$ over a dilution series from 259 uM to 2 uM. Spectra acquired at rt.

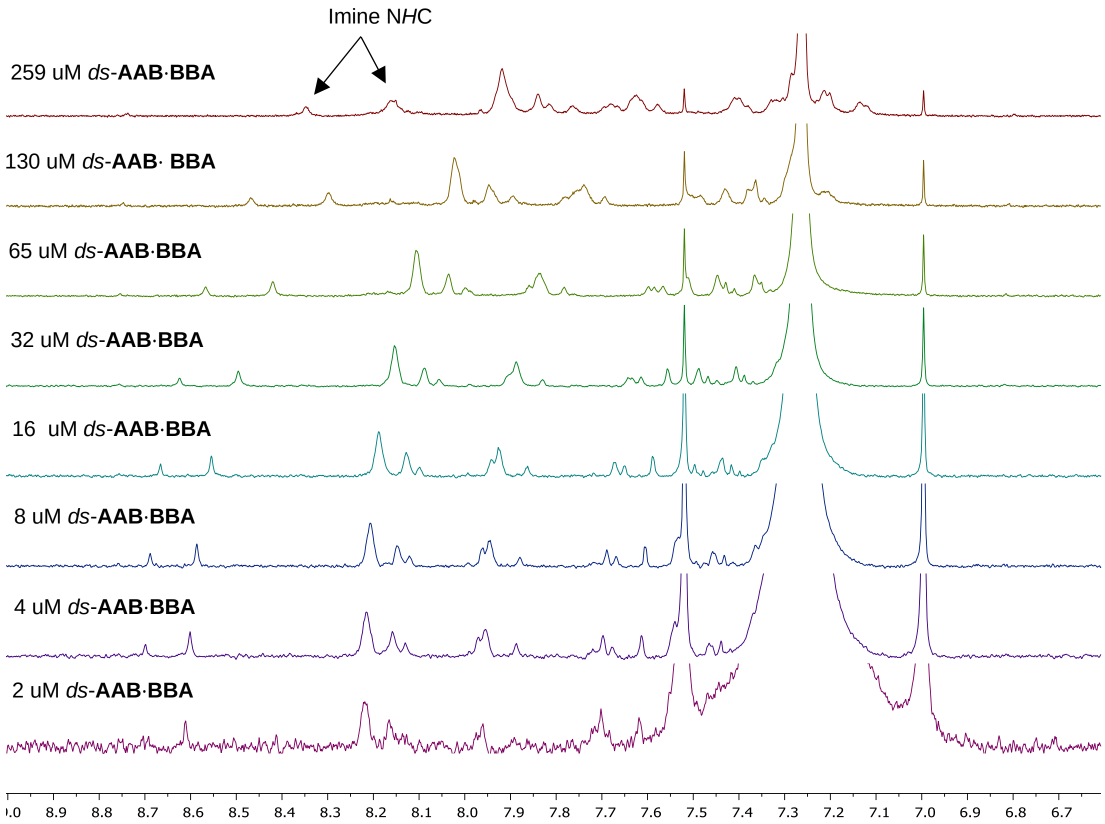

Figure S23. Relevant section of the ^{1}H NMR spectra of *ds*-**AAB·BBA** in $CDCl_3$ over a dilution series from 259 uM to 2 uM. Spectra acquired at 50 °C.

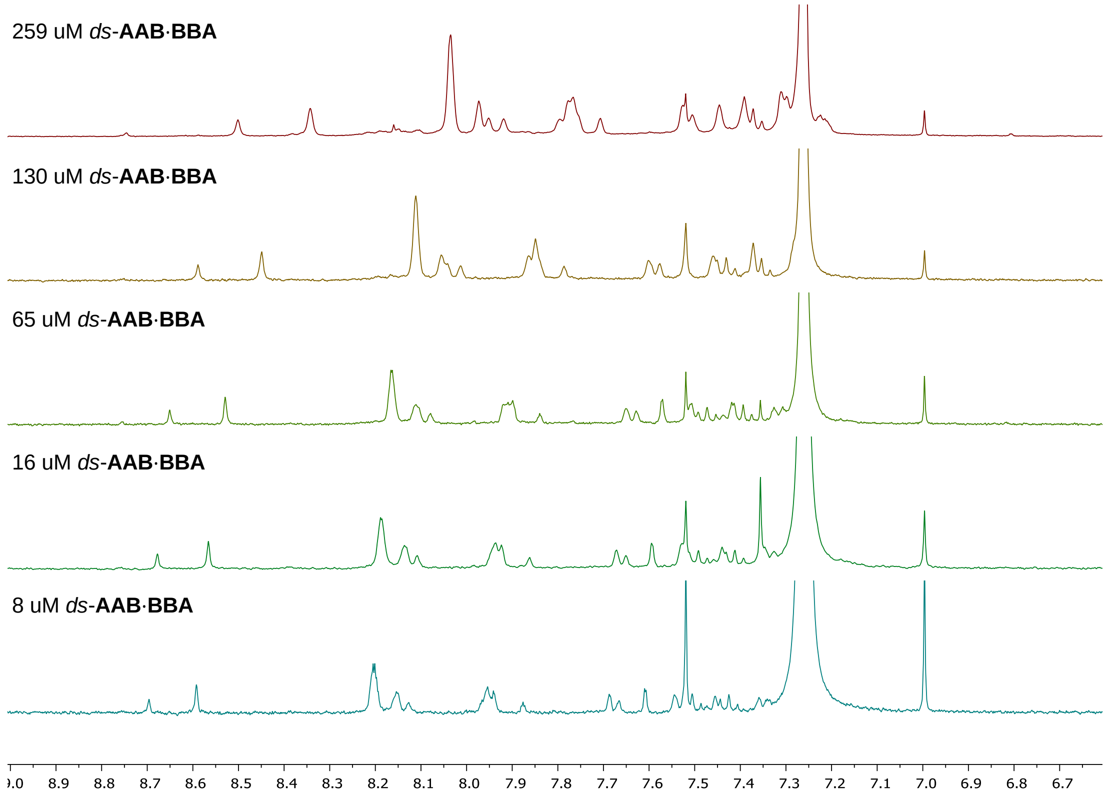

Figure S24. MALDI-TOF Mass spectra for *ds*-**AAA·BBB**, *ds*-**ABA·BAB**, and *ds*-**AAB·ABB**.

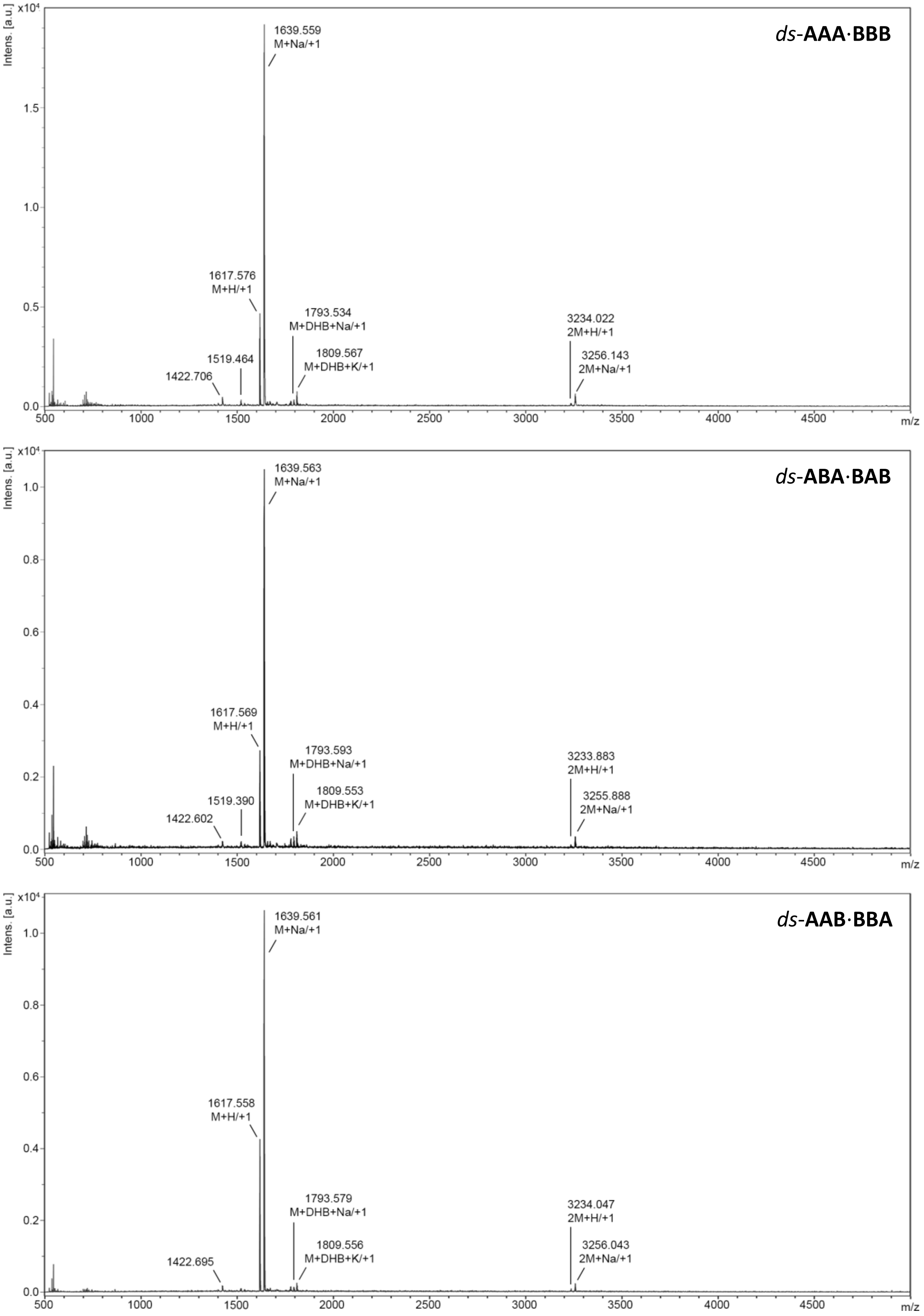

Figure S25. Concentration dependence of the ^{1}H NMR chemical of shift of 3-rung duplexes in $CDCl_3$ at rt and 50 °C for three resonances: blue, the internal imine (NHC), red the terminal imines (NHC), and yellow the aromatic protons meta to the ester functional groups. Curves represent the line of best fit to the equal K model of indefinite association. The error is the standard error estimated from the variance-covariance matrix of the least-squares fitted parameters.

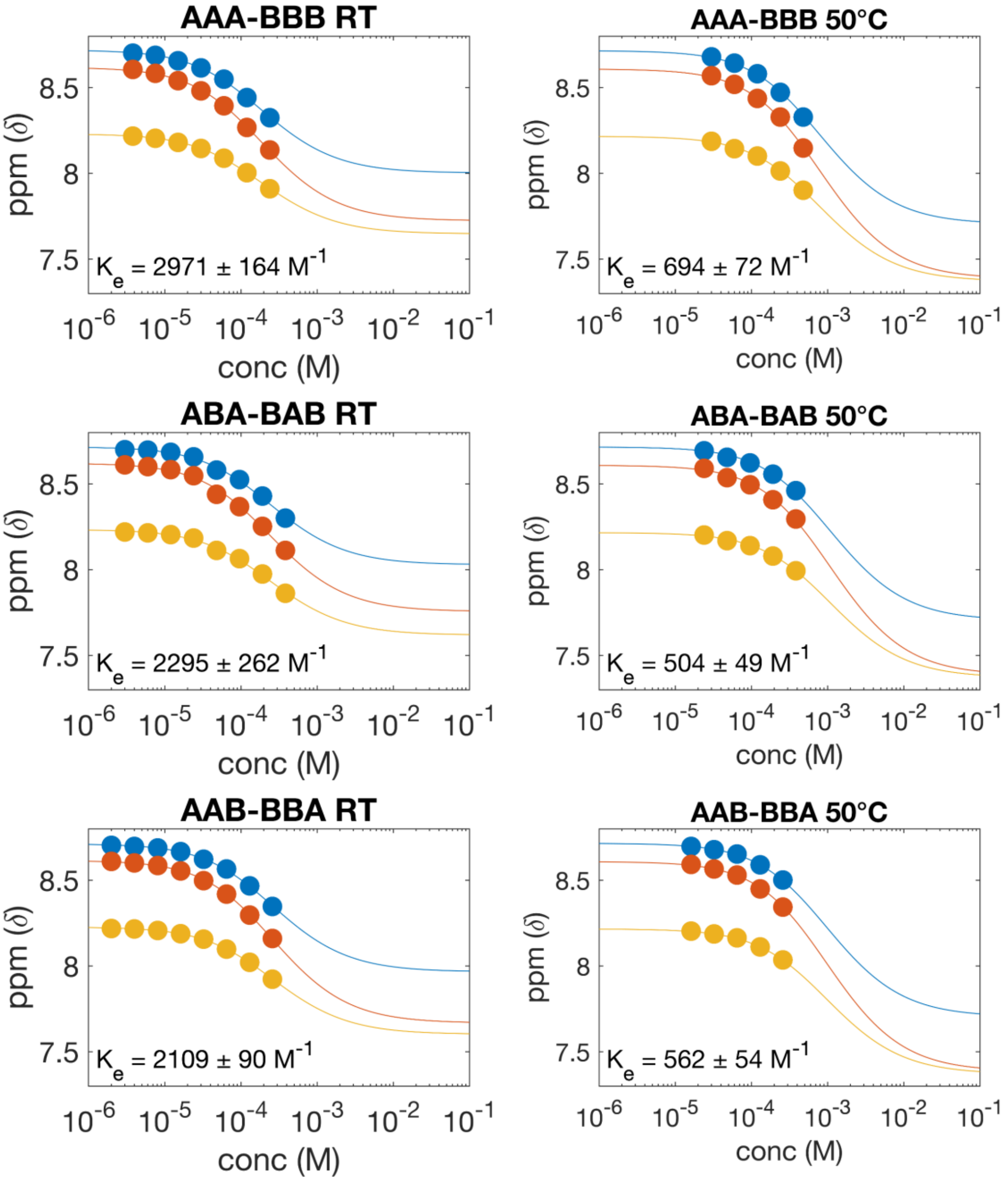

EXPERIMENTAL SECTION

General. All solvents and catalysts, as well as starting materials 3-ethnylbenzlaldhye **1** and 3-ethynylaniline **4** were purchased from commercial sources and used without further treatment. Diiodide **2**,[1] 3,5-diethnylbenzalehyde,[2] and 3,5-diethnylaniline[3] were prepared via previously reported methods. "Dry" $CDCl_3$ was prepared by treating and storing commercially available $CDCl_3$ over 4 Å molecular sieves. THF was purchased as a solution stabilized with 250 ppm BHT and fractionally distilled before use. Removal of peroxides from the THF was crucial to the solution stability of the ABOs. MM2 energy minimum calculations for 1,3-folded **AAB** and 1,2-folded **AAB** were accomplished using the Perkin Elmer Chem3D software package. ^{1}H NMR and ^{13}C NMR spectra were recorded at 93.94 kG (^{1}H 400 MHz, ^{13}C 100 MHz) at 25 °C. Hydrogen chemical shifts are expressed in parts per million (ppm) relative to the residual protio-solvent resonance: $CDCl_3$ δ 7.26. For ^{13}C spectra, the centerline of the solvent signal was used as internal reference: $CDCl_3$ δ 77.16. Unless otherwise noted, each carbon resonance represents a single carbon (relative intensity). ESI-TOF high resolution mass spectrometric data were obtained on a ToF (time-of-flight) Agilent Technologies system. Samples were injected as 10 uM solutions in 8:2 THF:H_2O with 20 uM sodium acetate. The MS settings were: capillary voltage 4500 V, desolvation temperature 300 °C, fragmentor voltage 450 V. Matrix-assisted laser desorption/ionization (MALDI) time-of-flight (TOF) mass spectrometry experiments were carried out as follows: 0.5 uL of a 100 uM solution of 2,5-dihydroxybenzoic acid matrix (Sigma) in methanol was deposited on a MTP 384 polished steel BC target plate (Bruker), and the solvent was allowed to evaporate. A single 0.5 µL aliquot of analyte in $CDCl_3$ was then added on top of the 2,5- dihydroxybenzoic acid film and the sample analyzed with an autoflextreme MALDI-TOF mass spectrometer (Bruker). For K_e determination, a global fit of three protons to the equal K model of indefinite association was preformed using the MATLAB software package the multiple curve fitting with common parameters tool. [4]

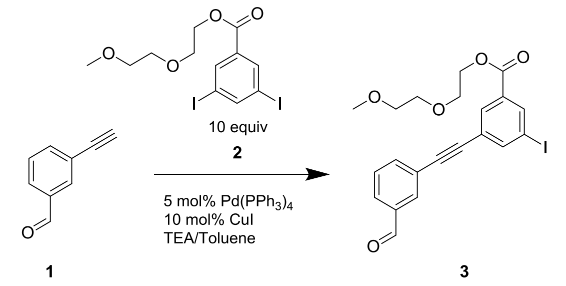

2-(2-methoxyethoxy)ethyl 3-((3-formylphenyl)ethynyl)-5-iodobenzoate (**3**).

To a 100 mL round bottom flask equipped with a stir bar **1** (65 mg, 0.5 mmol) was added then dissolved in anhydrous toluene (5 mL) and DIPEA (3 equiv. 1.5 mmol, 261 uL). Diiodide **2** was added (10 equiv., 5 mmol, 2.38 g) to the reaction vessel and the solution stirred until homogeneous (approx. 15 minutes). To the stirred solution $Pd(PPh_3)_4$ (0.05 equiv., 0.025 mmol, 29 mg) and

CuI (0.1 equiv., 0.05 mmol, 9.5 mg) were then added. The headspace of the reaction vessel was purged with argon and the flask sealed with a glass stopper. After stirring 2.5 h at room temperature dry silica was added. The mixture was then concentrated onto the silica gel using rotoevaporation and the resulting white powder used to dry load a silica gel column. Normal phase silica gel flash chromatography (40 g silica column, hexane with a 20-60% gradient of EtOAc over 18 minutes) gave product **3** (227 mg, 0.47 mmol, 95% yield) as a white solid and recovered unreacted **2** (2.05 g, 96%). ^{1}H NMR (400 MHz, $CDCl_3$) δ 10.03 (s, 1H), 8.36 (t, *J* = 1.6 Hz, 1H), 8.18 (t, *J* = 1.5 Hz, 1H), 8.06 (t, *J* = 1.6 Hz, 1H), 8.03 (t, *J* = 1.7 Hz, 1H), 7.88 (dt, *J* = 7.7, 1.4 Hz, 1H), 7.77 (dt, *J* = 7.8, 1.4 Hz, 1H), 7.55 (t, *J* = 7.7 Hz, 1H), 4.55 – 4.47 (m, 2H), 3.88 – 3.82 (m, 2H), 3.73 – 3.67 (m, 2H), 3.61 – 3.55 (m, 2H), 3.40 (s, 3H). ^{13}C NMR (100 MHz, $CDCl_3$) δ 193.9, 166.9, 146.6, 141.0, 139.7, 139.1, 135.5, 134.6, 134.5, 132.1, 131.9, 127.5, 126.2, 96.0, 92.6, 90.8, 74.5, 73.1, 71.7, 67.3, 61.7. HRMS (ESI): m/z calcd for $C_{21}H_{19}IO_5$+Na^+ 501.0169 [M+Na]$^+$; found 501.0145.

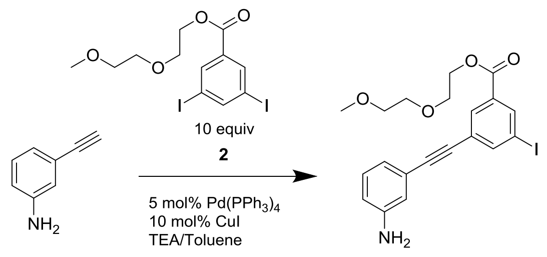

2-(2-methoxyethoxy)ethyl 3-((3-aminophenyl)ethynyl)-5-iodobenzoate (**5**).

To a 100 mL round bottom flask equipped with a stir bar **4** (56 uL, 0.5 mmol) was added then dissolved in anhydrous toluene (5 mL) and DIPEA (3 equiv. 1.5 mmol, 261 uL). Diiodide **2** was added (10 equiv., 5 mmol, 2.38 g) to the reaction vessel and the solution stirred until homogeneous (approx. 15 minutes). To the stirred solution $Pd(PPh_3)_4$ (0.05 equiv., 0.025 mmol, 29 mg) and CuI (0.1 equiv., 0.05 mmol, 9.5 mg) were then added. The headspace of the reaction vessel was purged with argon and the flask sealed with a glass stopper. After stirring 2.5 h at room temperature dry silica was added. The mixture was then concentrated onto the silica gel using rotoevaporation and the resulting white powder used to dry load a silica gel column. Normal phase silica gel flash chromatography (40 g silica column, hexane with a 30-70% gradient of EtOAc over 18 minutes) gave product **5** (219 mg, 0.47 mmol, 94% yield) as a white solid and recovered unreacted **2** (2.07 g, 96%). ^{1}H NMR (400 MHz, $CDCl_3$) δ 8.29 (t, *J* = 1.6 Hz, 1H), 8.12 (t, *J* = 1.5 Hz, 1H), 8.00 (t, *J* = 1.6 Hz, 1H), 7.12 (t, *J* = 7.8 Hz, 1H), 6.91 (dt, *J* = 7.6, 1.2 Hz, 1H), 6.81 (dd, *J* = 2.4, 1.4 Hz, 1H), 6.66 (ddd, *J* = 8.0, 2.4, 1.0 Hz, 1H), 4.51 – 4.45 (m, 2H), 3.86 – 3.79 (m, 2H), 3.74 (br, 2N*H*), 3.71 – 3.64 (m, 2H), 3.59 – 3.53 (m, 2H), 3.38 (s, 3H). ^{13}C NMR (100 MHz, $CDCl_3$) δ 164.6, 146.5, 144.1, 137.9, 132.0, 131.9, 129.4, 125.7, 123.1, 122.1, 117.8, 115.9, 93.3, 92.1, 86.1, 71.9, 70.6, 69.1, 64.6, 59.2. HRMS (ESI): m/z calcd for $C_{20}H_{20}INO_4$+H^+: 466.0510 [M+H]$^+$; found 466.0531.

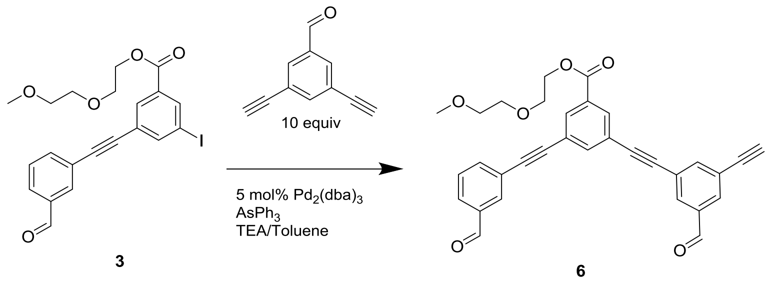

2-(2-methoxyethoxy)ethyl 3-((3-ethynyl-5-formylphenyl)ethynyl)-5-((3-formylphenyl)ethynyl)benzoate (**6**).

To a 20 mL scintillation vial $AsPh_3$ (0.04 mmol, 12 mg) and $Pd_2(dba)_3$ (0.01 mmol, 5.0 mg, 5.0 mol%) were added and dissolved in anhydrous toluene (1 mL). The head space of the vial was purged with argon then the vial sealed with a screw on cap. The purple solution was gently swirled until the $Pd_2(dba)_3$ dissolved and the color changed to bright yellow (approx. 10 min). In a separate vial equipped with a stir bar, 2,6-diethynylbenzaldehyde (308 mg, 2.0 mmol, 10 equiv.) and aryl-iodide **3** (1 equiv., 96 mg, 0.2 mmol) were dissolved in anhydrous toluene (1 mL) and DIPEA (12 equiv., 2.4 mmol, 414 uL). While stirring under a stream of argon the Pd catalyst solution was added dropwise (1 mL) to the solution containing the aldehyde and alkyne. The vessel was sealed with a screw on cap and heated to 40 °C. After stirring overnight, the reaction was cooled to rt transferred to a round bottom flask and diluted with DCM (approx. 50 mL). Silica gel was added to vessel and crude reaction mixture concentrated onto the silica gel. Silica gel flash chromatography (hexane with a 20-90% gradient of EtOAc over 18 minutes) gave pure **6** (86 mg, 0.17 mmol, 85%) as a white solid. ^1H NMR (400 MHz, $CDCl_3$) δ 10.04 (s, 1H), 10.00 (s, 1H), 8.21 (d, *J* = 1.6 Hz, 1H), 8.19 (t, *J* = 1.6 Hz, 1H), 8.05 (t, *J* = 1.7 Hz, 1H), 8.00 (t, *J* = 1.6 Hz, 1H), 7.96 (t, *J* = 1.6 Hz, 1H), 7.92 – 7.86 (overlap, 3H), 7.79 (dt, *J* = 7.7, 1.5 Hz, 1H), 7.56 (t, *J* = 7.7 Hz, 1H), 4.56 – 4.51 (m, 2H), 3.90 – 3.85 (m, 2H), 3.75 – 3.69 (m, 2H), 3.61 – 3.57 (m, 2H), 3.40 (s, 3H), 3.21 (s, 1H). ^{13}C NMR (100 MHz, $CDCl_3$) δ 191.5, 190.6, 165.1, 140.2, 138.5, 137.3, 136.8, 136.7, 133.2, 133.1, 133.0, 132.9, 132.7, 131.2, 129.6, 129.4, 124.4, 124.0, 123.93, 123.89, 123.5, 89.75, 89.69, 89.0, 88.6, 81.4, 79.8, 72.1, 70.7, 69.3, 64.7, 59.3. HRMS (ESI): m/z calcd for $C_{32}H_{24}O_6$+Na^+: 527.1465 $[M+Na]^+$; found 527.1443.

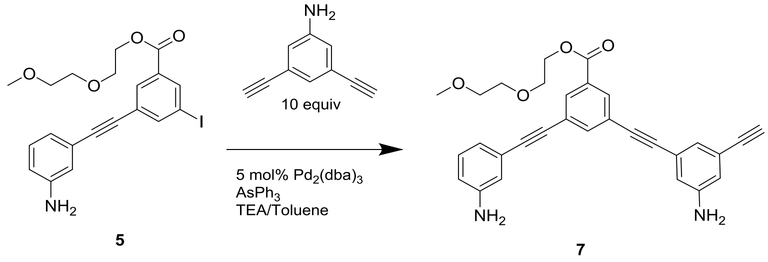

2-(2-methoxyethoxy)ethyl 3-((3-amino-5-ethynylphenyl)ethynyl)-5-((3-aminophenyl)ethynyl)benzoate (**7**).

To a 20 mL scintillation vial $AsPh_3$ (0.046 mmol, 14.1 mg) and $Pd_2(dba)_3$ (0.011 mmol, 5.3 mg, 5.0 mol%) were added and dissolved in anhydrous toluene (1 mL). The head space of the vial was purged with argon then the vial sealed with a screw on cap. The purple solution was gently swirled until the $Pd_2(dba)_3$ dissolved and the color changed to bright yellow (approx. 10 min). In a separate vial equipped with a stir bar, 2,6-diethynylaniline (303 mg, 2.34 mmol, 10 equiv.) and aryl-iodide **5** (1 equiv., 109 mg, 0.23 mmol) were dissolved in anhydrous toluene (1 mL) and DIPEA (12 equiv., 2.76 mmol, 480 uL). While stirring under a stream of argon the catalyst solution was added dropwise the solution containing the aldehyde and alkyne. The vessel was sealed with a screw on cap and heated to 40 °C. After stirring overnight, the reaction was cooled to rt transferred to a round bottom flask and diluted with DCM (approx. 50 mL). Silica gel was added to vessel and crude reaction mixture concentrated onto the silica gel. Silica gel flash chromatography (hexane with a 70-100% gradient of EtOAC over 5 minutes, then held at 100% EtOAc for 20 mim) gave pure **7** (87 mg, 0.18 mmol, 79%) as a white solid. ^1H NMR (400 MHz, $CDCl_3$) δ 8.12 (s, 1H), 8.10 (s, 1H), 7.79 (s, 1H), 7.13 (t, *J* = 7.8 Hz, 1H), 7.06 (s, 1H), 6.93 (d, *J* = 7.5 Hz, 1H), 6.84 (s, 1H), 6.80 (s, 1H), 6.77 (s, 1H), 6.67 (d, *J* = 8.2 Hz, 1H), 4.53 – 4.47 (m, 2H), 3.87 – 3.81 (m, 2H), 3.78 (s, 2H), 3.75 (br, 2N*H*), 3.72 – 3.68 (br, 2N*H*), 3.60 – 3.55 (m, 2H), 3.39 (s, 3H), 3.05 (s, 1H). ^{13}C NMR (100 MHz, $CDCl_3$) δ 165.3, 146.49, 146.48, 138.4, 132.4, 132.2, 130.8, 129.4, 125.6, 124.3, 123.9, 123.6, 123.3, 123.2, 122.1, 118.8, 118.4, 117.9, 115.8, 91.3, 90.2, 87.6, 87.0, 83.1, 77.3, 71.9, 70.6, 69.2, 64.6, 59.2. HRMS (ESI): m/z calcd for $C_{30}H_{26}N_2O_4+H^+$ 479.1965 $[M+H]^+$; found 479.1946.

ss-**AAA.**

To a 20 mL scintillation vial $AsPh_3$ (0.026 mmol, 7.9 mg) and $Pd_2(dba)_3$ (0.0033 mmol, 3.0 mg, 10 mol%) were added and dissolved in anhydrous toluene (0.5 mL). The head space of the vial was purged with argon then the vial sealed with a screw on cap. The purple solution was gently swirled until the $Pd_2(dba)_3$ dissolved and the color changed to bright yellow (approx. 10 min). In a separate vial equipped with a stir bar, 2,6-diethynylaniline (9.2 mg, 0.065 mmol, 1 equiv.) and aryl-iodide **5** (2 equiv., 60 mg, 0.13 mmol) were dissolved in anhydrous toluene (1.3 mL) and DIPEA (12 equiv., 1.6 mmol, 271 uL). While stirring under a stream of argon the catalyst solution was added dropwise to the solution containing 2,6-diethynylaniline and **5**. The vessel was sealed with a screw on cap and heated to 40 °C. After stirring overnight, the reaction was cooled to rt, transferred to a round bottom flask, and diluted with DCM (approx. 50 mL) and TEA (approx. 1 mL). Silica gel (approx. 2 g) was added to the vessel and the crude reaction mixture was concentrated onto the silica gel. Silica gel flash chromatography (hexane with a 70-100% gradient of EtOAC over 5 minutes, then held at 100% EtOAc for 25 min, both organic solvents were basified with 0.1 % TEA) gave *ss*-**AAA** (34 mg, 0.042 mmol, 65%) as a white solid after concentration in vacuo. ^1H NMR (400 MHz, $CDCl_3$) δ 8.15 (t, *J* = 1.6 Hz, 2H), 8.13 (t, *J* = 1.6 Hz, 2H), 7.83 (t, *J* = 1.6 Hz, 2H), 7.18 – 7.12 (overlap, 3H), 6.95 (ddd, *J* = 7.6, 1.5, 1.0 Hz, 2H), 6.87 (ddd, *J* = 2.4, 1.5, 0.5 Hz, 2H), 6.85 (d, *J* = 1.4 Hz, 2H), 6.69 (ddd, *J* = 8.1, 2.4, 1.0 Hz, 2H), 4.54 – 4.49 (m, 4H), 3.89 – 3.84 (m, 4H), 3.79 (br, 2N*H*), 3.74 – 3.70

(overlap, 8H), 3.62 – 3.56 (m, 4H), 3.40 (s, 6H). ^{13}C NMR (100 MHz, $CDCl_3$) δ 165.4 (2C), 146.6, 146.5 (2C), 138.5 (2C), 132.5 (2C), 132.3 (2C), 130.9 (2C), 129.5 (2C), 125.5, 124.4 (2C), 124.0 (2C), 123.9 (2C), 123.4 (2C), 122.3 (2C), 118.4 (2C), 118.0 (2C), 115.9 (2C), 91.4 (2C), 90.3 (2C), 87.8 (2C), 87.1 (2C), 72.1 (2C), 70.7 (2C), 69.3 (2C), 64.6 (2C), 59.3 (2C). HRMS (ESI): m/z calcd for $C_{50}H_{45}N_3O_8$+Na^+: 838.3099 $[M+Na]^+$; found 838.3100.

ss-**BBB**.

To a 20 mL scintillation vial $AsPh_3$ (0.19 mmol, 19 mg) and $Pd_2(dba)_3$ (0.0075 mmol, 6.8 mg, 5 mol%) were added and dissolved in anhydrous toluene (1.0 mL). The head space of the vial was purged with argon then the vial sealed with a screw on cap. The purple solution was gently swirled until the $Pd_2(dba)_3$ dissolved and the color changed to bright yellow (approx. 10 min). In a separate vial equipped with a stir bar, 2,6-diethynylbenzaldehyde (19 mg, 0.12 mmol, 1.0 equiv.) and aryl-iodide **3** (2.5 equiv., 143 mg, 0.3 mmol) were dissolved in anhydrous toluene (3.0 mL) and DIPEA (12 equiv., 1.5 mmol, 261 uL). While stirring under a stream of argon the catalyst solution was added dropwise to the solution containing 2,6-diethynylbenzaldehyde and **3**. The vessel was sealed with a screw on cap and stirred at rt. After stirring overnight, the crude reaction mixture was transferred to a round bottom flask and diluted with DCM (approx. 50 mL) and TEA (approx. 1 mL). Silica gel (approx. 5 g) was added to the vessel and the crude reaction mixture was concentrated onto the silica gel. Silica gel flash chromatography (hexane with a 20-90% gradient of EtOAC) gave pure *ss*-**BBB** (98 mg, 0.11 mmol, 94%) as a white solid after concentration in vacuo. ^{1}H NMR (400 MHz, $CDCl_3$) δ 10.044 (s, 1H), 10.039 (s, 2H), 8.24 – 8.19 (overlap, 4H), 8.06 (t, *J* = 1.6 Hz, 2H), 8.03 (d, *J* = 1.6 Hz, 2H), 7.96 (t, *J* = 1.6 Hz, 1H), 7.92 – 7.87 (overlap, 4H), 7.80 (dt, *J* = 7.7, 1.5 Hz, 2H), 7.56 (t, *J* = 7.7 Hz, 2H), 4.57 – 4.51 (m, 4H), 3.90 – 3.85 (m, 4H), 3.74 – 3.69 (m, 4H), 3.62 – 3.56 (m, 4H), 3.40 (s, 6H). ^{13}C NMR (100 MHz, $CDCl_3$) δ 191.5 (2C), 190.6, 165.1 (2C), 139.7, 138.5 (2C), 137.3 (2C), 136.9, 136.7 (2C), 133.1 (2C), 133.0 (2C), 132.9 (2C), 132.5 (2C), 131.2 (2C), 129.6 (2C), 129.4 (2C), 124.5 (2C), 123.89 (2C), 123.86 (2C), 123.5 (2C), 89.8 (2C), 89.7 (2C), 88.9 (2C), 88.7 (2C), 72.1 (2C), 70.7 (2C), 69.3 (2C), 64.7 (2C), 59.2 (2C). HRMS (ESI): m/z calcd for $C_{53}H_{42}O_{11}$+Na^+: 877.2619 $[M+Na]^+$; found 877.2643.

ss-**ABA**.

To a 20 mL scintillation vial $AsPh_3$ (0.02 mmol, 6.2 mg) and $Pd_2(dba)_3$ (0.0025 mmol, 2.2 mg, 10 mol%) were added and dissolved in anhydrous toluene (0.5 mL). The head space of the vial was purged with argon then the vial sealed with a screw on cap. The purple solution was gently swirled until the $Pd_2(dba)_3$ dissolved and the color changed to bright yellow (approx. 10 min). In a separate vial equipped with a stir bar, 2,6-diethynylbenzaldehyde (7.7 mg, 0.05 mmol, 1 equiv.) and aryl-iodide **5** (2 equiv., 47 mg, 0.1 mmol) were dissolved in anhydrous toluene (1 mL) and DIPEA (12 equiv., 0.6 mmol, 104 uL). While stirring under a stream of argon the catalyst solution was added dropwise to the solution containing 2,6-diethynylbenzaldehyde and **5**. The vessel was

sealed with a screw on cap and heated to 40 °C. After stirring at overnight, the reaction was cooled to rt, transferred to a round bottom flask, and diluted with DCM (approx. 50 mL) and TEA (approx. 1 mL). Silica gel (approx. 2 g) was added to the vessel and the crude reaction mixture was concentrated onto the silica gel. Silica gel flash chromatography (hexane with a 70-100% gradient of EtOAC over 5 minutes, then held at 100% EtOAc for 10 min, both organic solvents were basified with 0.1 % TEA) gave *ss*-**ABA** (31 mg, 0.037 mmol, 74%) as a white solid after concentration in vacuo. ^{1}H NMR (400 MHz, $CDCl_3$) δ 10.04 (s, 1H), 8.18 (t, *J* = 1.6 Hz, 2H), 8.17 (t, *J* = 1.6 Hz, 2H), 8.01 (d, *J* = 1.6 Hz, 2H), 7.95 (t, *J* = 1.6 Hz, 1H), 7.86 (t, *J* = 1.6 Hz, 2H), 7.16 (t, *J* = 7.8 Hz, 2H), 6.96 (dt, *J* = 7.6, 1.2 Hz, 2H), 6.87 (dd, *J* = 2.4, 1.5 Hz, 2H), 6.70 (ddd, *J* = 8.0, 2.5, 1.0 Hz, 2H), 4.55 – 4.50 (m, 4H), 3.90 – 3.85 (m, 4H), 3.77 – 3.68 (overlap, 8H), 3.62 – 3.57 (m, 4H), 3.40 (s, 6H). ^{13}C NMR (100 MHz, $CDCl_3$) δ 190.7, 165.3 (2C), 146.5 (2C), 139.8, 138.5 (2C), 136.9, 133.0 (2C), 132.5 (2C), 132.4 (2C), 131.1 (2C), 129.6 (2C), 124.64 (2C), 124.59 (2C), 123.4 (2C), 123.3 (2C), 122.3 (2C), 118.0 (2C), 116.0 (2C), 91.6 (2C), 90.0 (2C), 88.5 (2C), 86.9 (2C), 72.1 (2C), 70.7 (2C), 69.3 (2C), 64.7 (2C), 59.3 (2C). HRMS (ESI): m/z calcd for $C_{51}H_{44}N_2O_9$+Na^+: 851.2939 $[M+Na]^+$; found 851.2937.

ss-**BAB**.

To a 20 mL scintillation vial $AsPh_3$ (0.017 mmol, 5.1 mg) and $Pd_2(dba)_3$ (0.0021 mmol, 1.9 mg, 10 mol%) were added and dissolved in anhydrous toluene (0.5 mL). The head space of the vial was purged with argon then the vial sealed with a screw on cap. The purple solution was gently swirled until the $Pd_2(dba)_3$ dissolved and the color changed to bright yellow (approx. 10 min). In a separate vial equipped with a stir bar, 2,6-diethynylaniline (5.6 mg, 0.042 mmol, 1 equiv.) and aryl-iodide **3** (2 equiv., 40.0 mg, 0.083 mmol) were dissolved in anhydrous toluene (700 uL) and DIPEA (12 equiv., 0.50 mmol, 82 uL). While stirring under a stream of argon, the catalyst solution was added dropwise dropwise to the solution containing 2,6-diethynylaniline and **3**. The vessel was sealed with a screw on cap and heated to 40 °C. After stirring at overnight, the reaction was cooled to rt, transferred to a round bottom flask, and diluted with DCM (approx. 50 mL) and TEA (approx. 1 mL). Silica gel (approx. 2 g) was added to the vessel and the crude reaction was mixture concentrated onto the silica gel. Silica gel flash chromatography (hexane with a 20-100% gradient of EtOAC over 10 minutes, then held at 100% EtOAc for 10 min, both organic solvents basified with 0.1 % TEA) gave *ss*-**BAB** (31 mg, 0.037 mmol, 87%) as a white solid after concentration in vacuo. ^{1}H NMR (400 MHz, $CDCl_3$) δ 10.03 (s, 2H), 8.18 – 8.15 (overlap, 4H), 8.04 (t, *J* = 1.6 Hz, 2H), 7.88 (dt, *J* = 7.8, 1.4 Hz, 2H), 7.85 (t, *J* = 1.6 Hz, 2H), 7.79 (dt, *J* = 7.7, 1.4 Hz, 2H), 7.55 (t, *J* = 7.7 Hz, 2H), 7.14 (t, *J* = 1.3 Hz, 1H), 6.85 (d, *J* = 1.4 Hz, 2H), 4.55 – 4.51 (m, 4H), 3.89 – 3.85 (m, 4H), 3.83 (s, 2H), 3.74 – 3.69 (m, 4H), 3.61 – 3.57 (m, 4H), 3.40 (s, 6H). ^{13}C NMR (100 MHz, $CDCl_3$) δ 191.5 (2C), 165.3 (2C), 146.7, 138.5 (2C), 137.3 (2C), 136.7 (2C), 133.2 (2C), 132.8 (2C), 132.5 (2C), 131.1 (2C), 129.5 (2C), 129.4 (2C), 125.5, 124.2 (2C), 124.0 (2C), 123.8 (2C), 123.7 (2C), 118.4 (2C), 90.6 (2C), 89.4 (2C), 89.2 (2C), 87.6 (2C), 72.1 (2C), 70.7 (2C), 69.3 (2C), 64.7 (2C), 59.3 (2C). HRMS (ESI): m/z calcd for $C_{52}H_{43}NO_{10}$+Na^+: 864.2779 $[M+Na]^+$; found 864.2791.

ss-**AAB.**

To a 20 mL scintillation vial $AsPh_3$ (0.02 mmol, 6.2 mg) and $Pd_2(dba)_3$ (0.0025 mmol, 2.2 mg, 10 mol%) were added and dissolved in anhydrous toluene (0.5 mL). The head space of the vial was purged with argon then the vial sealed with a screw on cap. The purple solution was gently swirled until the $Pd_2(dba)_3$ dissolved and the color changed to bright yellow (approx. 10 min). In a separate vial equipped with a stir bar, aryl-yne **7** (57 mg, 0.12 mmol, 1.2 equiv.) and aryl-iodide **3** (1.0 equiv., 48 mg, 0.1 mmol) were dissolved in anhydrous toluene (0.5 mL) and DIPEA (24 equiv., 1.2 mmol, 200 uL). While stirring under a stream of argon the Pd catalyst solution was added dropwise to the solution containing **7** and **3**. The vessel was sealed with a screw on cap and heated to 40 °C. After stirring overnight, the reaction was cooled to rt, transferred to a round bottom flask, and diluted with DCM (approx. 50 mL) and TEA (approx. 1 mL). Silica gel (approx. 2 g) was added to the vessel and the crude reaction mixture was concentrated onto the silica gel. Silica gel flash chromatography (hexane with a 70-100% gradient of EtOAC over 5 minutes, then held at 100% EtOAc for 20 min, both organic solvents were basified with 0.1 % TEA) gave *ss*-**AAB** (74 mg, 0.09 mmol, 88%) as a white solid after concentration in vacuo. ^{1}H NMR (400 MHz, $CDCl_3$) δ 10.03 (s, 1H), 8.18 – 8.16 (overlap, 2H), 8.14 (t, *J* = 1.6 Hz, 1H), 8.12 (t, *J* = 1.6 Hz, 1H), 8.04 (t, *J* = 1.6 Hz, 1H), 7.88 (dt, *J* = 7.8, 1.4 Hz, 1H), 7.85 (t, *J* = 1.6 Hz, 1H), 7.82 (t, *J* = 1.6 Hz, 1H), 7.79 (dt, *J* = 7.7, 1.4 Hz, 1H), 7.55 (t, *J* = 7.7 Hz, 1H), 7.17 – 7.12 (overlap, 2H), 6.94 (dt, *J* = 7.6, 1.2 Hz, 1H), 6.87 – 6.83 (overlap, 3H), 6.68 (ddd, *J* = 8.0, 2.5, 1.0 Hz, 1H), 4.55 – 4.49 (overlap, 4H), 3.89 – 3.84 (overlap, 4H), 3.82 (br, 2N*H*), 3.75 – 3.68 (overlap, 6H), 3.61 – 3.56 (overlap, 4H), 3.40 (s, 3H), 3.40 (s, 3H). ^{13}C NMR (100 MHz, $CDCl_3$) δ 191.5, 165.4, 165.3, 146.6, 146.5, 138.50, 138.46, 137.3, 136.7, 133.2, 132.8, 132.52, 132.48, 132.3, 131.1, 130.9, 129.5, 129.44, 129.36, 125.5, 124.4, 124.2, 124.02, 124.01, 123.9, 123.8, 123.7, 123.4, 122.3, 118.4, 118.3, 117.9, 115.9, 91.4, 90.6, 90.3, 89.4, 89.2, 87.8, 87.6, 87.1, 72.1 (2C), 70.7 (2C), 69.3 (2C), 64.7 (2C), 64.6, 59.3 (2C). HRMS (ESI): m/z calcd for $C_{51}H_{44}N_2O_9+Na^+$: 851.2939 $[M+Na]^+$; found 851.2937.

ss-**ABB**.

To a 20 mL scintillation vial $AsPh_3$ (0.011 mmol, 3.3 mg) and $Pd_2(dba)_3$ (0.0013 mmol, 1.3 mg, 10 mol%) were added and dissolved in anhydrous toluene (0.4 mL). The head space of the vial was purged with argon then the vial sealed with a screw on cap. The purple solution was gently swirled until the $Pd_2(dba)_3$ dissolved and the color changed to bright yellow (approx. 10 min). In a separate vial equipped with a stir bar, aryl-yne **6** (28 mg, 0.055 mmol, 1.0 equiv.) and aryl-iodide **5** (1.1 equiv., 26 mg, 0.061 mmol) were dissolved in anhydrous toluene (0.55 mL) and DIPEA (6 equiv., 0.33 mmol, 59 uL). While stirring under a stream of argon the catalyst solution was added dropwise to the solution containing **6** and **5**. The vessel was sealed with a screw on cap and heated to 40 °C. After stirring overnight, the reaction was cooled to rt, transferred to a round bottom flask, and diluted with DCM (approx. 50 mL) and TEA (approx. 1 mL). Silica gel (approx. 2 g) was added to the vessel and the crude reaction mixture was concentrated onto the silica gel. Silica gel flash chromatography (hexane with a 70-100% gradient of EtOAC over 5 minutes, then

held at 100% EtOAc for 10 min, both organic solvents basified with 0.1 % TEA) gave *ss*-**ABB** (37 mg, 0.043 mmol, 79%) as a white solid after concentration in vacuo. ^{1}H NMR (400 MHz, $CDCl_3$) δ 10.04 (s, 2H), 8.23 – 8.21 (overlap, 2H), 8.19 (t, *J* = 1.6 Hz, 1H), 8.17 (t, *J* = 1.6 Hz, 1H), 8.06 (td, *J* = 1.7, 0.6 Hz, 1H), 8.02 (d, *J* = 1.6 Hz, 2H), 7.96 (t, *J* = 1.6 Hz, 1H), 7.91 – 7.88 (overlap, 2H), 7.87 (t, *J* = 1.6 Hz, 1H), 7.80 (dt, *J* = 7.7, 1.4 Hz, 1H), 7.57 (t, *J* = 7.7 Hz, 1H), 7.16 (t, *J* = 7.8 Hz, 1H), 6.96 (dt, *J* = 7.6, 1.2 Hz, 1H), 6.87 (dd, *J* = 2.5, 1.4 Hz, 1H), 6.70 (ddd, *J* = 8.1, 2.4, 1.0 Hz, 1H), 4.56 – 4.52 (overlap, 4H), 3.90 – 3.85 (overlap, 4H), 3.74 – 3.70 (overlap, 6H), 3.61 – 3.57 (overlap, 4H), 3.41 (s, 3H), 3.40 (s, 3H). ^{13}C NMR (100 MHz, $CDCl_3$) δ ^{13}C NMR (100 MHz, $CDCl_3$) δ 191.5, 190.7, 165.3, 165.2, 146.5, 139.8, 138.6, 138.5, 137.3, 136.9, 136.7, 133.2, 133.1, 133.0, 132.9, 132.6, 132.43, 132.36, 131.2, 131.1, 129.58, 129.55, 129.4, 124.64, 124.62, 124.5, 123.94, 123.89, 123.5, 123.33, 123.30, 122.3, 118.0, 116.0, 91.7, 90.1, 89.8, 89.7, 89.0, 88.8, 88.5, 86.9, 72.1 (2C), 70.7 (2C), 69.3 (2C), 64.74, 64.68, 59.3 (2C). HRMS (ESI): m/z calcd for $C_{52}H_{43}NO_{10}$+Na^+: 864.2779 $[M+Na]^+$; Found 864.2786.

ds-**ABA**.

A 7.5 mM solution of *ss*-**ABA** (3.7 mg, 4.5 µmol) in dry $CDCl_3$ (600 uL) was prepared in an NMR tube. An initial ^{1}H NMR spectrum of *ss*-**ABA** was acquired and then 10 uL of a solution of 1% TFA in $CDCl_3$ (v/v) was added. The capped NMR tube was immediately inverted several times to mix, and then the solvent evaporated under a stream of Ar. Dry $CDCl_3$ (600 uL) was added to the NMR tube and the yellow residue allowed to dissolve (approx. 5 min). Another ^{1}H NMR spectrum was acquired, and then the acidic solution was quenched by adding TEA (0.5 uL) directly to the NMR tube. Another ^{1}H NMR spectrum was acquired showing complete conversion to *ds*-**ABA.** Using DCM (~4 mL) the solution was transferred to a test tube and the organic layer washed with water (4 mL) 3 times. The cloudy organic layer was then concentrated in vacuo to give *ds*-**ABA** (3.7 mg, 2.3 µmol, 100%) as a white solid. ^{1}H NMR (400 MHz, $CDCl_3$) δ 8.52 (s, 2H), 8.16 – 8.12 (overlap, 6H), 8.10 (t, *J* = 1.6 Hz, 2H), 8.03 (d, *J* = 1.6 Hz, 4H), 7.86 (t, *J* = 1.6 Hz, 2H), 7.83 (t, *J* = 1.6 Hz, 2H), 7.76 (t, *J* = 1.5 Hz, 2H), 7.49 (s, 2H), 7.41 (dt, *J* = 7.3, 1.7 Hz, 2H), 7.37 (t, *J* = 7.4 Hz, 2H), 7.32 (dt, *J* = 7.4, 1.9 Hz, 2H), 7.15 (t, *J* = 7.8 Hz, 2H), 6.96 (dt, *J* = 7.7, 1.1 Hz, 2H), 6.87 (dd, *J* = 2.5, 1.5 Hz, 2H), 6.69 (ddd, *J* = 8.1, 2.4, 1.0 Hz, 2H), 4.56 – 4.48 (overlap, 8H), 3.92 – 3.84 (overlap, 8H), 3.77 – 3.69 (overlap, 12H), 3.63 – 3.57 (overlap, 8H), 3.42 (s, 6H), 3.41 (s, 6H). ^{13}C NMR (100 MHz, $CDCl_3$) δ 165.3 (2C), 165.2 (2C), 157.8 (2C), 150.1 (2C), 146.6 (2C), 139.0 (2C), 138.5 (2C), 136.9 (2C), 136.8 (2C), 132.7 (2C), 132.35 (2C), 132.29 (4C), 132.1 (2C), 131.7 (2C), 130.9 (4C), 130.1 (2C), 129.52 (2C), 129.47 (2C), 125.0 (2C), 124.5 (2C), 124.2 (2C), 124.0 (2C), 123.9 (2C), 123.7 (2C), 123.7 (2C), 123.6 (2C), 123.5 (2C), 122.3 (2C), 122.0 (2C), 118.0 (2C), 115.9 (2C), 91.5 (2C), 90.9 (2C), 89.5 (2C), 89.4 (2C), 89.3 (2C), 89.3 (2C), 88.1 (2C), 87.1 (2C), 72.1 (2C), 72.1 (2C), 70.8 (2C), 70.7 (2C), 69.33 (2C), 69.32 (2C), 64.6 (4C), 59.31 (2C), 59.29 (2C). HRMS (ESI): m/z calcd for $C_{102}H_{84}N_4O_{16}$+Na^+: 1644.5808 $[M+Na]^+$; found 1644.5810.

ds-**BAB.**

A 7.5 mM solution of *ss*-**BAB** (7.6 mg, 9 µmol) in dry $CDCl_3$ (1.2 mL) was prepared in an NMR tube. An initial ^1H NMR spectrum of *ss*-**BAB** was acquired and then 10 uL of a solution of 1% TFA in $CDCl_3$ (v/v) was added. The capped NMR tube was immediately inverted several times to mix, and then the solvent evaporated under a stream of Ar. Dry $CDCl_3$ (1.2mL) was added to the NMR tube and the yellow residue allowed to dissolve (approx. 5 min). Another ^1H NMR spectrum was acquired, and then the acidic solution was quenched by adding TEA (1 uL) directly to the NMR tube. Another ^1H NMR spectrum was acquired showing nearly complete conversion to *ds*-**BAB**. Using DCM (~4 mL) the solution was transferred to a test tube and the organic layer washed with water (4 mL) 3 times. The cloudy organic layer was then concentrated in vacuo to give *ds*-**BAB** (7.3 mg, 4.4 µmol, 98%) as a white solid. ^1H NMR (400 MHz, $CDCl_3$) δ 10.04 (s, 2H), 8.59 (s, 2H), 8.20 (t, *J* = 1.6 Hz, 2H), 8.18 (t, *J* = 1.6 Hz, 2H), 8.17 (t, *J* = 1.6 Hz, 2H), 8.16 – 8.14 (overlap, 4H), 8.05 (t, *J* = 1.7 Hz, 2H), 7.92 – 7.86 (overlap, 8H), 7.79 (dt, *J* = 7.7, 1.4 Hz, 2H), 7.65 (dt, *J* = 7.8, 1.4 Hz, 2H), 7.61 (t, *J* = 1.5 Hz, 2H), 7.56 (t, *J* = 7.7 Hz, 2H), 7.53 – 7.46 (overlap, 6H), 4.57 – 4.51 (overlap, 8H), 3.92 – 3.85 (overlap, 8H), 3.76 – 3.70 (overlap, 8H), 3.63 – 3.58 (overlap, 8H), 3.42 (s, 6H), 3.41 (s, 6H). ^{13}C NMR (100 MHz, $CDCl_3$) δ 191.4 (2C), 165.04 (2C), 164.99 (2C), 159.0 (2C), 150.2 (2C), 138.7 (2C), 138.4 (2C), 137.3 (2C), 136.6 (2C), 136.1 (2C), 134.2 (2C), 133.2 (2C), 132.7 (2C), 132.4 (2C), 132.2 (2C), 132.05 (2C), 132.02 (2C), 132.00 (2C), 130.9 (2C), 130.6 (2C), 129.4 (2C), 129.31 (2C), 129.28 (2C), 129.0 (2C), 128.8 (2C), 125.7 (2C), 124.3 (2C), 124.00 (2C), 123.98 (2C), 123.94 (2C), 123.91 (2C), 123.64 (2C), 123.55 (2C), 123.4 (2C), 90.4 (2C), 90.2 (2C), 89.9 (2C), 89.4 (2C), 89.2 (2C), 88.7 (2C), 88.6 (2C), 88.5 (2C), 72.13 (2C), 72.09 (2C), 70.72 (2C), 70.70 (2C), 69.29 (2C), 69.26 (2C), 64.6 (2C), 64.5 (2C), 59.28 (2C), 59.26 (2C). HRMS (ESI): m/z calcd for $C_{104}H_{82}N_2O_{18}$+Na^+: 1670.5488 $[M+Na]^+$; found 1670.5469.

ds-**AAB**.

A 7.5 mM solution of *ss*-**AAB** (3.7 mg, 4.5 umol) in dry $CDCl_3$ (600 uL) was prepared in an NMR tube. An initial ^1H NMR spectrum of *ss*-**AAB** was acquired and then TFA was added (2 uL). The capped NMR tube was immediately inverted several times to mix. Another ^1H NMR spectrum was acquired, and then the acidic solution was quenched by adding TEA (6 uL) directly to the NMR tube. Another ^1H NMR spectrum was acquired showing nearly complete conversion to *ds*-**AAB.** Using DCM (~4 mL) the solution was transferred to a test tube and the organic layer washed with water (4 mL) 3 times. The cloudy organic layer was then concentrated in vacuo to give *ds*-**AAB** (3.7 mg, 2.3 µmol, 100%) as a white solid. ^1H NMR (400 MHz, $CDCl_3$) δ 8.45 (s, 2H), 8.10 (t, *J* = 1.6 Hz, 2H), 8.08 (t, *J* = 1.6 Hz, 2H), 8.01 (t, *J* = 1.6 Hz, 2H), 8.00 – 7.97 (overlap, 4H), 7.78 – 7.72 (overlap, 6H), 7.50 (dt, *J* = 7.7, 1.4 Hz, 2H), 7.46 (t, *J* = 1.4 Hz, 2H), 7.40 – 7.37 (overlap, 4H), 7.34 (t, *J* = 7.6 Hz, 2H), 7.14 (t, *J* = 7.8 Hz, 2H), 6.94 (dt, *J* = 7.5, 1.2 Hz, 2H), 6.85 (dd, *J* = 2.4, 1.5 Hz, 2H), 6.68 (ddd, *J* = 8.1, 2.4, 1.0 Hz, 2H), 4.53 – 4.45 (overlap, 8H), 3.90 – 3.82 (overlap, 8H), 3.78 – 3.68 (overlap, 12H), 3.63 – 3.57 (overlap, 8H), 3.42 (s, 6H), 3.40 (s, 6H). ^{13}C NMR (100 MHz, $CDCl_3$) δ 165.3 (2C), 165.2 (2C), 159.4

(2C), 150.5 (2C), 146.6 (2C), 139.0 (2C), 138.4 (2C), 136.2 (2C), 134.3 (2C), 132.6 (2C), 132.5 (2C), 132.4 (2C), 132.3 (2C), 132.2 (2C), 132.1 (2C), 130.9 (2C), 130.8 (2C), 129.5 (2C), 129.1 (2C), 129.0 (2C), 125.8 (2C), 124.42 (2C), 124.36 (2C), 124.12 (2C), 124.05 (2C), 124.0 (2C), 123.8 (4C), 123.5 (2C), 123.4 (2C), 122.2 (2C), 118.0 (2C), 115.9 (2C), 91.5 (2C), 90.4 (2C), 89.94 (2C), 89.88 (2C), 88.75 (2C), 88.69 (2C), 88.6 (2C), 87.1 (2C), 72.12 (2C), 72.09 (2C), 70.74 (2C), 70.72 (2C), 69.3 (4C), 64.6 (4C), 59.29 (2C), 59.27 (2C). HRMS (ESI): m/z calcd for $C_{102}H_{84}N_4O_{16}$+Na^+: 1644.5808 $[M+Na]^+$; found 1644.5868.

ds-**ABB.**

A 7.5 mM solution of *ss*-**ABB** (7.6 mg, 9 umol) in dry $CDCl_3$ (1.2 mL) was prepared in an NMR tube. An initial ^{1}H NMR spectrum of *ss*-**ABA** was acquired and then 10 uL of a solution of 1% TFA in $CDCl_3$ (v/v) was added. The capped NMR tube was immediately inverted several times to mix, and then the solvent evaporated under a stream of Ar. Dry $CDCl_3$ (600 uL) was added to the NMR tube, 5 uL of a solution of 1% TFA in $CDCl_3$ (v/v) was added, and the yellow residue allowed to dissolve (approx. 5 min). Another ^{1}H NMR spectrum was acquired, and then the acidic solution was quenched by adding TEA (1 uL) directly to the NMR tube. Another ^{1}H NMR spectrum was acquired showing a mix of *fold*-**ABB** and *ds*-**ABB**, 2:8 respectively. The solution was loaded directly onto a silica gel column for purification. Silica gel flash chromatography (DCM with a 0-50% gradient of THF, both organic solvents basified with 0.1% TEA) gave *ds*-**ABB** (5.0 mg, 3.0 µmol, 66%) as a white solid after concentration in vacuo. ^{1}H NMR (400 MHz, $CDCl_3$) δ 10.04 (s, 2H), 8.59 (s, 2H), 8.23 – 8.19 (overlap, 6H), 8.18 (t, *J* = 1.6 Hz, 2H), 8.13 – 8.09 (overlap, 4H), 8.06 (s, 2H), 7.94 (t, *J* = 1.5 Hz, 2H), 7.92 – 7.87 (overlap, 4H), 7.87 – 7.77 (overlap, 2H), 7.80 (dt, *J* = 7.8, 1.6 Hz, 2H), 7.60 – 7.53 (overlap, 4H), 7.50 – 7.41 (overlap, 4H), 7.37 (dt, *J* = 7.4, 1.6 Hz, 2H), 4.57 – 4.52 (overlap, 8H), 3.91 – 3.86 (overlap, 8H), 3.75 – 3.70 (overlap, 8H), 3.63 – 3.58 (overlap, 8H), 3.42 (s, 6H), 3.41 (s, 6H). ^{13}C NMR (100 MHz, $CDCl_3$) δ 191.5 (2C), 165.2 (2C), 165.1 (2C), 157.8 (2C), 150.2 (2C), 139.0 (2C), 138.5 (2C), 137.3 (2C), 136.9 (2C), 136.77 (2C), 136.75 (2C), 136.7 (2C), 133.2 (2C), 132.81 (2C), 132.75 (2C), 132.41 (2C), 132.38 (2C), 132.1 (2C), 131.6 (2C), 131.1 (2C), 131.0 (2C), 130.1 (2C), 129.51 (2C), 129.46 (2C), 129.4 (2C), 125.0 (2C), 124.2 (2C), 124.02 (2C), 123.96 (4C), 123.9 (2C), 123.8 (2C), 123.6 (2C), 122.1 (2C), 90.9 (2C), 89.7 (2C), 89.6 (2C), 89.4 (2C), 89.3 (2C), 89.2 (2C), 89.1 (2C), 88.1 (2C), 72.14 (2C), 72.12 (2C), 70.8 (4C), 69.33 (2C), 69.31 (2C), 64.7 (2C), 64.6, (2C), 59.3 (4C). HRMS (ESI): m/z calcd for $C_{104}H_{82}N_2O_{18}$+Na^+: 1670.5488 $[M+Na]^+$; found 1670.5450.

fold-**AAB.**

A 3.9 mM solution of *ss*-**AAB** (6.4 mg, 7.7 umol) in dry $CDCl_3$ (2 mL) was prepared in a 100 mL flask, and then a 5 uL of a solution of 1% TFA in $CDCl_3$ (v/v) was added. The solvent was evaporated under a stream of Ar, then the residue was dissolved in 80 mL dry $CDCl_3$ (100 uM **AAB**), and then 5 uL of a solution of 1% TFA in $CDCl_3$ (v/v) was added. The solution was allowed to stand for 5 min and then the acid was quenched with 6 uL TEA. An ^{1}H NMR spectrum of an aliquot of the solution showed complete

consumption of *ss*-**AAB** and a 9:1 mixture of *fold*-**AAB** to *ds*-**AAB**. The solution was concentrated to approx. 400 uL and loaded onto a silica gel column for purification. Silica gel flash chromatography (DCM with a 0-50% gradient of THF, both organic solvents basified with 0.1% TEA) gave *fold*-**ABB** (5.0 mg, 6.2 umol, 79%) as a white solid after concentration in vacuo. ^{1}H NMR (400 MHz, $CDCl_3$) δ 8.74 (s, 1H), 8.34 (t, *J* = 1.7 Hz, 1H), 8.13 – 8.07 (overlap, 6H), 7.87 (dt, *J* = 7.9, 1.3 Hz, 1H), 7.74 (s, 1H), 7.59 (dt, *J* = 7.7, 1.4 Hz, 1H), 7.48 (t, *J* = 7.7 Hz, 1H), 7.44 – 7.37 (overlap, 4H), 6.79 (d, *J* = 1.4 Hz, 2H), 4.56 – 4.50 (overlap, 4H), 3.90 – 3.86 (overlap, 4H), 3.82 (s, 2H), 3.76 – 3.71 (overlap, 4H), 3.63 – 3.59 (overlap, 4H), 3.43 (s, 6H). ^{13}C NMR (100 MHz, $CDCl_3$) δ 165.5, 165.4, 158.7, 150.0, 146.9, 142.0, 141.9, 136.7, 134.5, 132.7, 131.0 (2C), 130.9, 130.8, 130.64, 130.61, 129.5, 129.1, 128.5 (2C), 128.4, 128.1, 124.22, 124.19, 124.18, 124.03, 123.99, 123.9 (2C), 123.6, 121.0, 116.9 (2C), 91.5, 91.2, 91.1 (2C), 89.4, 89.0, 88.41, 88.38, 72.1 (2C), 70.8 (2C), 69.3 (2C), 64.6 (2C), 59.3 (2C). HRMS (ESI): m/z calcd for $C_{51}H_{42}N_2O_8+Na^+$: 833.2833 $[M+Na]^+$; found 833.2827.

fold-**ABB.**

A 7.5 mM solution of *ss*-**ABB** (3.8 mg, 4.5 umol) in dry $CDCl_3$ (600 uL) was prepared in an NMR tube. An initial ^{1}H NMR spectrum of *ss*-**ABB** was acquired and then TFA (2 uL) was added. The capped NMR tube was immediately inverted several times to mix. Another ^{1}H NMR spectrum was acquired, and then the acidic solution was quenched by adding TEA (6 uL) directly to the NMR tube. Another ^{1}H NMR spectrum was acquired showing complete conversion to *fold*-**ABB.** Using DCM (~4 mL) the solution was transferred to a test tube and the organic layer washed with water (4 mL) 3 times. The cloudy organic layer was then concentrated in vacuo to give *fold*-**ABB** (3.7 mg, 4.5 umol, 100%) as a white solid. ^{1}H NMR (400 MHz, $CDCl_3$) δ 10.02 (s, 1H), 8.72 (s, 1H), 8.34 (t, *J* = 1.6 Hz, 1H), 8.16 (t, *J* = 1.6 Hz, 1H), 8.15 – 8.11 (overlap, 5H), 8.10 (t, *J* = 1.6 Hz, 1H), 7.94 (d, *J* = 1.6 Hz, 2H), 7.83 (dt, *J* = 7.7, 1.4 Hz, 1H), 7.73 (s, 1H), 7.58 (dt, *J* = 7.7, 1.4 Hz, 1H), 7.48 (t, *J* = 7.7 Hz, 1H), 7.44 – 7.36 (overlap, 3H), 4.56 – 4.51 (overlap, 4H), 3.91 – 3.86 (m, 4H), 3.76 – 3.71 (overlap, 4H), 3.63 – 3.59 (overlap, 4H), 3.42 (s, 6H). ^{13}C NMR (100 MHz, $CDCl_3$) δ 190.7, 165.24, 165.23, 158.5, 149.9, 142.7, 142.0, 141.9, 137.1, 136.7, 134.2, 132.8, 131.33, 131.30, 131.22, 131.19, 131.1 (3C), 129.6 (2C), 129.2, 128.7, 128.60, 128.57, 124.6 (2C), 124.4, 124.3, 123.9, 123.6, 123.5, 123.4, 120.7, 91.8, 91.4, 90.54, 90.48, 89.27, 89.26, 89.2, 88.9, 72.1 (2C), 70.8 (2C), 69.3 (2C), 64.7 (2C), 59.3 (2C). HRMS (ESI): m/z calcd for $C_{52}H_{41}NO_9+Na^+$: 846.2674 $[M+Na]^+$; found 846.2718.

ds-**AAA·BBB.**

Solutions of **AAA** and **BBB** in $CDCl_3$ were combined in an NMR tube to give equimolar amounts of each trimer, then the solution was diluted with $CDCl_3$ to a final concentration of 0.80 mM trimer and total volume of 600 uL. An initial ^{1}H NMR spectrum was acquired to verify the 1:1 sociometry of **AAA** and **BBB** and then 5 uL of a solution of 1% TFA in $CDCl_3$ (v/v) was added. The capped NMR tube was immediately inverted several times to mix, and then concentrated to dryness under a stream of Ar. The residue

was dissolved in dry $CDCl_3$ (600 uL) and the acidic solution was quenched by adding TEA (6 uL) directly to the NMR tube. DCM (~4 mL) the solution was transferred to a test tube and the organic layer washed with water (4 mL) 3 times. The cloudy organic layer was then concentrated in vacuo to give *ds*-**AAA·BBB** as a white solid. ^1H NMR (400 MHz, $CDCl_3$ with 0.1 % v/v TEA, 50 °C) δ 8.64 (s, 1H), 8.52 (s, 2H), 8.19 – 8.13 (overlap, 8H), 8.13 – 8.07 (overlap, 4H), 7.94 – 7.87 (overlap, 6H), 7.83 (s, 1H), 7.65 (s, 1H), 7.63 (d, *J* = 7.6 Hz, 2H), 7.56 (s, 2H), 7.51 – 7.35 (overlap, 8H), 7.31 (d, *J* = 7.6 Hz, 2H), 4.57 – 4.52 (overlap, 8H), 3.92 – 3.87 (overlap, 8H), 3.77 – 3.73 (overlap, 8H), 3.65 – 3.60 (overlap, 8H), 3.43 (s, 12H). HRMS (MALDI-TOF): m/z calcd for $C_{103}H_{81}N_3O_{16}$+Na^+: 1639.554 $[M+Na]^+$; found 1639.559.

ds-**ABA·BAB**

Solutions of **ABA** and **BAB** (as mixtures of ss-ABO and ds-ABO) in $CDCl_3$ were combined in an NMR tube to give equimolar amounts of each trimer, then the solution was diluted with $CDCl_3$ to a final concentration of 0.40 mM trimer and total volume of 600 uL. An initial ^1H NMR spectrum was acquired to verify the 1:1 sociometry of **ABA** and **BAB** and then 5 uL of a solution of 1% TFA in $CDCl_3$ (v/v) was added. The capped NMR tube was immediately inverted several times to mix, and then concentrated to dryness under a stream of Ar. The residue was dissolved in dry $CDCl_3$ (600 uL) and the acidic solution was quenched by adding TEA (6 uL) directly to the NMR tube. DCM (~4 mL) the solution was transferred to a test tube and the organic layer washed with water (4 mL) 3 times. The cloudy organic layer was then concentrated in vacuo to give *ds*-**ABA·BAB** as a white solid. ^1H NMR (400 MHz, $CDCl_3$ with 0.1 % v/v TEA, 50 °C) δ 8.62 (s, 1H), 8.49 (s, 2H), 8.17 – 8.11 (overlap, 8H), 8.07 (d, *J* = 12.2 Hz, 4H), 7.91 – 7.86 (overlap, 6H), 7.82 (s, 1H), 7.64 – 7.59 (overlap, 3H), 7.55 (s, 2H), 7.51 – 7.35 (overlap, 8H), 7.30 (d, *J* = 7.6 Hz, 2H), 4.57 – 4.51 (overlap, 8H), 3.92 – 3.87 (overlap, 8H), 3.78 – 3.73 (overlap, 8H), 3.65 – 3.60 (overlap, 8H), 3.44 (s, 12H). HRMS (MALDI-TOF): m/z calcd for $C_{103}H_{81}N_3O_{16}$+Na^+: 1639.554 $[M+Na]^+$; found 1639.563.

ds-**AAB·BBA**

Solutions of **AAB** and **BBA** (as mixtures of ss-ABO and ds-ABO) in $CDCl_3$ were combined in an NMR tube to give equimolar amounts of each trimer, then the solution was diluted with $CDCl_3$ to a final concentration of 0.26 mM trimer and total volume of 600 uL. An initial ^1H NMR spectrum was acquired to verify the 1:1 sociometry of **AAB** and **BBA** and then 5 uL of a solution of 1% TFA in $CDCl_3$ (v/v) was added. The capped NMR tube was immediately inverted several times to mix, and then concentrated to dryness under a stream of Ar. The residue was dissolved in dry $CDCl_3$ (600 uL) and the acidic solution was quenched by adding TEA (6 uL) directly to the NMR tube. DCM (~4 mL) the solution was transferred to a test tube and the organic layer washed with water (4 mL) 3 times. The cloudy organic layer was then concentrated in vacuo to give *ds*-**AAB·BBA** as a white solid. ^1H NMR (400 MHz, $CDCl_3$ with 0.01 % v/v TEA, 50 °C) δ 8.65 (s, 1H), 8.53 (s, 2H), 8.20 – 8.14 (overlap, 8H), 8.14 – 8.05 (overlap, 4H), 7.94 – 7.87

(overlap, 6H), 7.84 (s, 1H), 7.68 – 7.61 (overlap, 3H), 7.57 (s, 2H), 7.51 – 7.36 (overlap, 8H), 7.32 (d, *J* = 7.8 Hz, 2H), 4.58 – 4.51 (overlap, 8H), 3.93 – 3.87 (overlap, 8H), 3.78 – 3.73 (overlap, 8H), 3.64 – 3.60 (overlap, 8H), 3.43 (s, 12H). HRMS (MALDI-TOF): m/z calcd for $C_{103}H_{81}N_3O_{16}+Na^+$: 1639.554 $[M+Na]^+$; found 1639.561.

References:

[1] Strom, K. R.; Szostak, J. W.; Prywes, N., Transfer of Sequence Information and Replication of Diimine Duplexes. *J. Org. Chem.* 2019, 84 (7), 3754-3761.

[2] Pollock, J.B.; Cook, T.R.;, P.J., Photophysical and Computational Investigations of Bis(phosphine) Organoplatinum(II) Metallacycles *J. Am. Chem. Soc.* 2012, 134 (25), 10607–10620.

[3] Saf, R.; Swoboda, P.; Hummel, K; Czaputa, R., New derivatives of 4,4,5,5-tetramethyl-4,5-dihydro-1-*H*-imidazol-1-oxyl 3-oxide as monomers for polyradicals with special magnetic properties. *J. Heterocycl. Chem.* 1993, 30, 425-428.

[4] Chen (2022). Multiple curve fitting with common parameters using NLINFIT (https://www.mathworks.com/matlabcentral/fileexchange/40613-multiple-curve-fitting-with-common-parameters-using-nlinfit), MATLAB Central File Exchange. Retrieved June 8, 2022.

^{1}H NMR (400 MHz, $CDCl_3$)

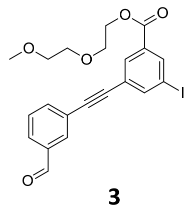

3

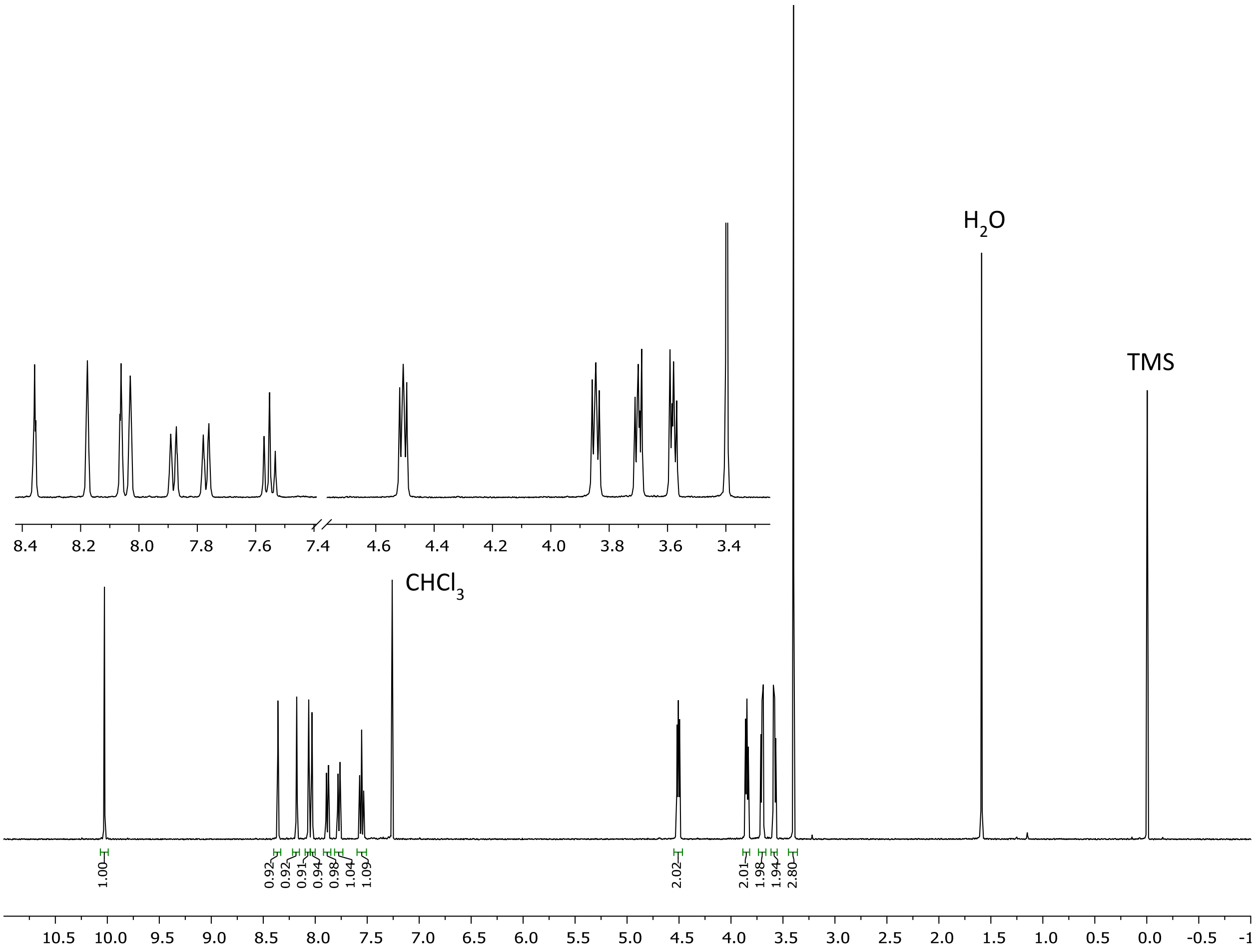

^{13}C NMR (100 MHz, $CDCl_3$)

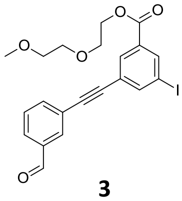

3

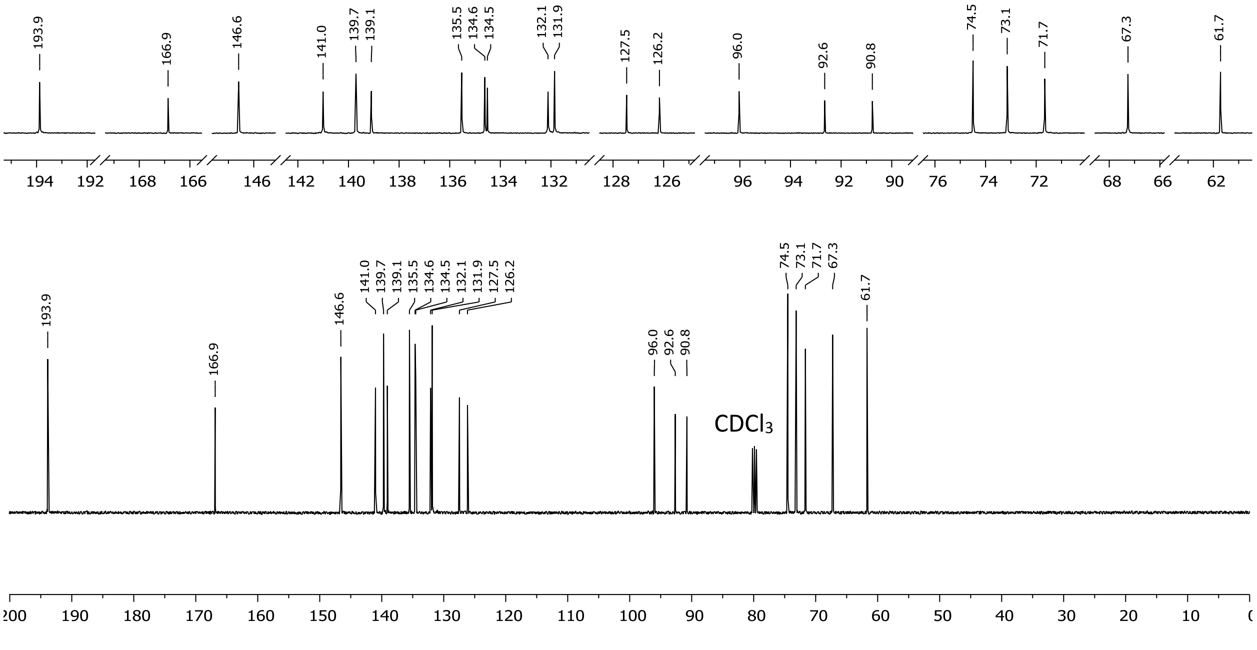

^{1}H NMR (400 MHz, $CDCl_3$)

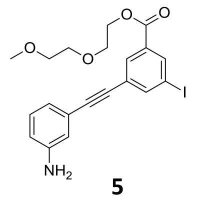

5

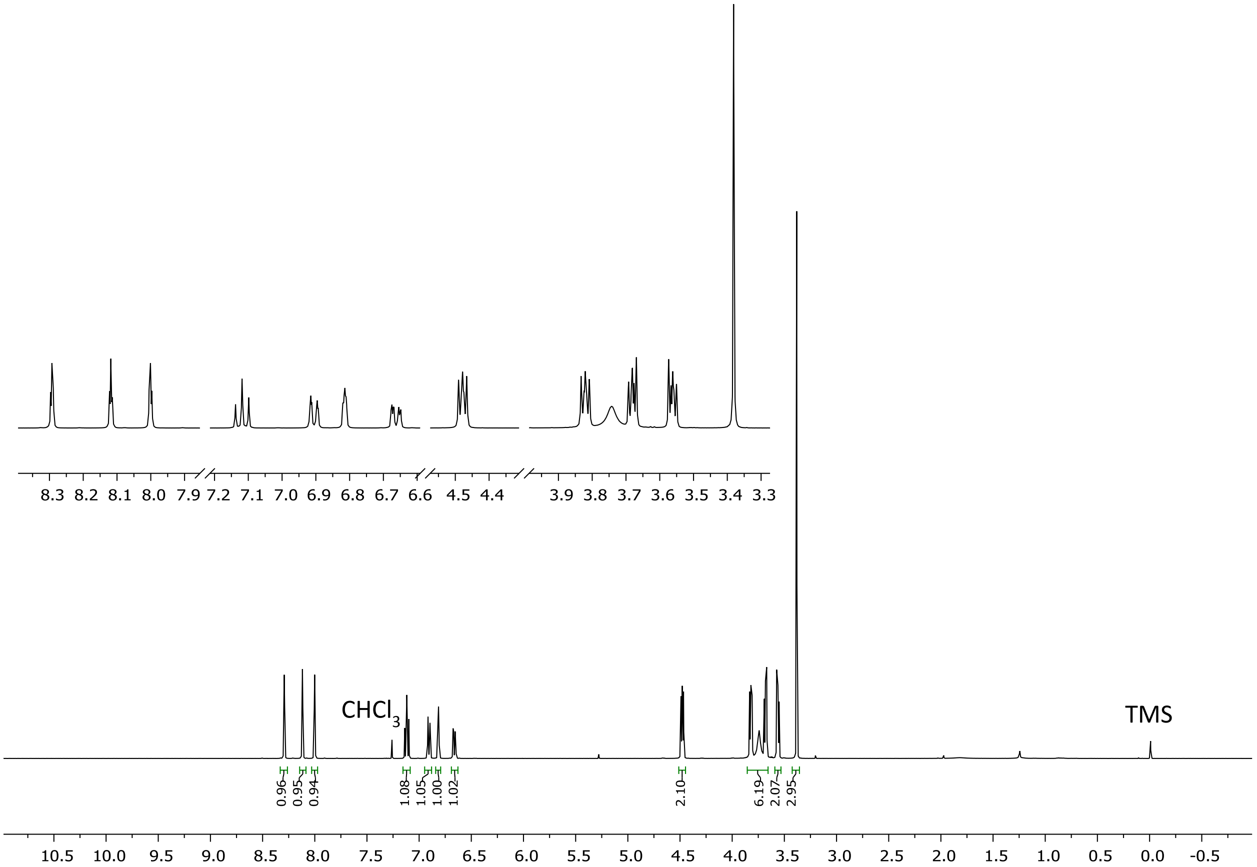

^{13}C NMR (100 MHz, $CDCl_3$)

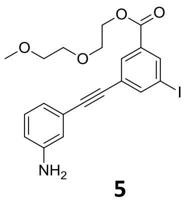

5

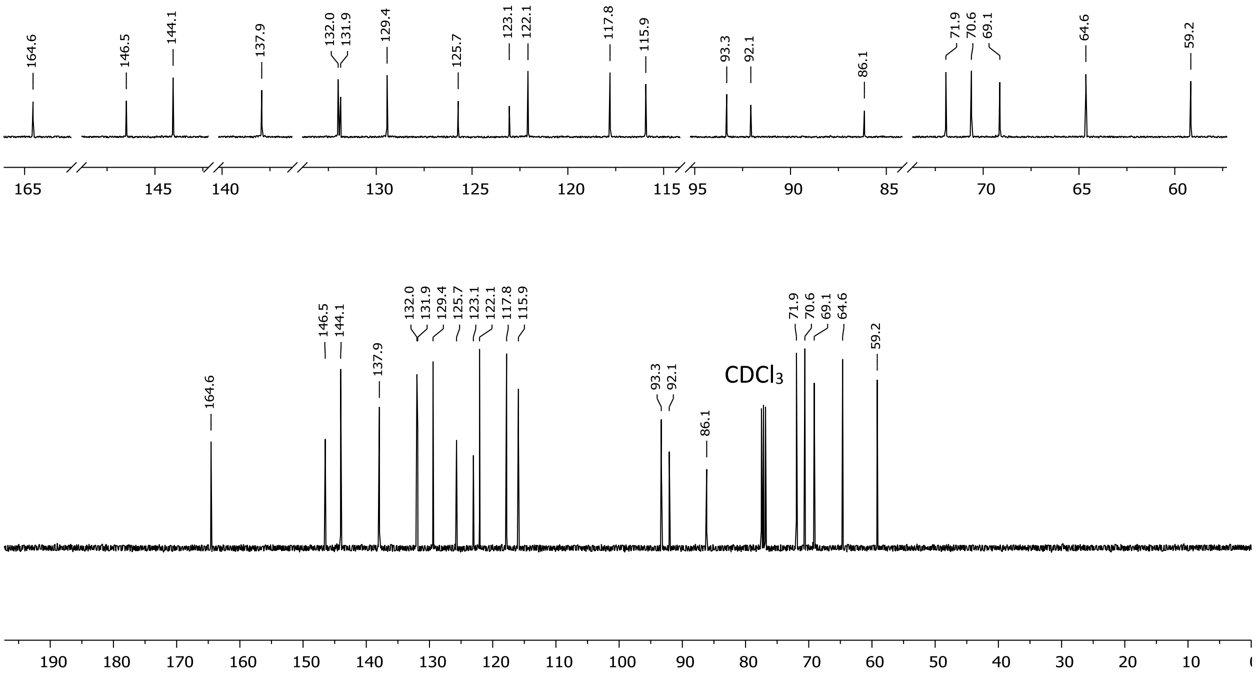

^{1}H NMR (400 MHz, CDCl3)

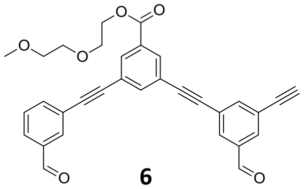

6

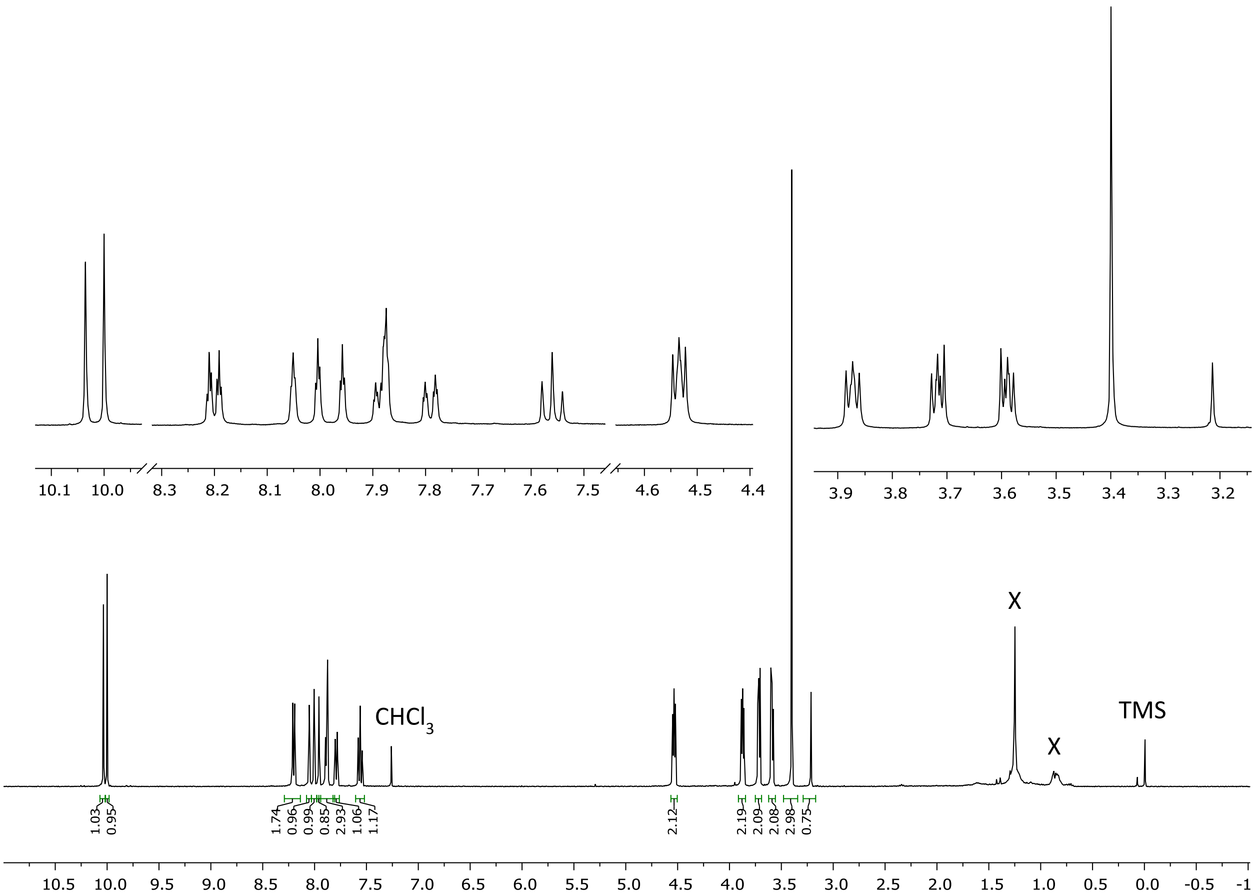

^{13}C NMR (100 MHz, $CDCl_3$)

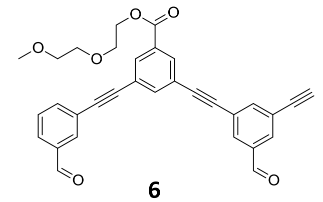

6

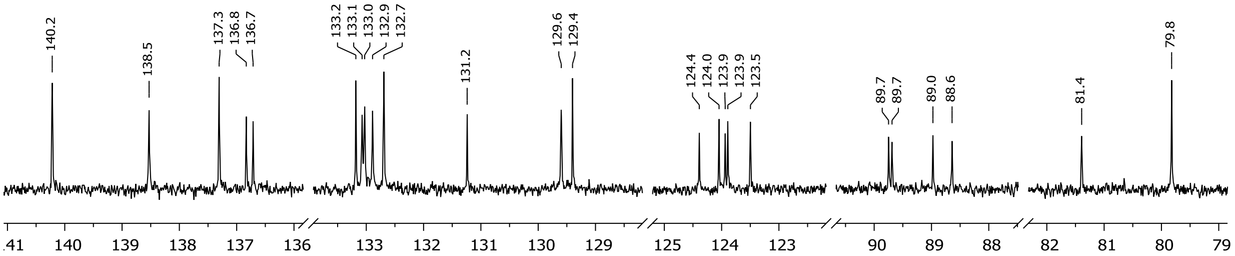

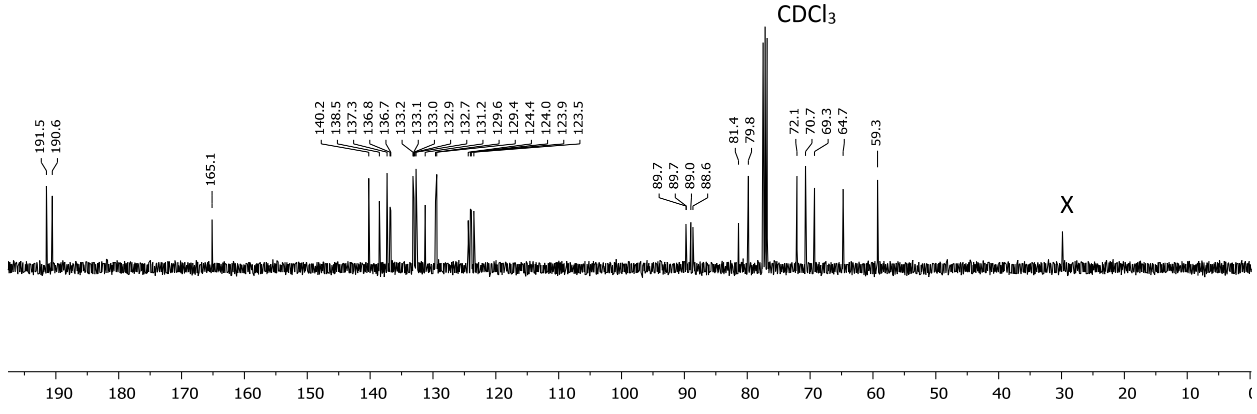

^{1}H NMR (400 MHz, $CDCl_3$)

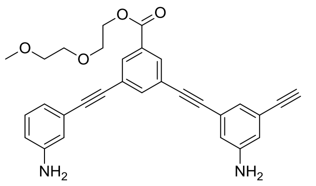

7

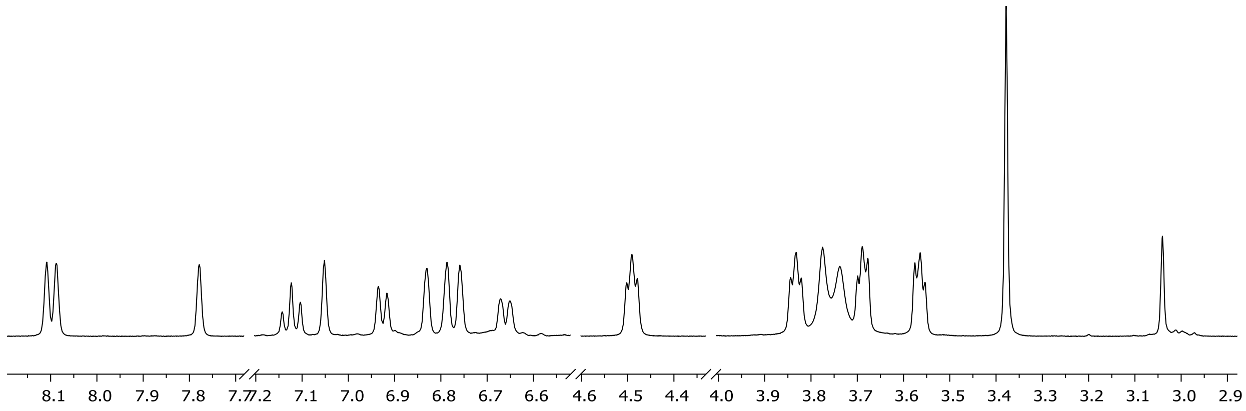

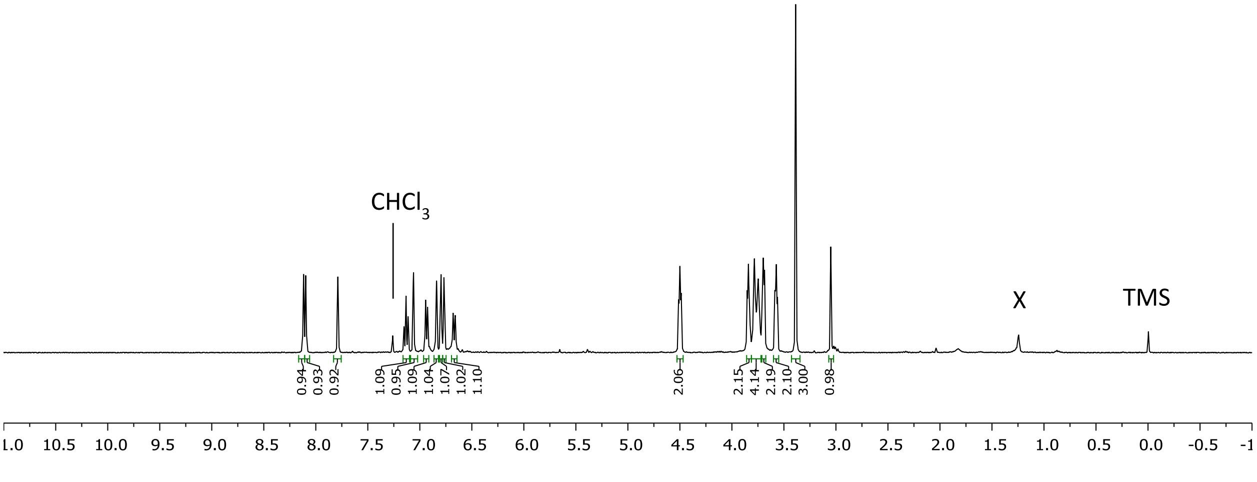

^{13}C NMR (100 MHz, $CDCl_3$)

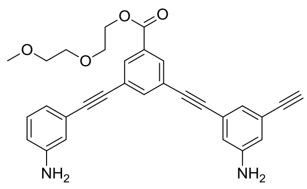

7

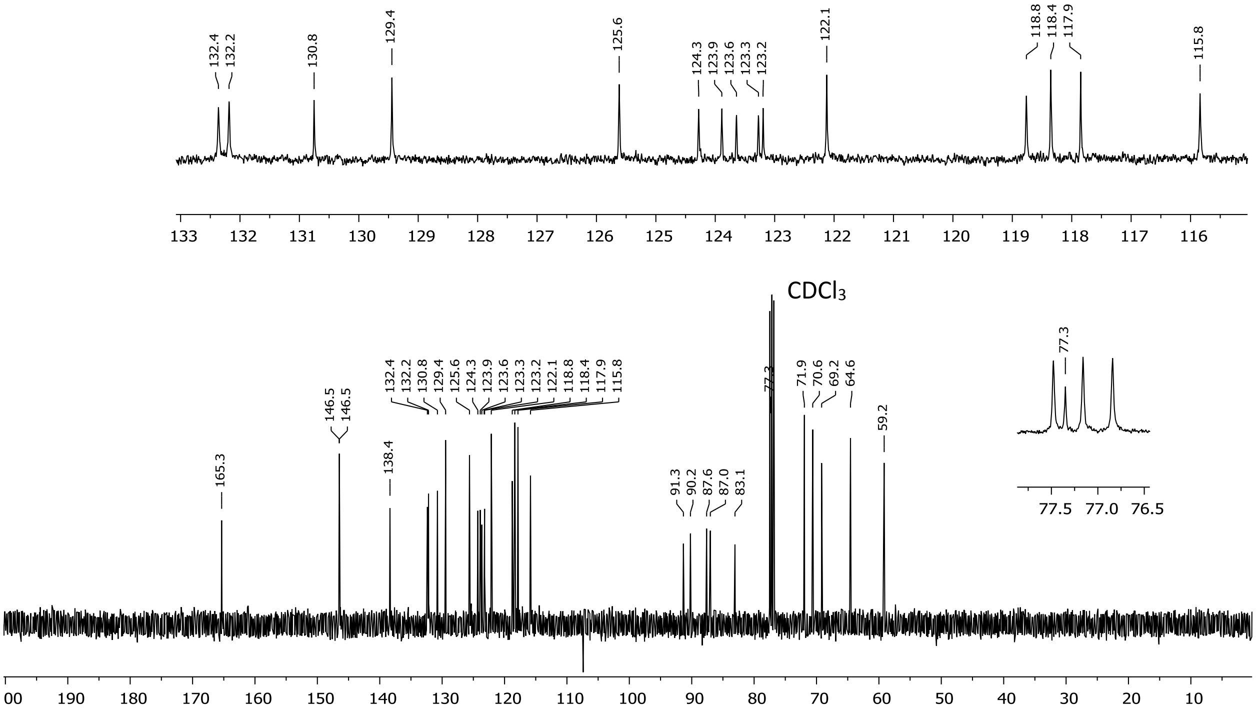

^1H NMR (400 MHz, $CDCl_3$)

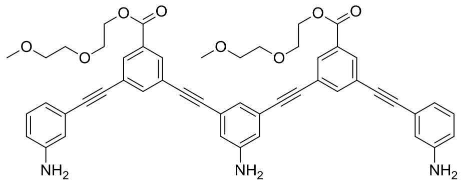

ss-**AAA**

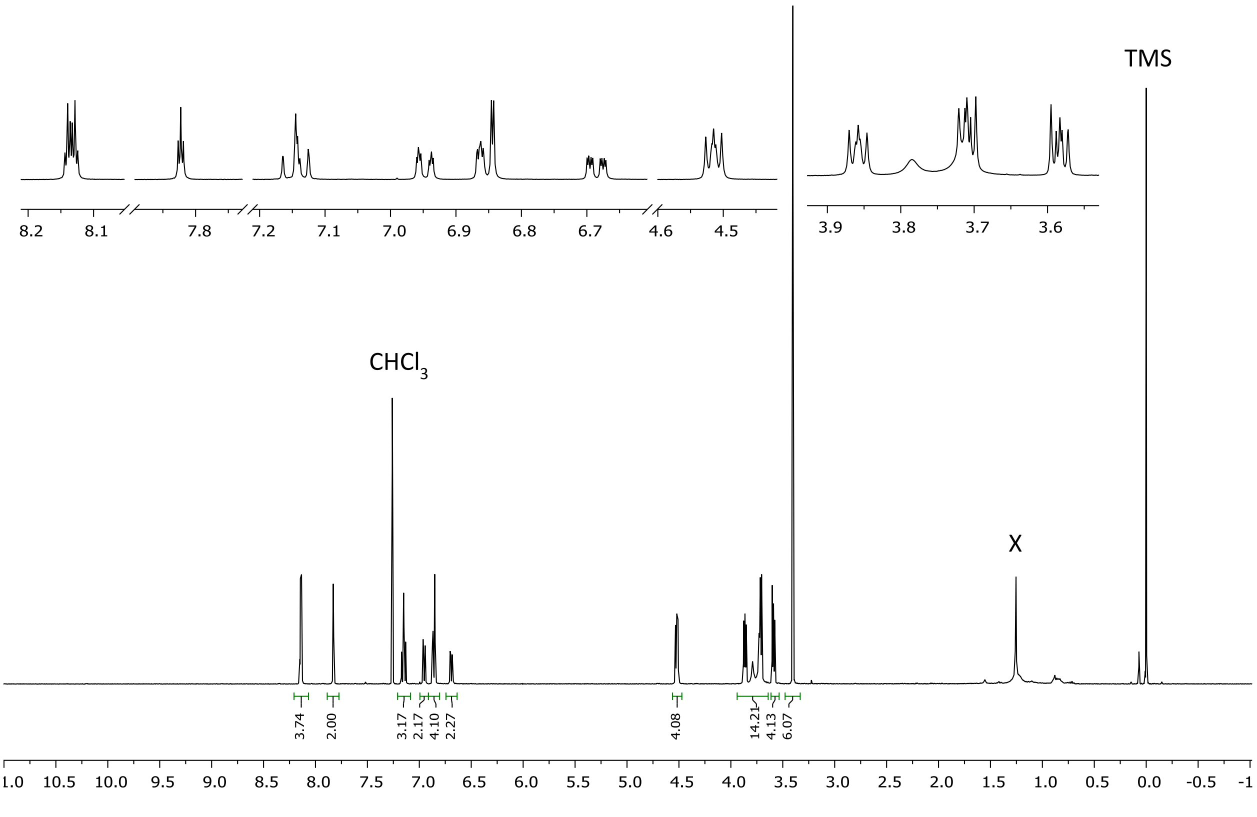

^{13}C NMR (100 MHz, $CDCl_3$)

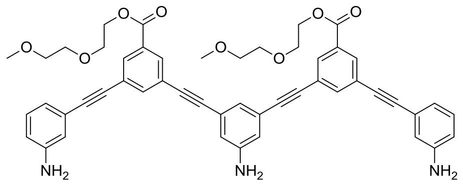

ss-**AAA**

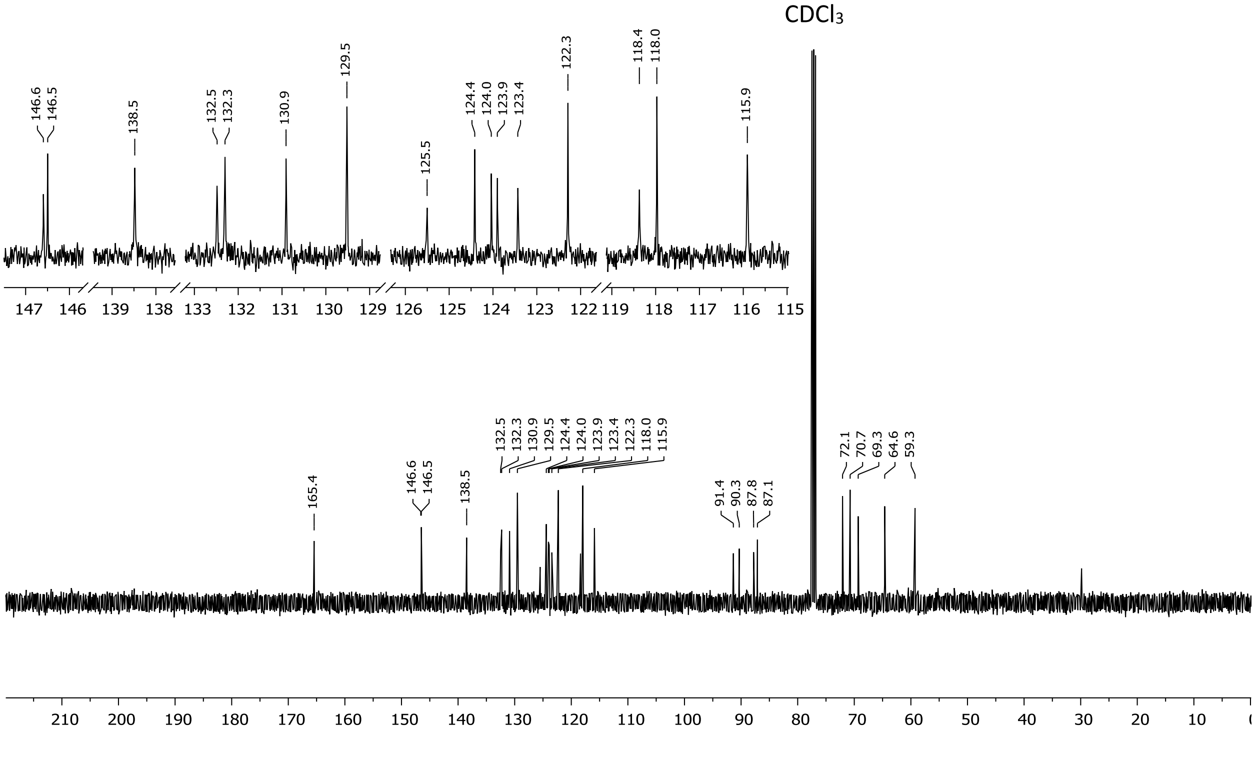

^{1}H NMR (400 MHz, $CDCl_3$)

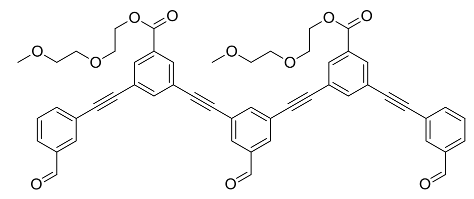

ss-**BBB**

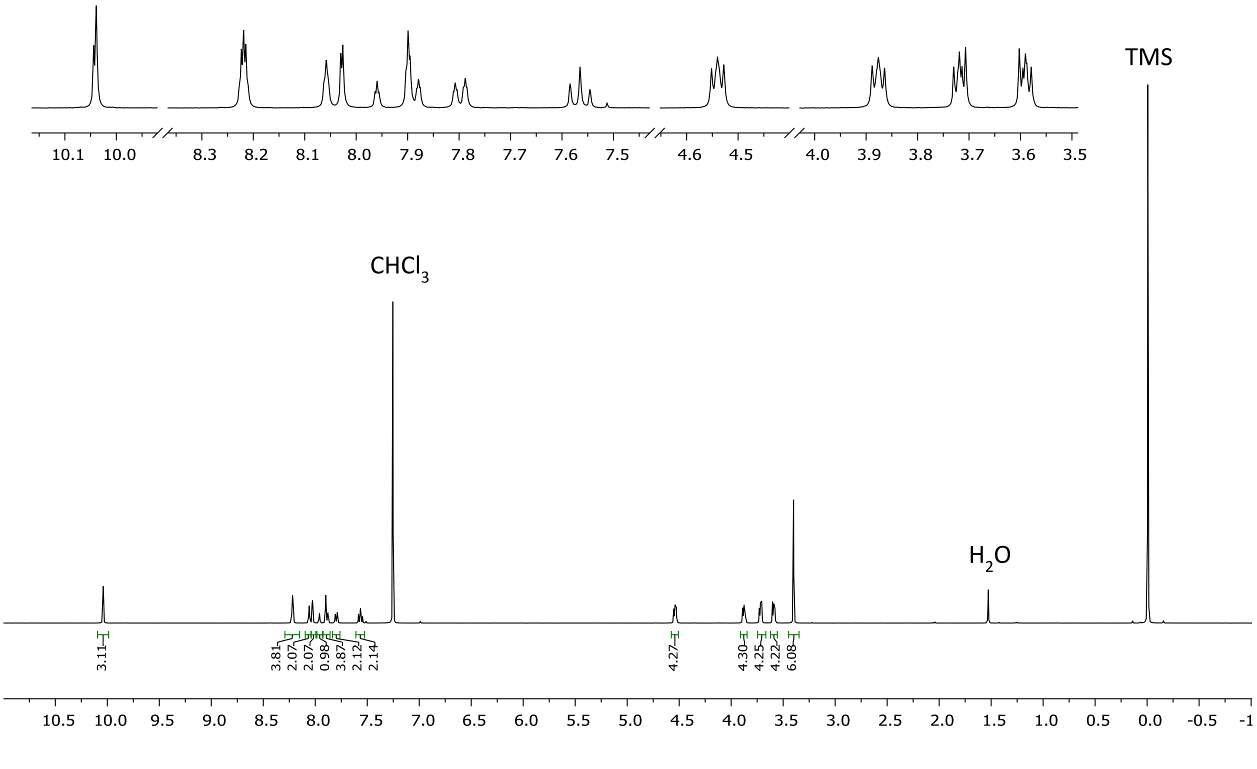

^{13}C NMR (100 MHz, $CDCl_3$)

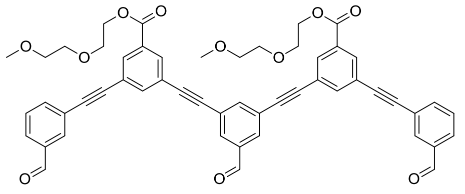

ss-**BBB**

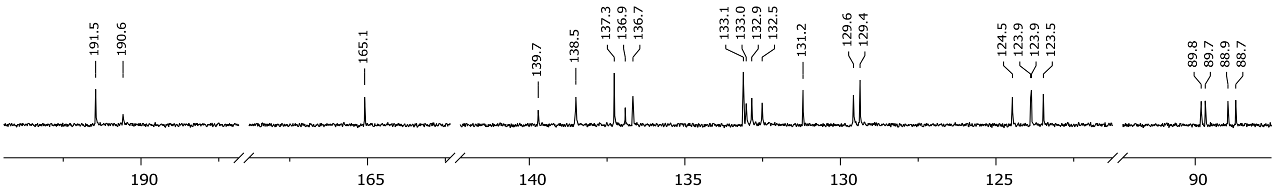

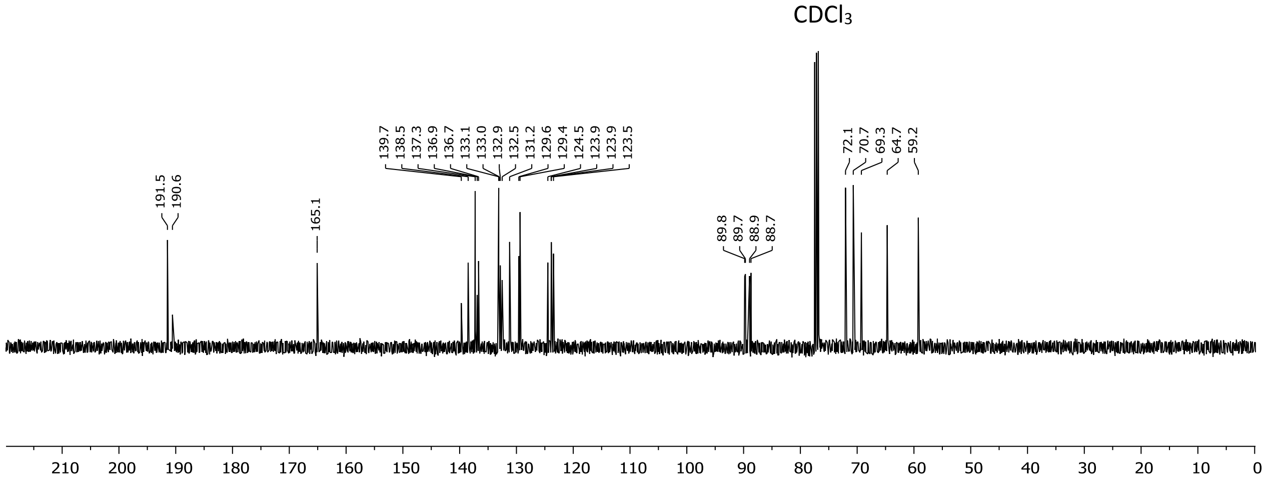

^{1}H NMR (400 MHz, $CDCl_3$ w/ 0.1% TEA)

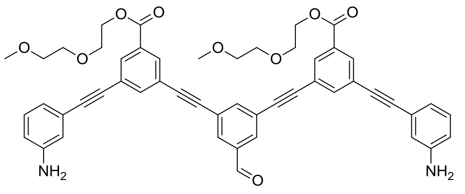

ss-**ABA**

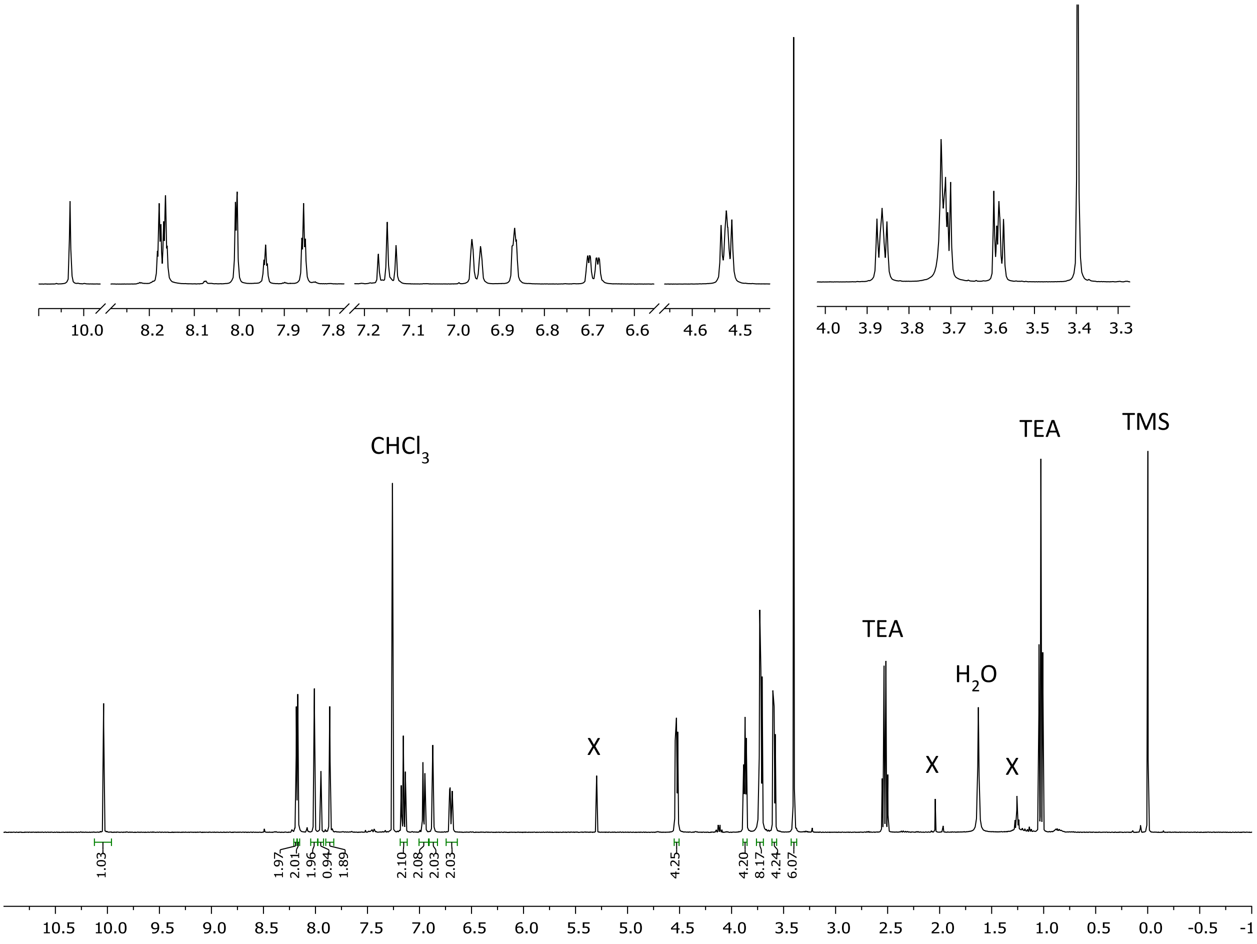

^{13}C NMR (100 MHz, $CDCl_3$ w/ 0.1% TEA)

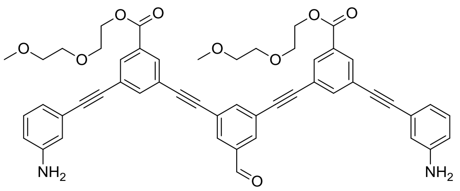

ss-**ABA**

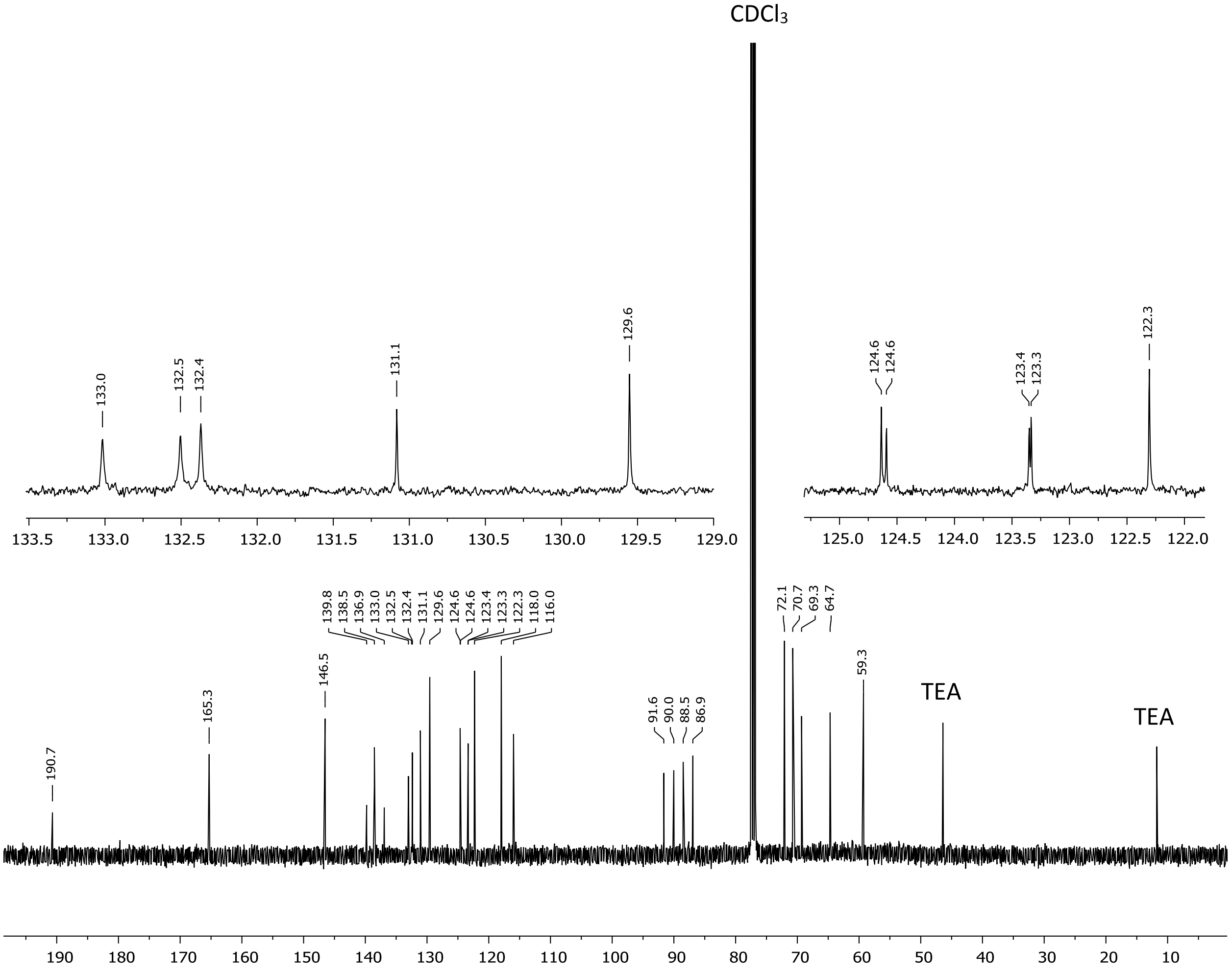

^{1}H NMR (400 MHz, $CDCl_3$)

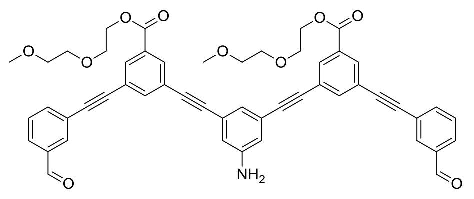

ss-**BAB**

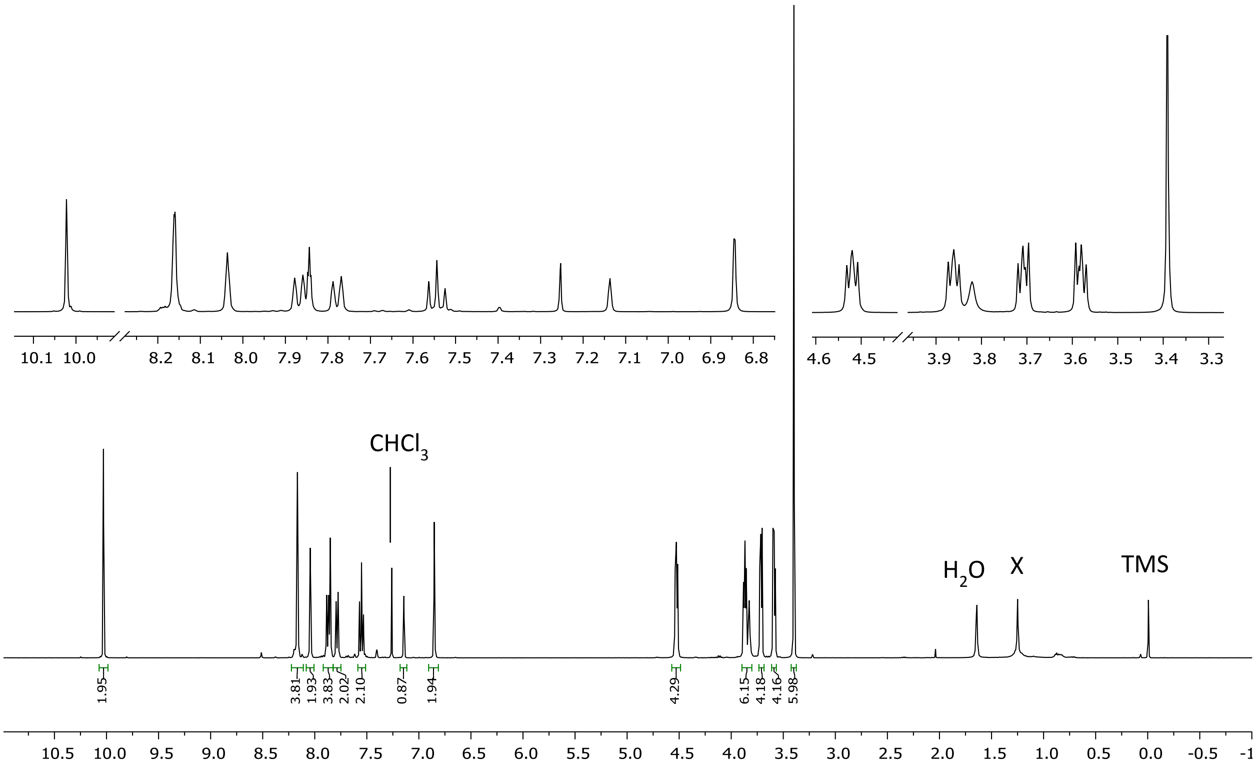

^{13}C NMR (100 MHz, $CDCl_3$)

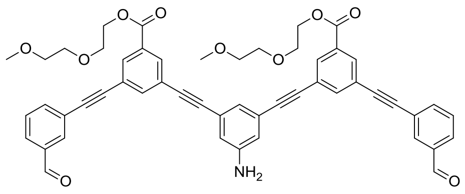

ss-**BAB**

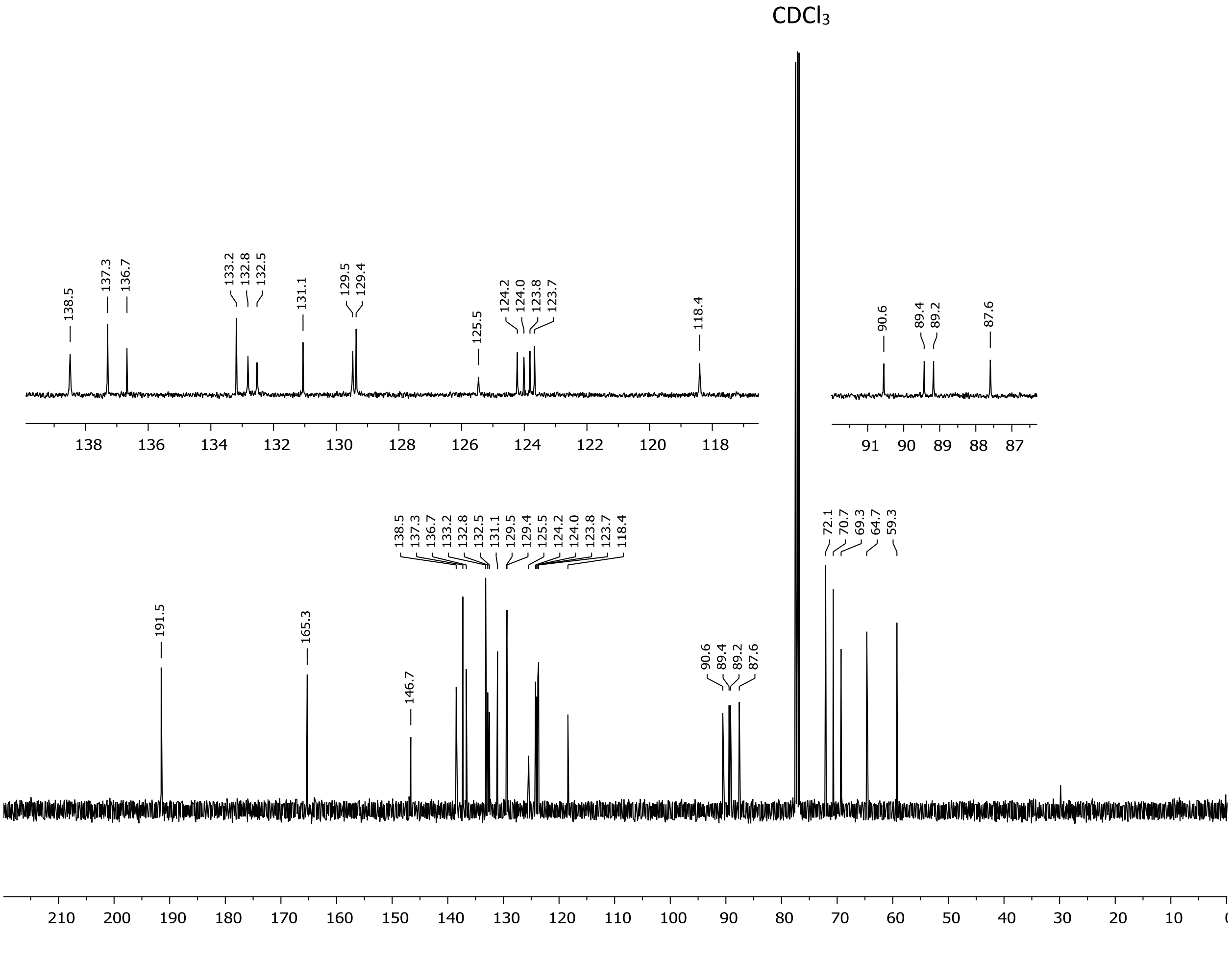

^{1}H NMR (400 MHz, $CDCl_3$ w/ 0.1% TEA)

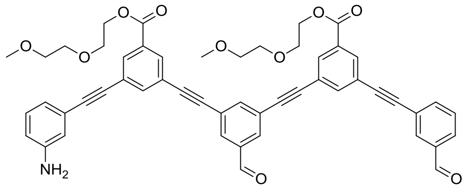

ss-**ABB**

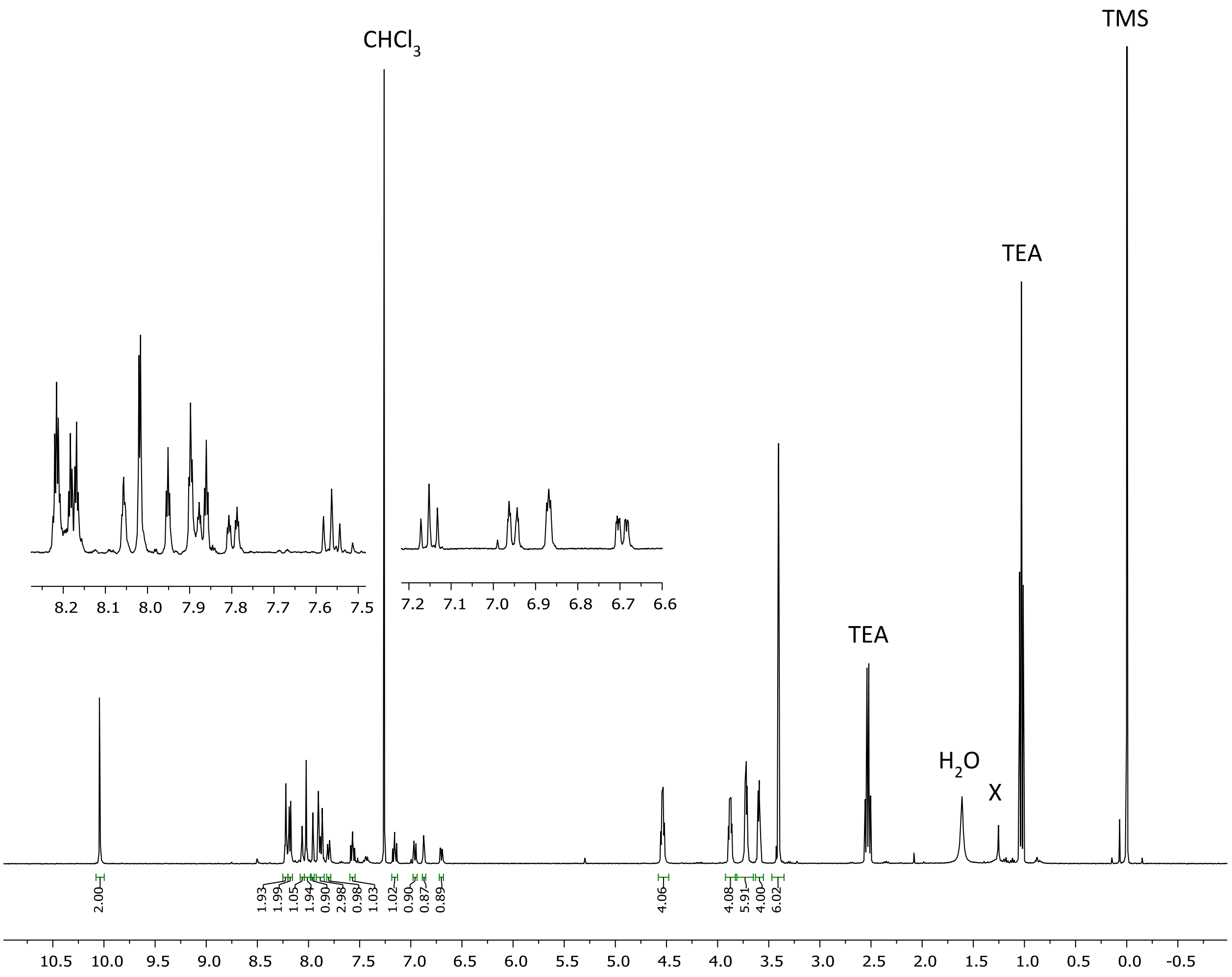

^{13}C NMR (100 MHz, $CDCl_3$ w/ 0.1% TEA)

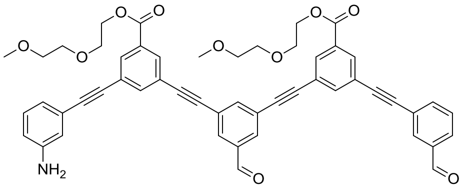

***ss*-ABB**

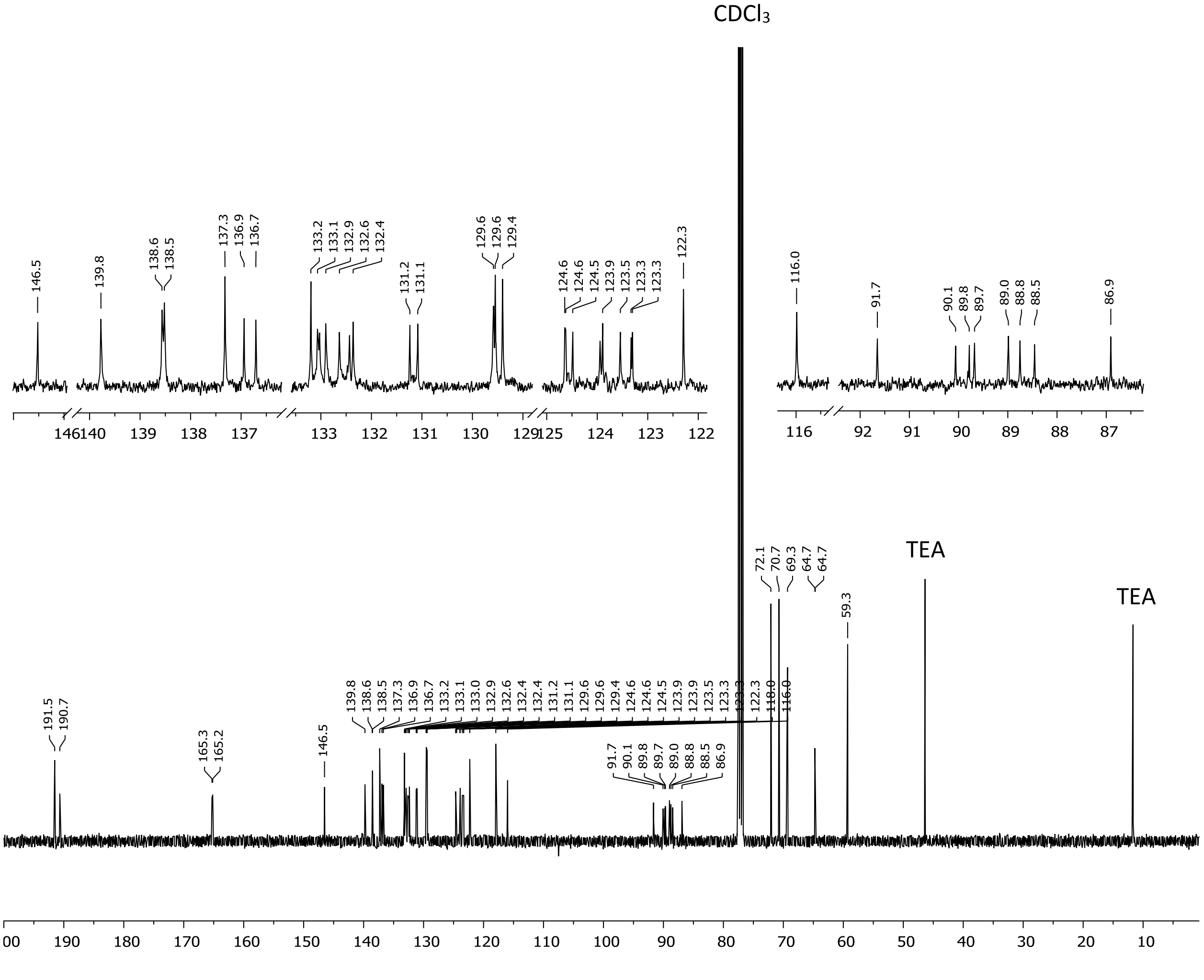

^{1}H NMR (400 MHz, $CDCl_3$ w/ 0.1% TEA)

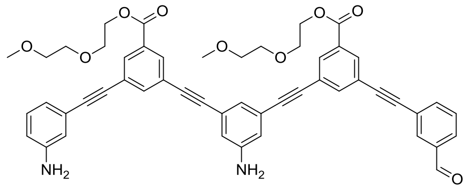

ss-**AAB**

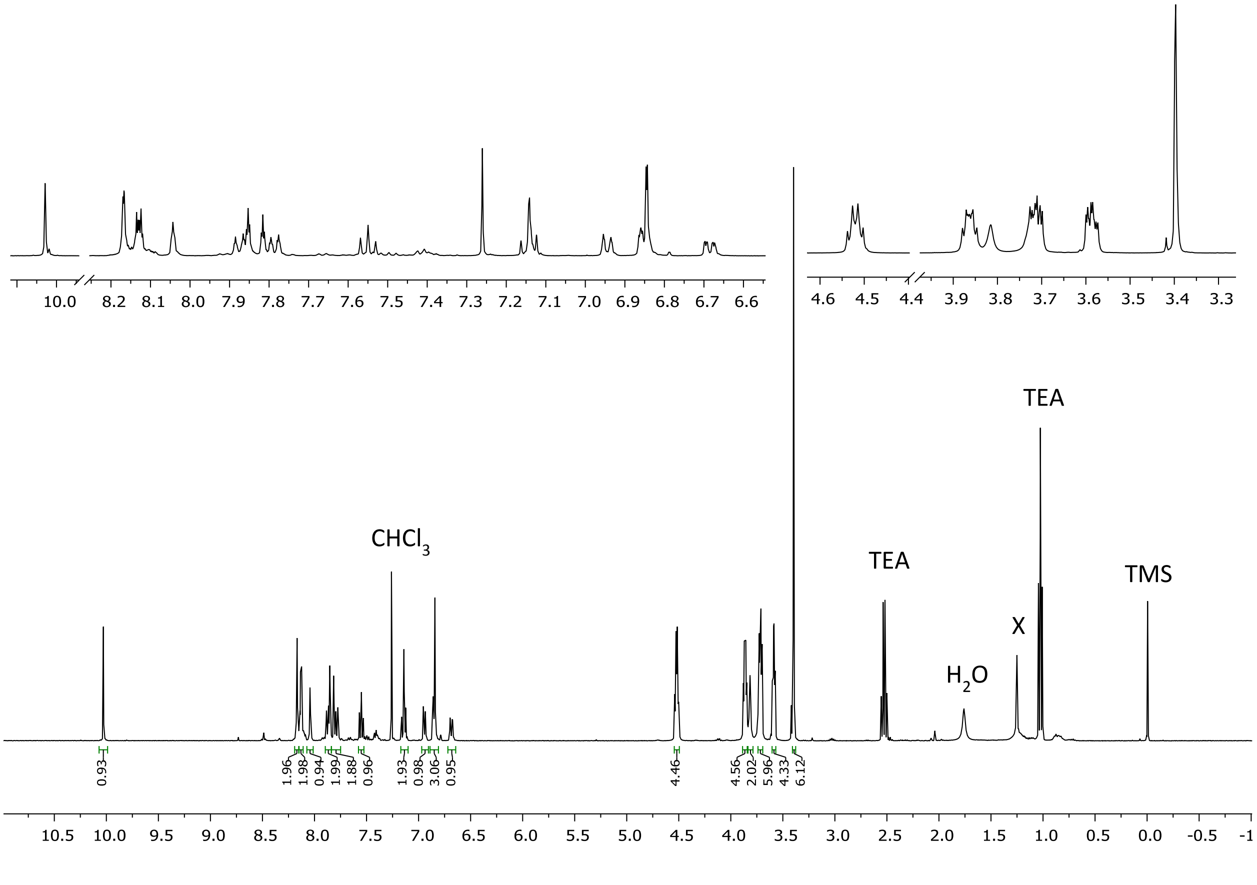

^{13}C NMR (100 MHz, $CDCl_3$ w/ 0.1% TEA)

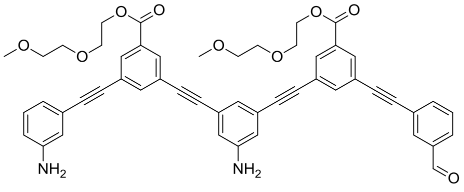

ss-**AAB**

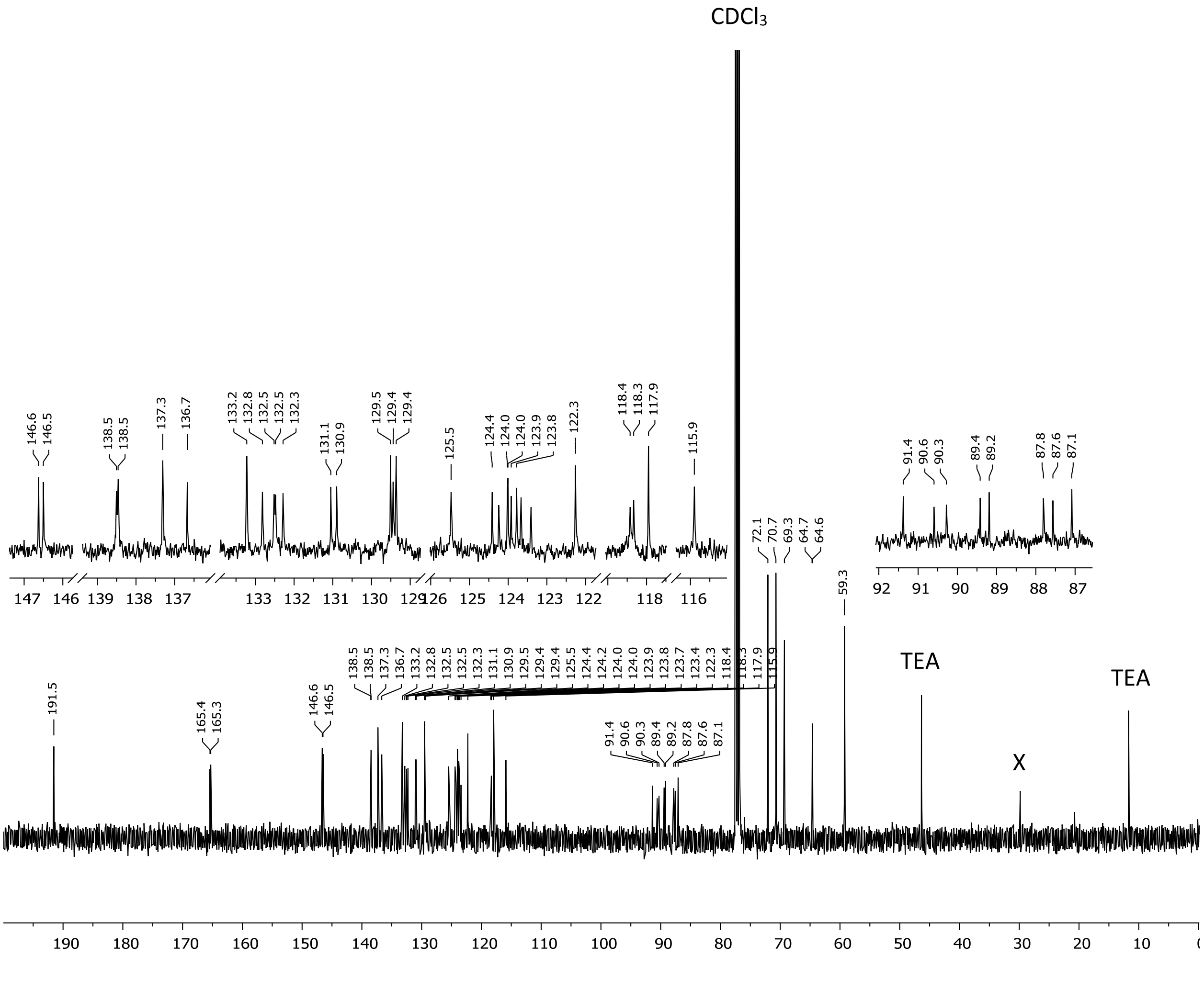

^{1}H NMR (400 MHz, $CDCl_3$ w/ 0.1% TEA)

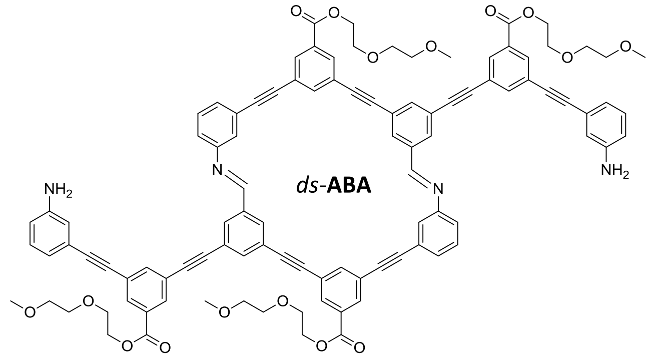

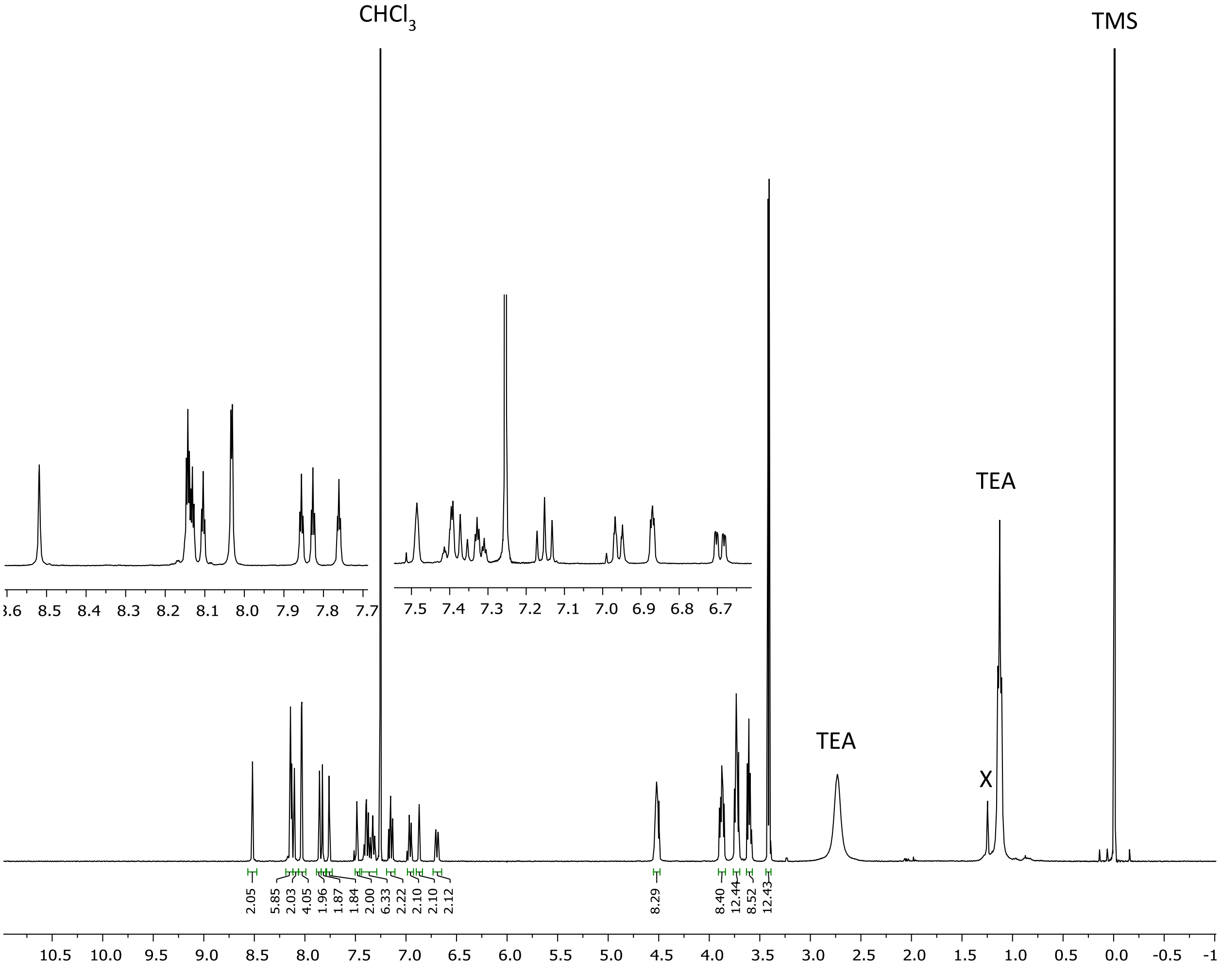

^{13}C NMR (100 MHz, $CDCl_3$ w/ 0.1% TEA)

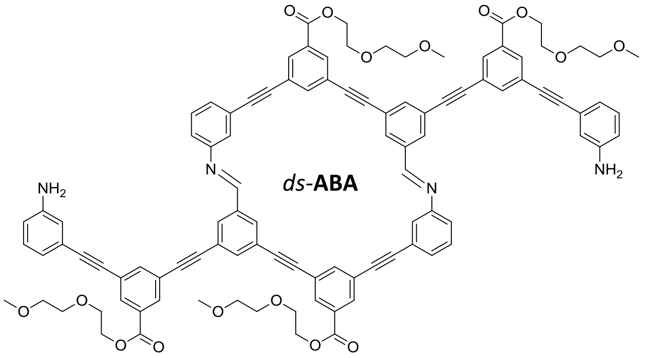

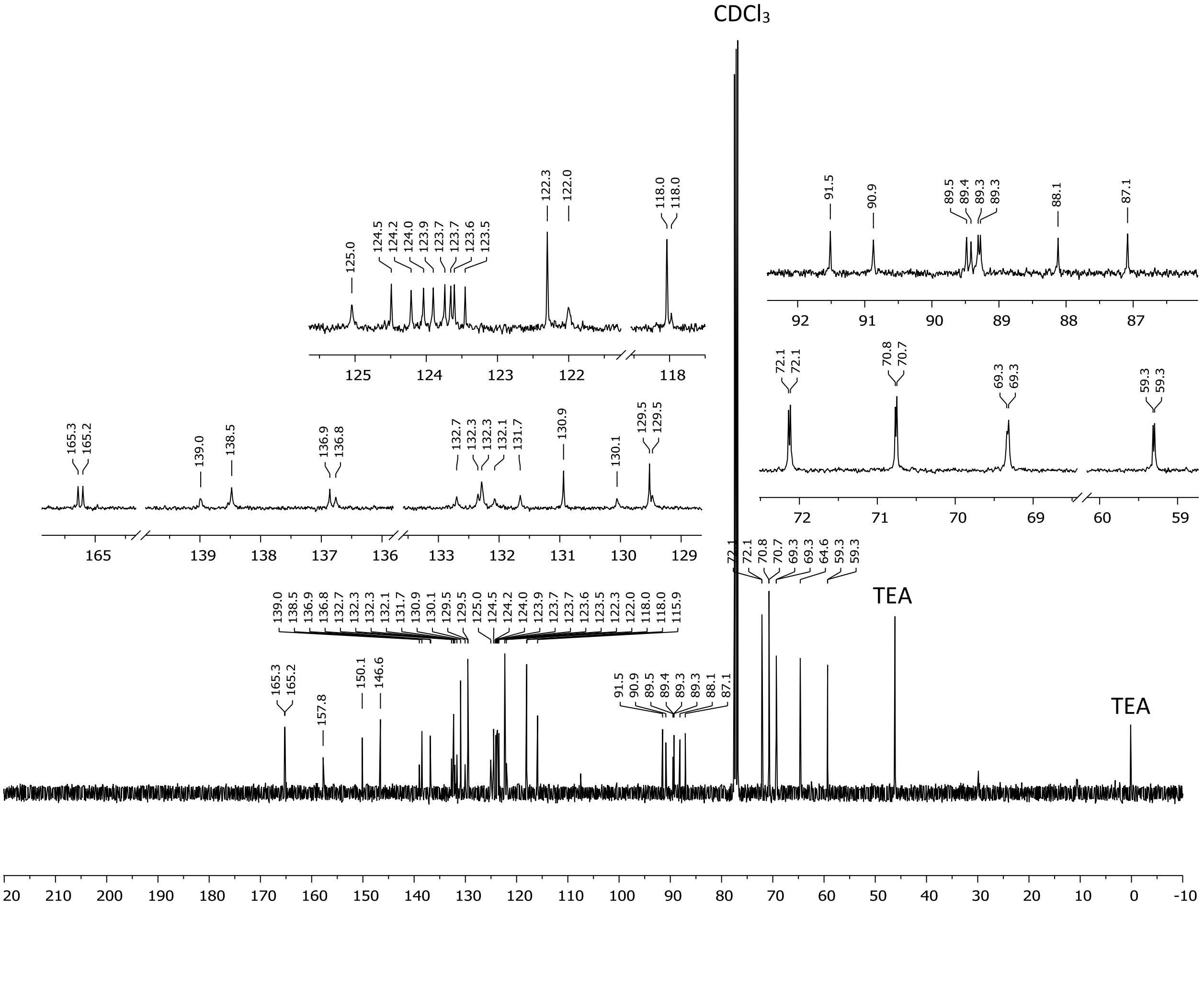

^1H-^1H ROSEY NMR (400 MHz, $CDCl_3$ w/ 0.1% TEA)

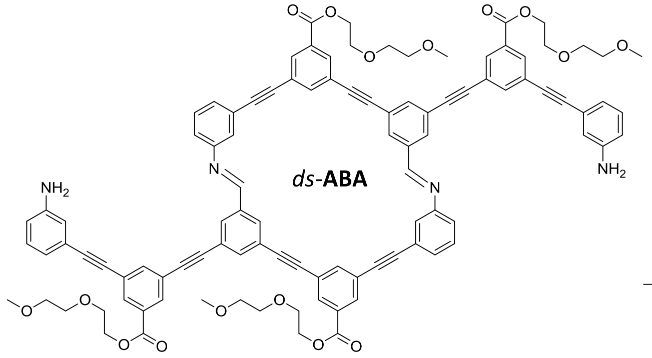

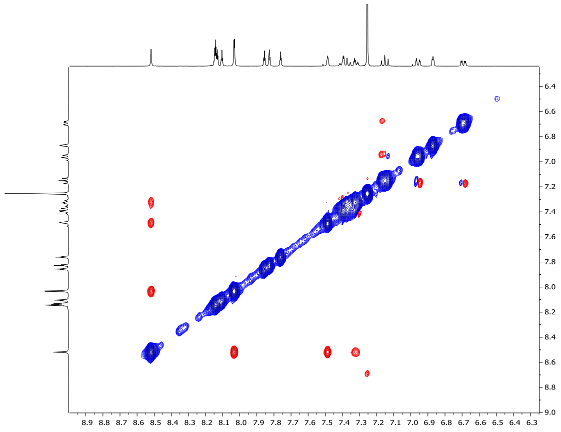

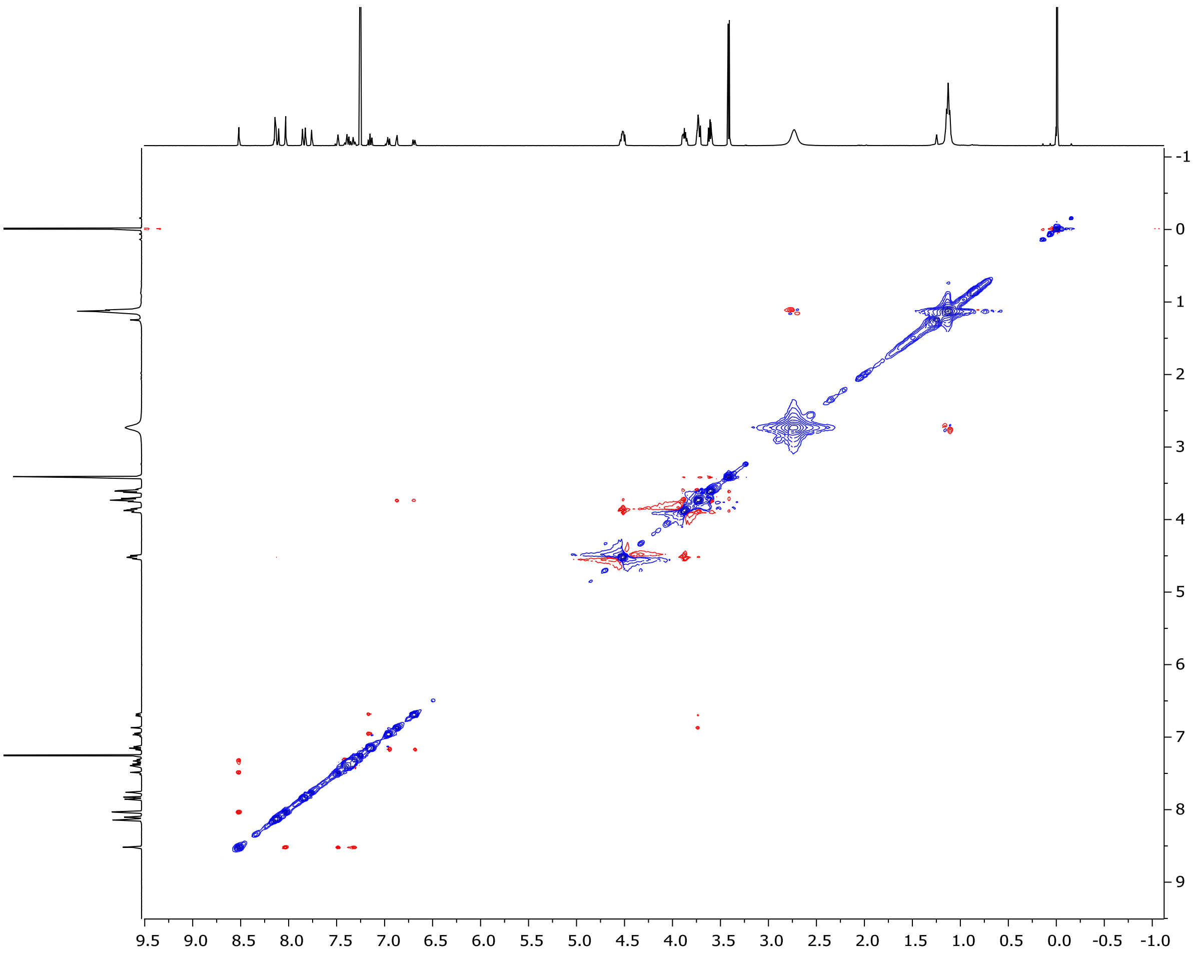

^{1}H-^{1}H COSY NMR (400 MHz, $CDCl_3$ w/ 0.1% TEA)

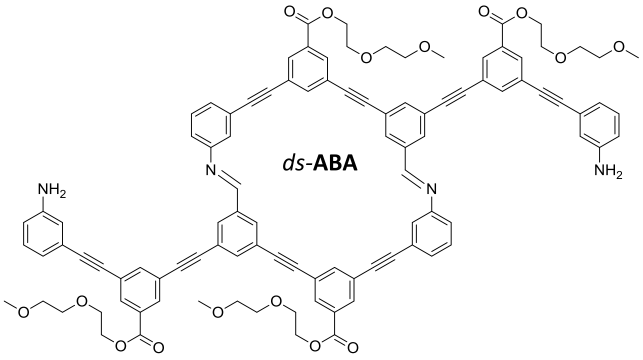

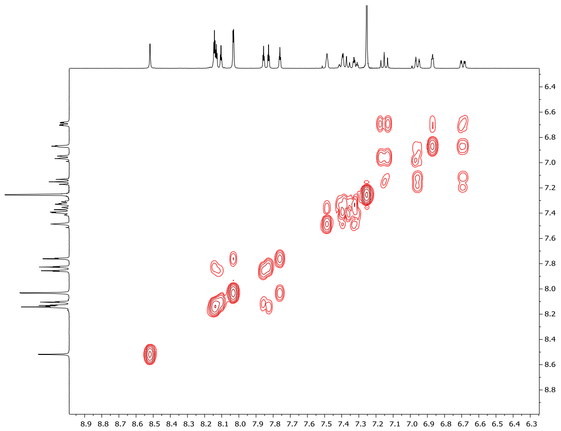

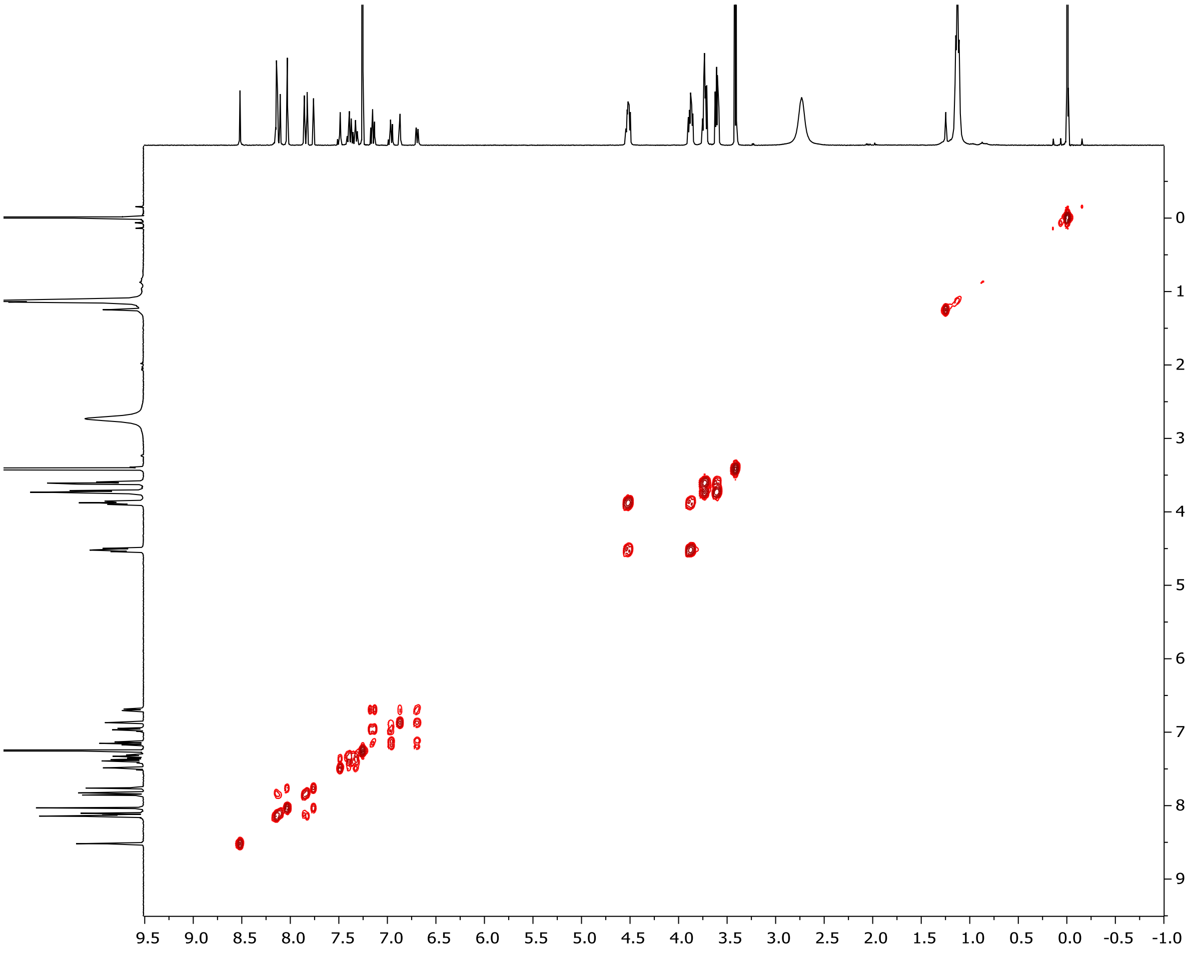

^{1}H NMR (400 MHz, $CDCl_3$ w/ 0.1% TEA)

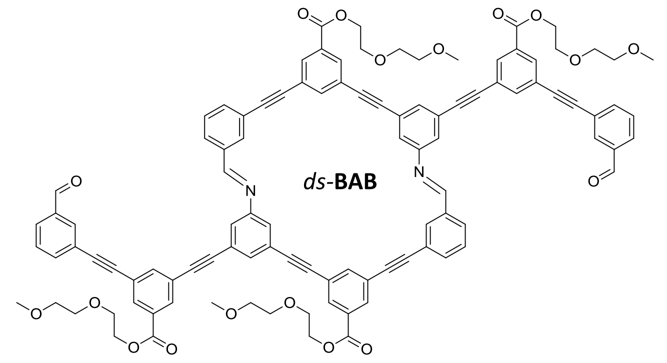

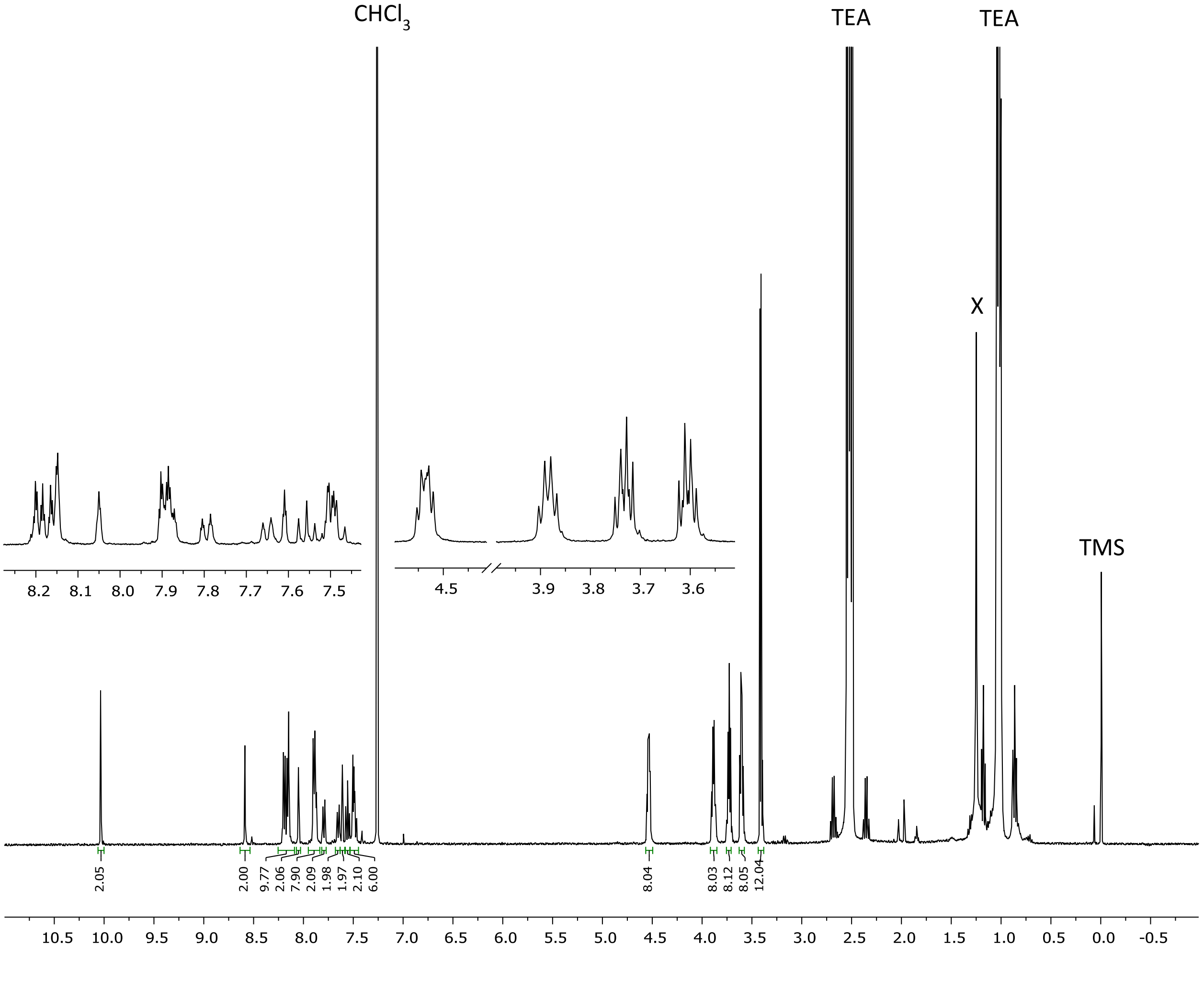

^{13}C NMR (100 MHz, $CDCl_3$ w/ 0.1% TEA)

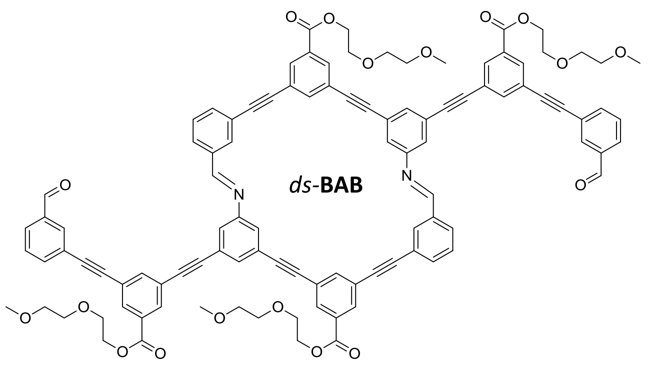

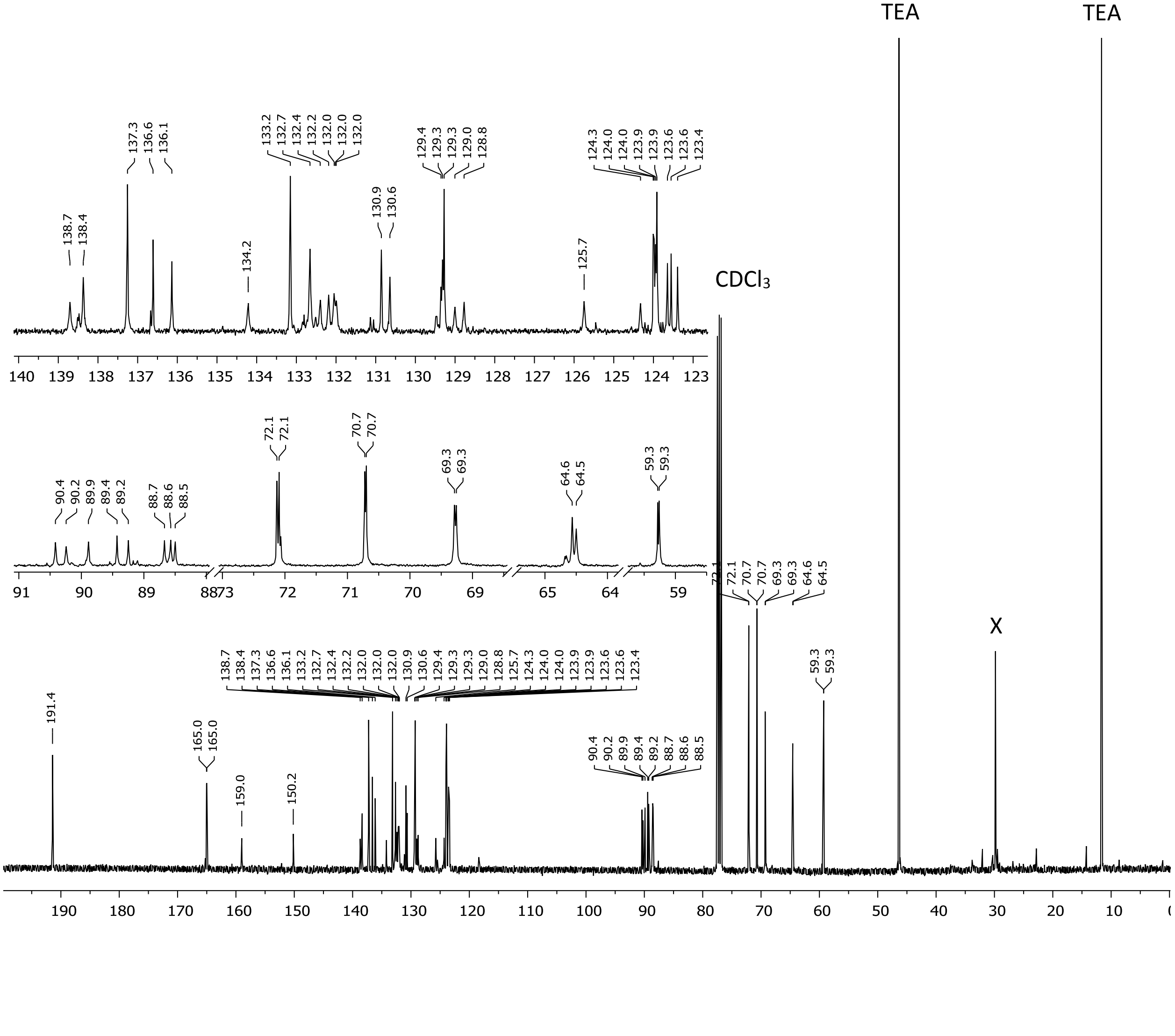

^{1}H-^{1}H ROSEY NMR (400 MHz, $CDCl_3$ w/ 0.1% TEA)

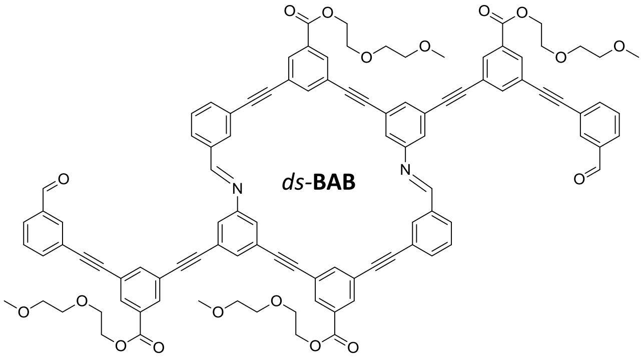

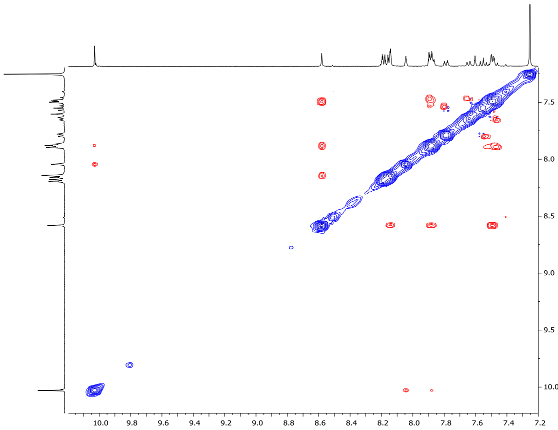

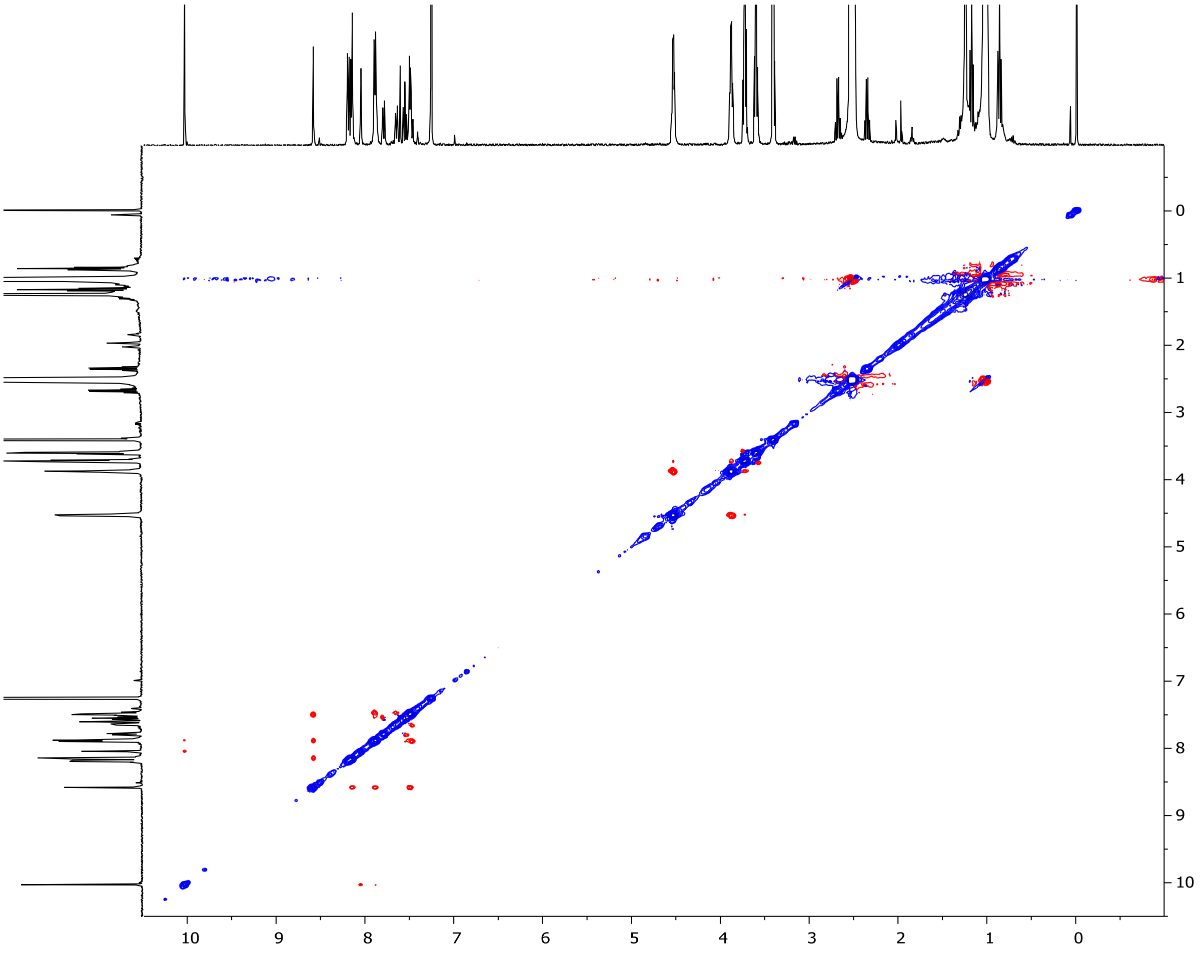

^{1}H-^{1}H COSY NMR (400 MHz, $CDCl_3$ w/ 0.1% TEA)

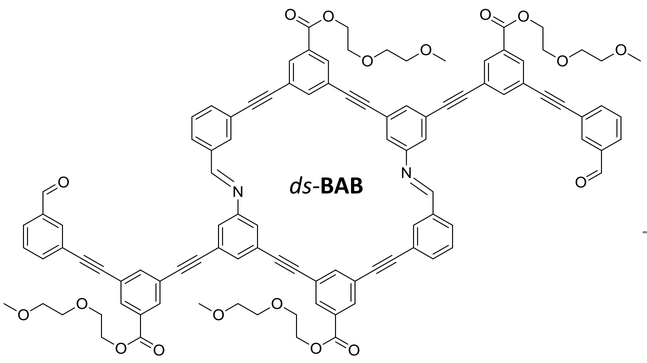

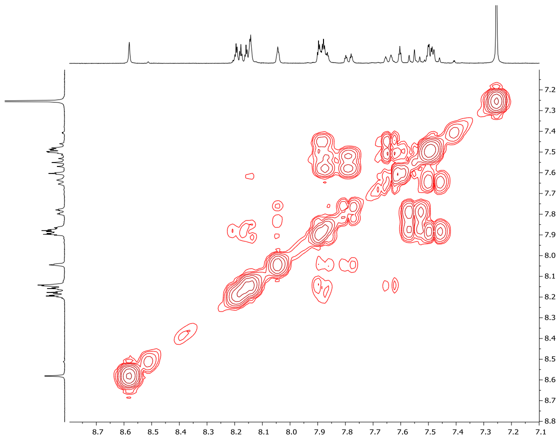

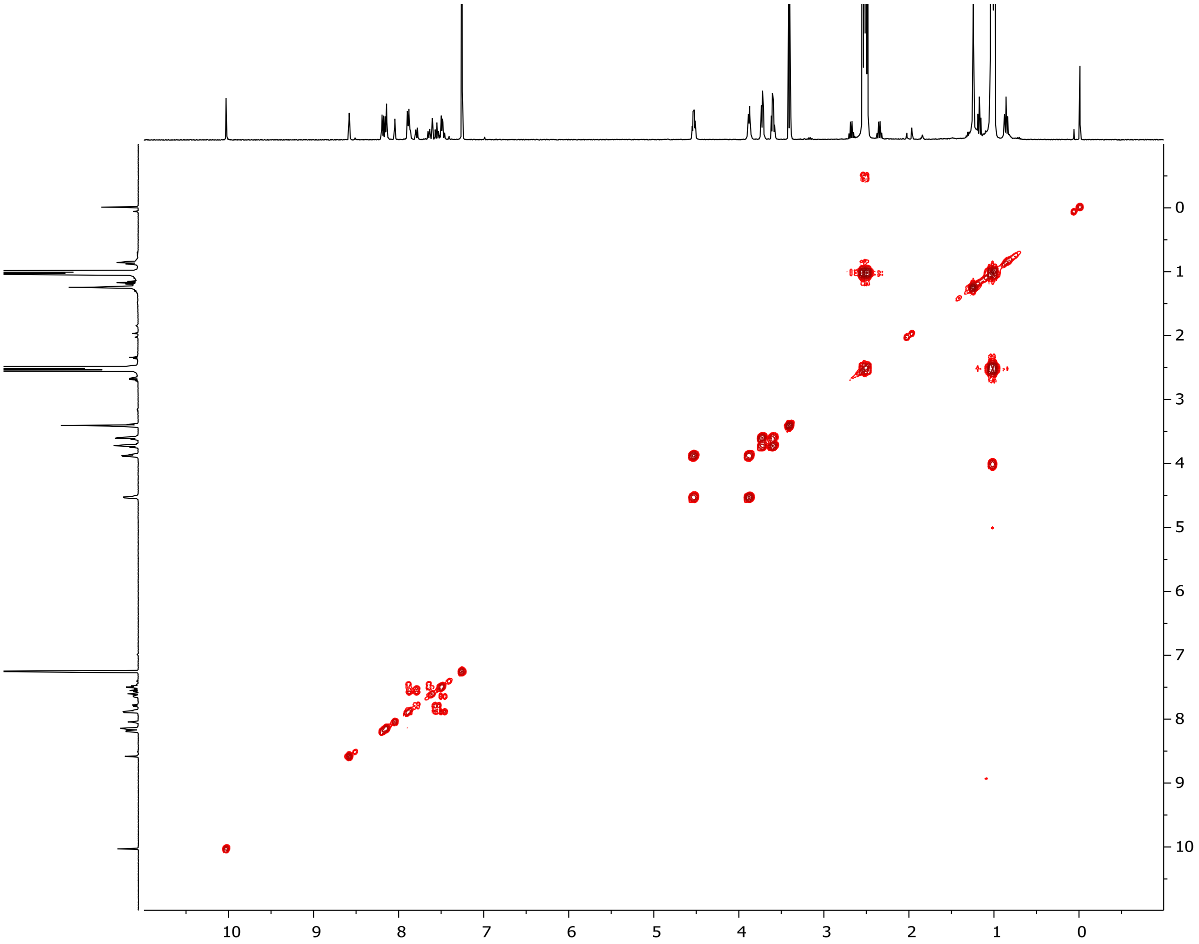

^{1}H NMR (400 MHz, $CDCl_3$ w/ 0.1% TEA)

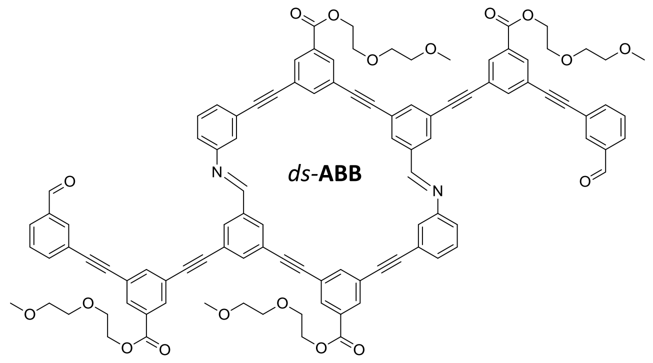

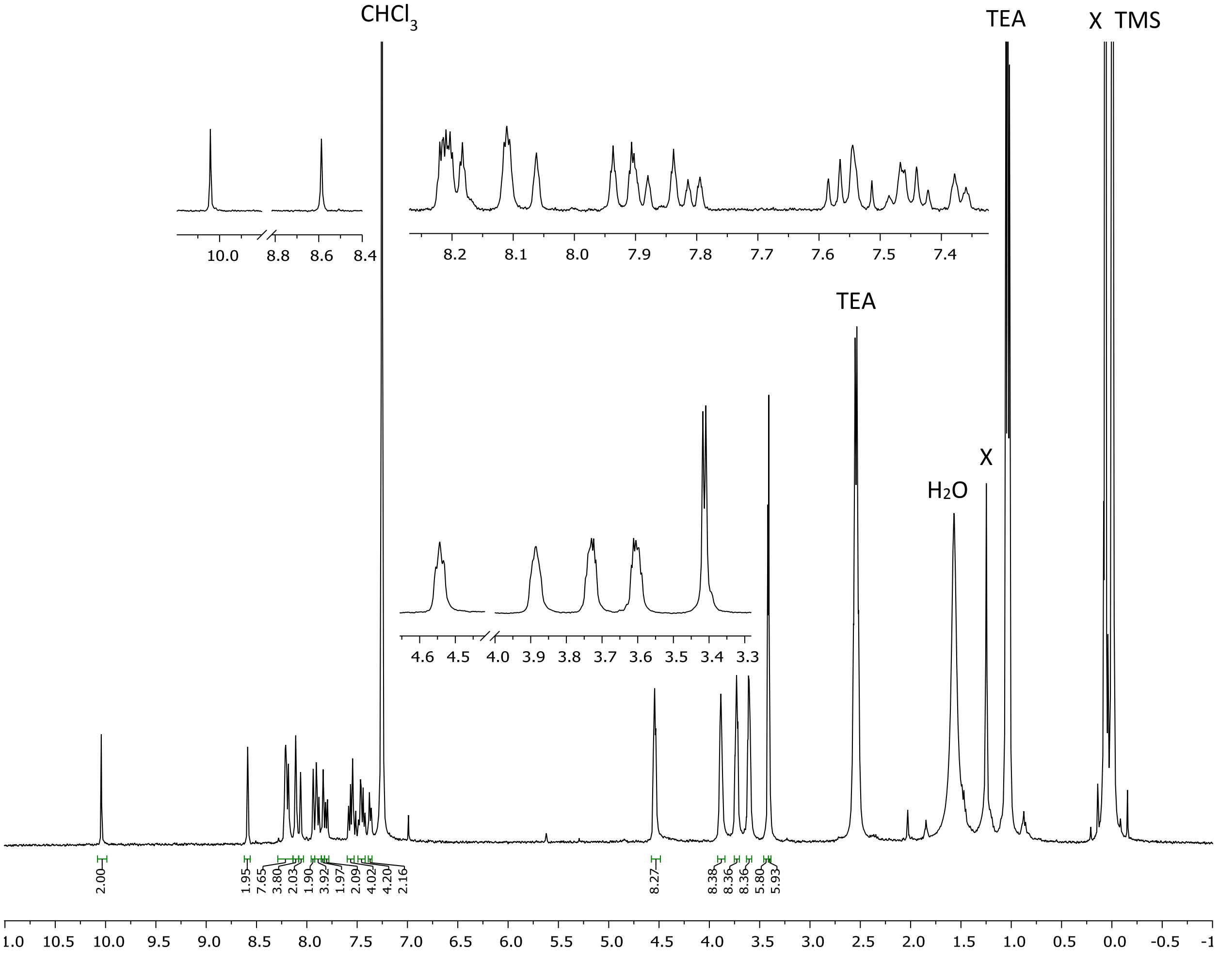

^{13}C NMR (100 MHz, $CDCl_3$ w/ 0.1% TEA)

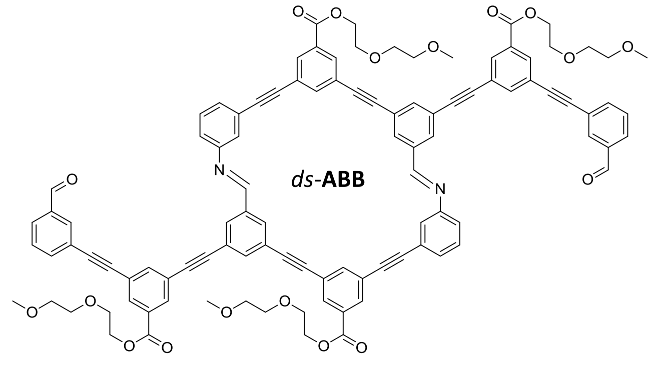

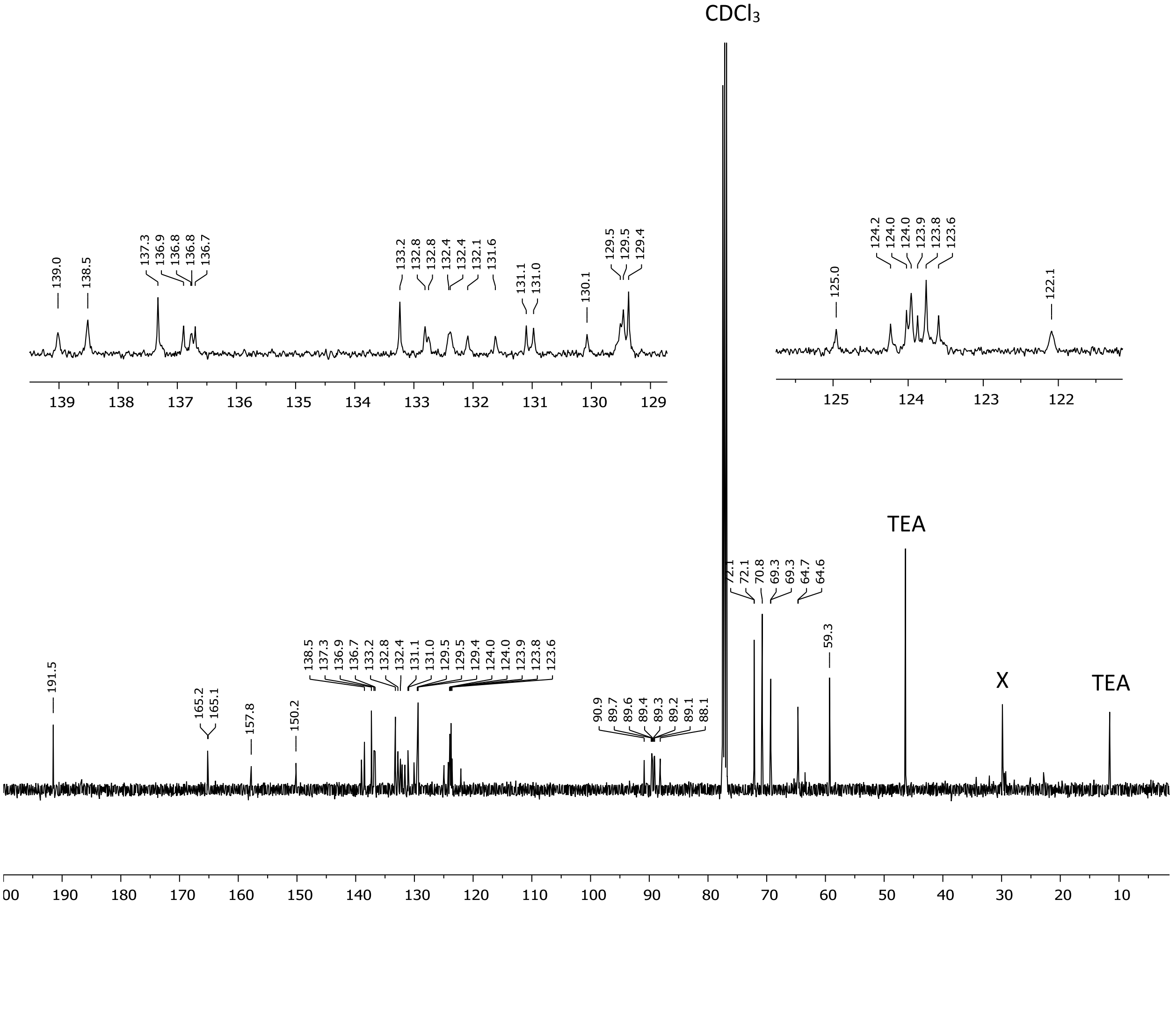

^{1}H-^{1}H ROSEY NMR (400 MHz, $CDCl_3$ w/ 0.1% TEA)

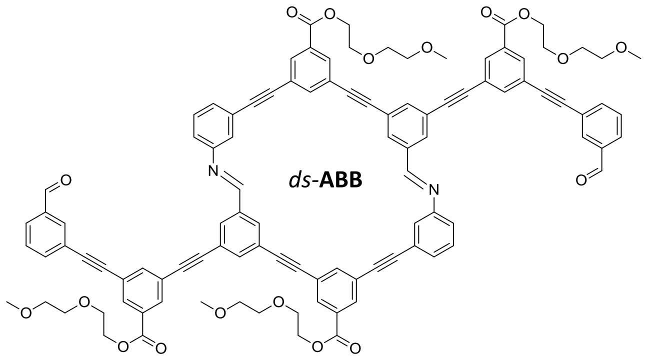

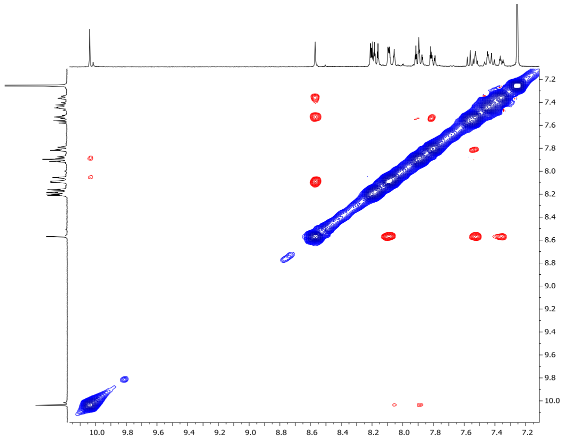

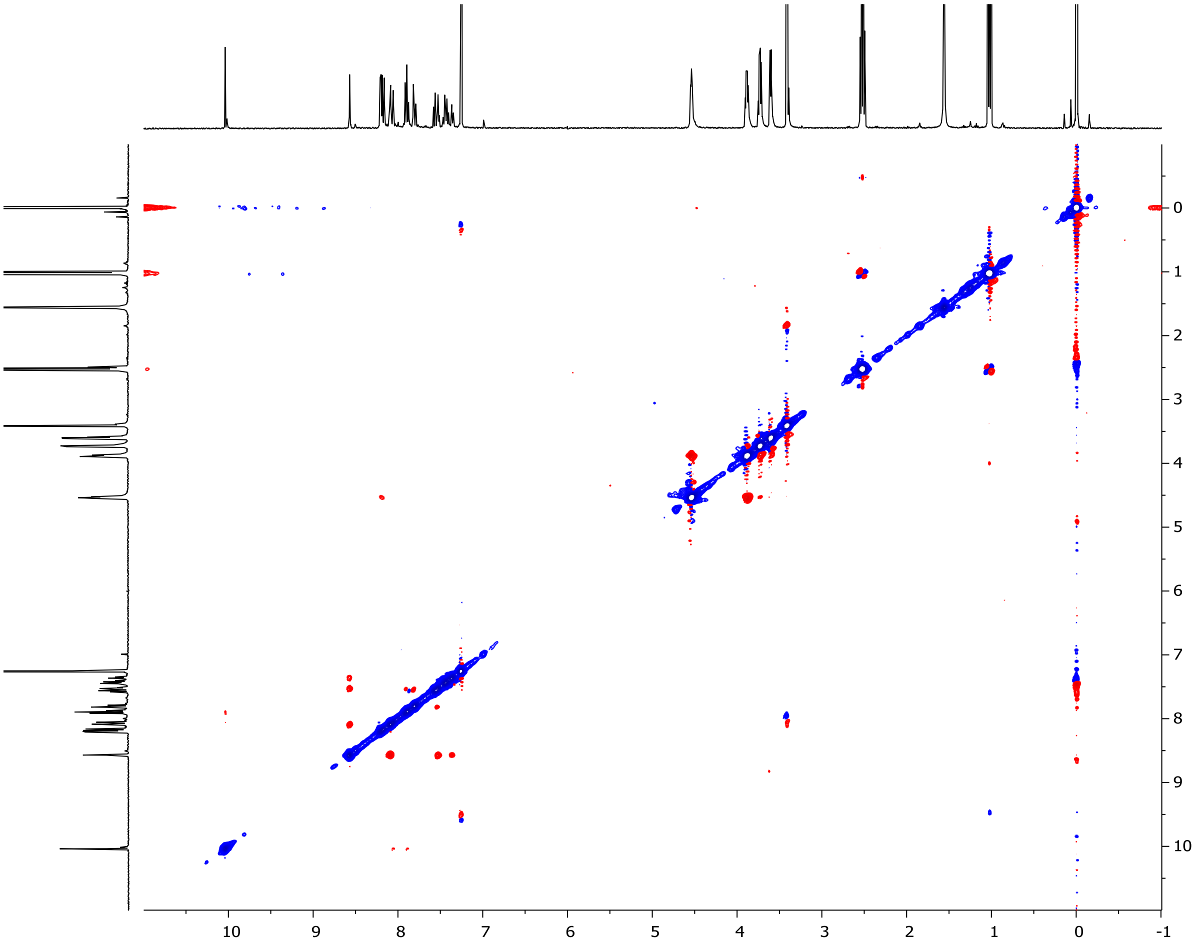

^{1}H-^{1}H COSY NMR (400 MHz, $CDCl_3$ w/ 0.1% TEA)

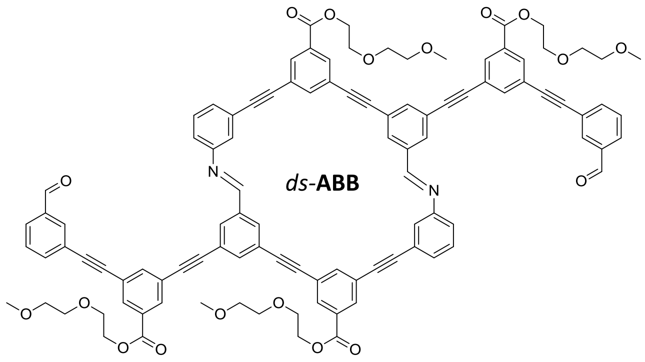

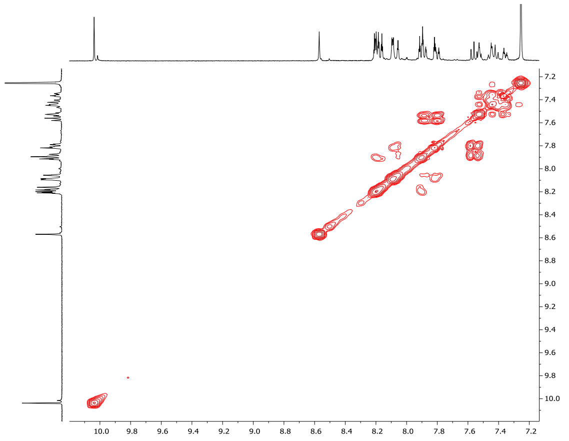

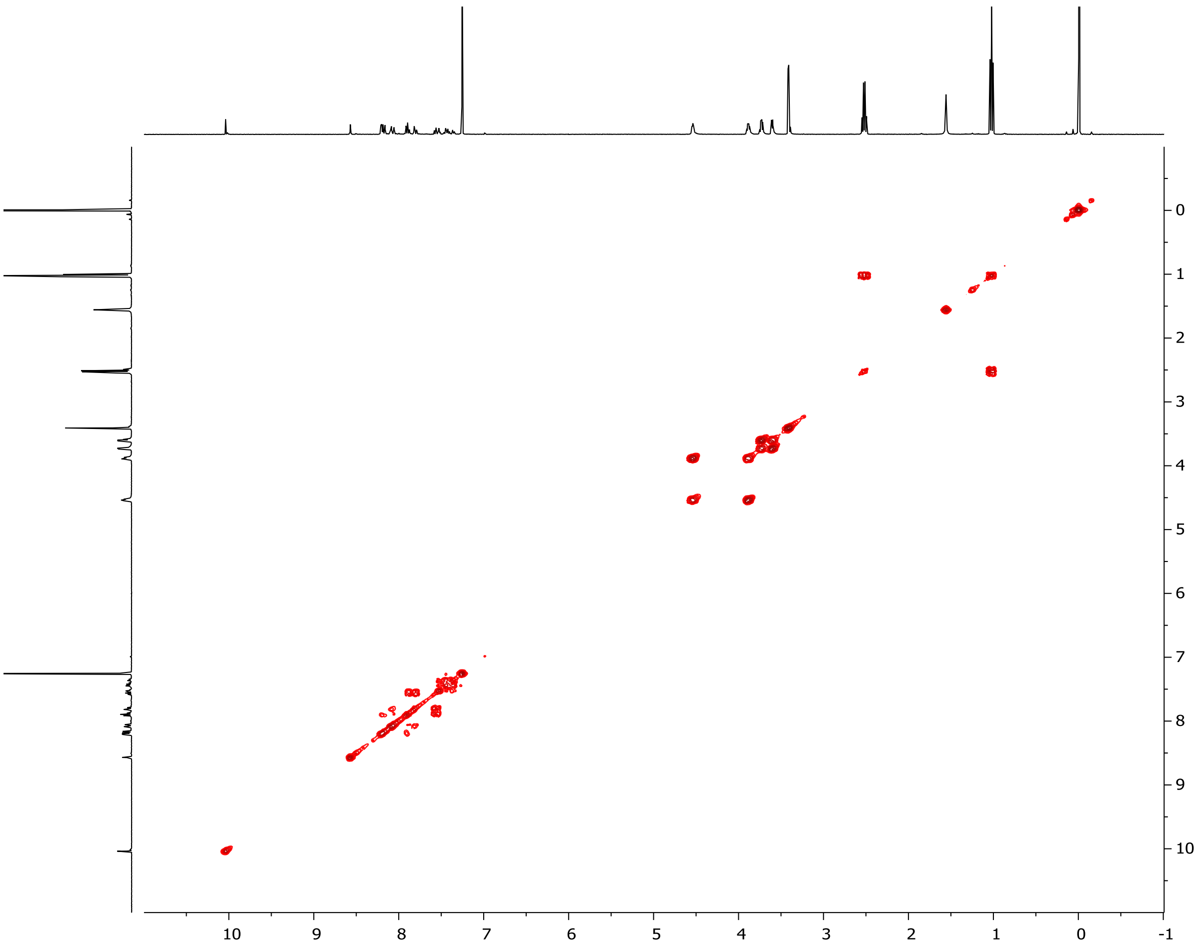

^{1}H NMR (400 MHz, $CDCl_3$ w/ 0.1% TEA)

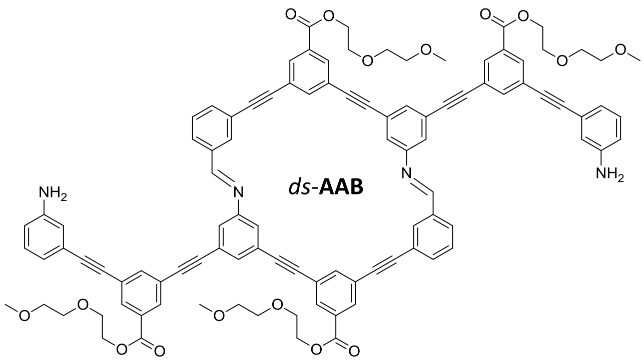

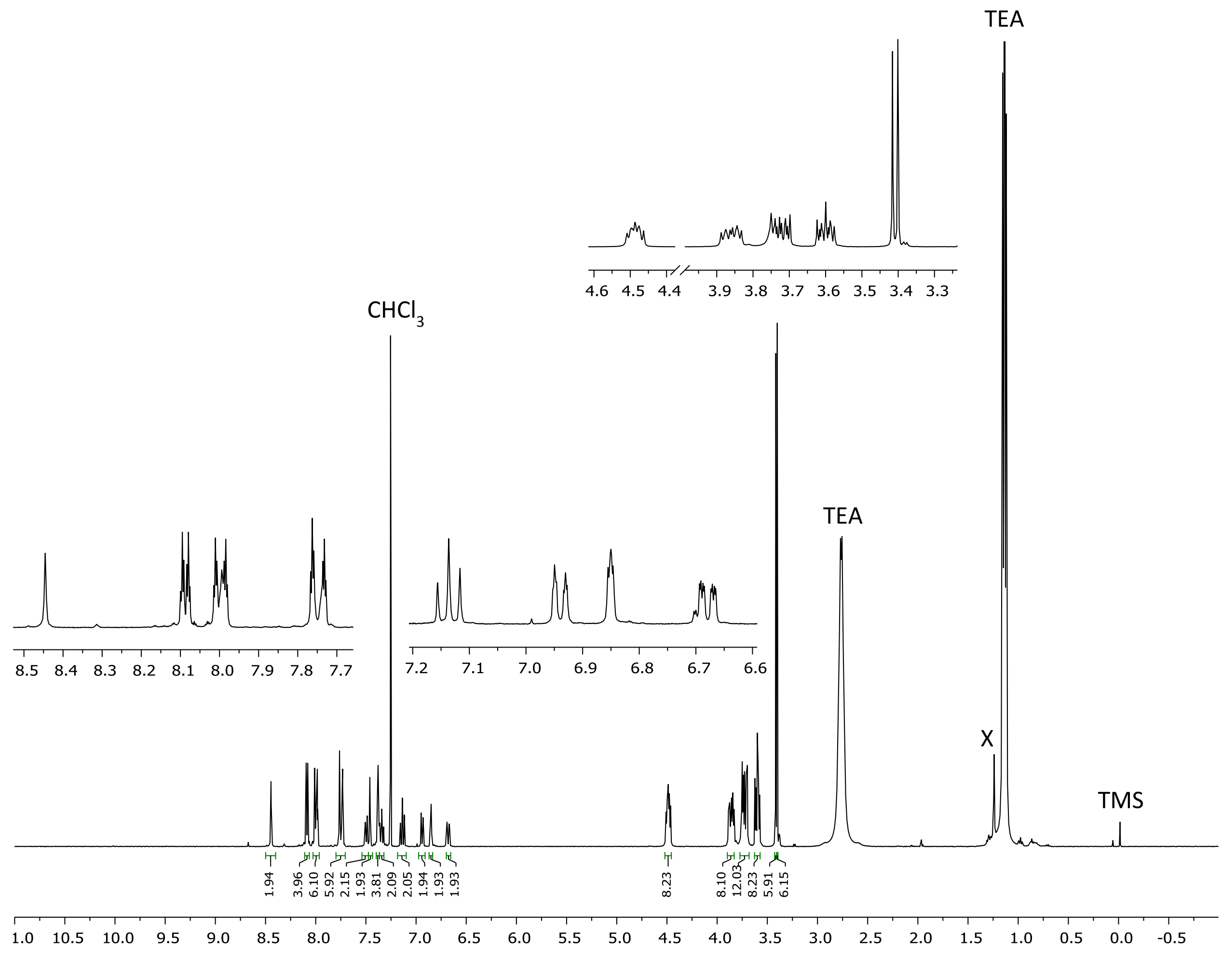

^{13}C NMR (100 MHz, $CDCl_3$ w/ 0.1% TEA)

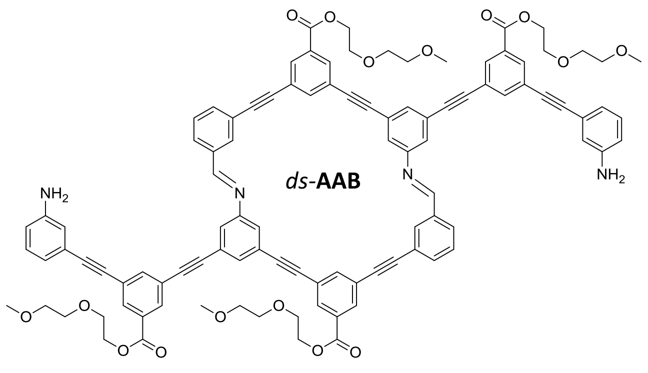

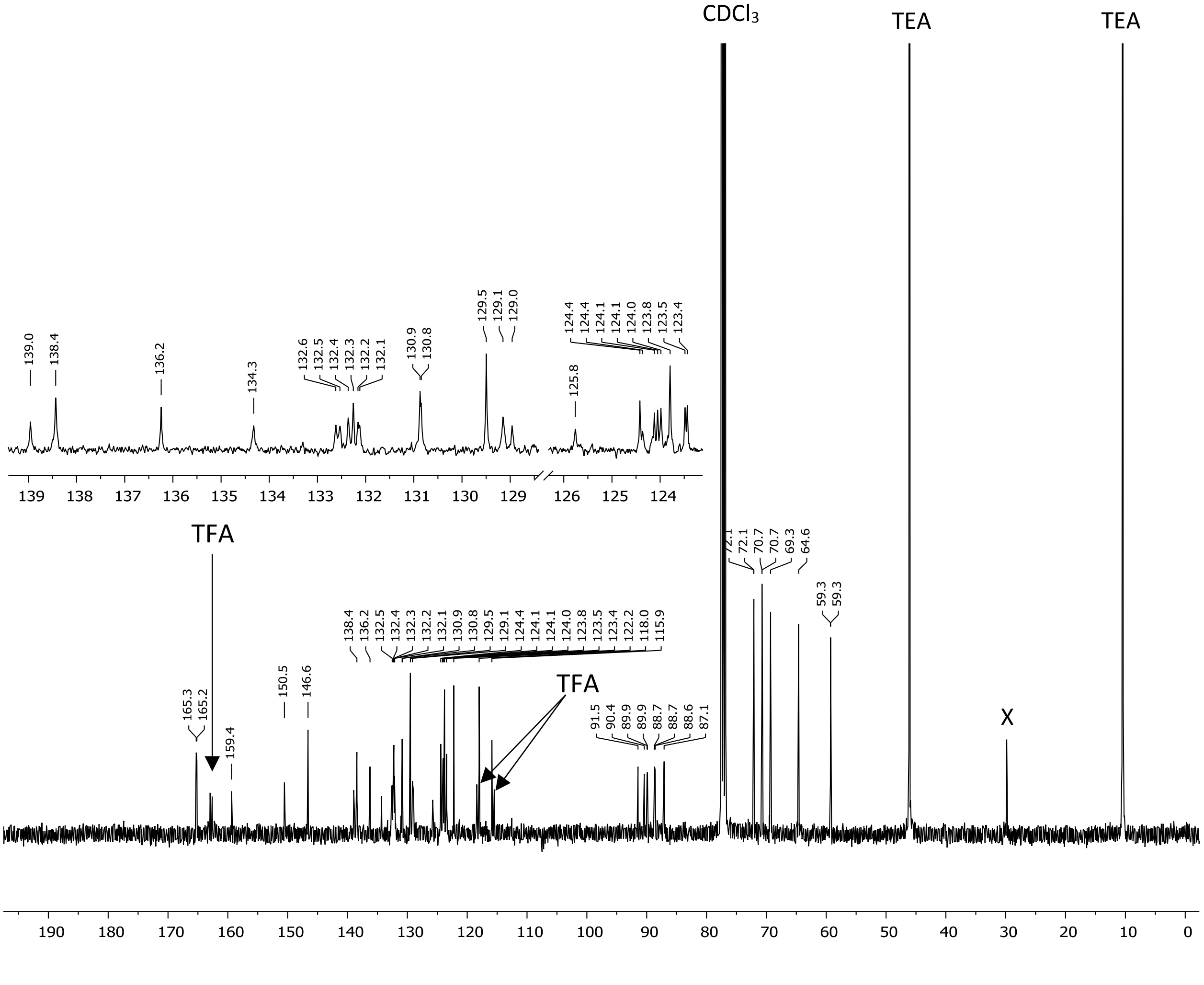

^{1}H-^{1}H ROSEY NMR(400 MHz, $CDCl_3$ w/ 0.1% TEA)

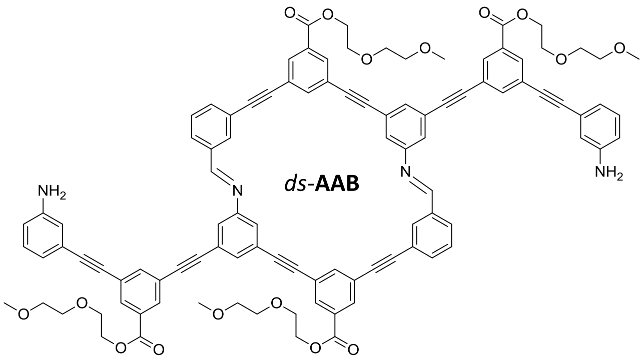

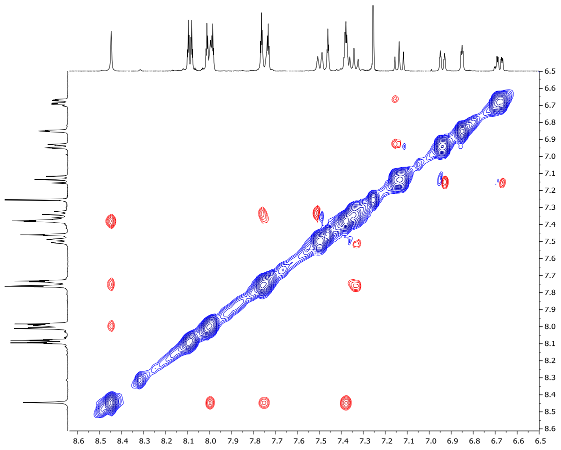

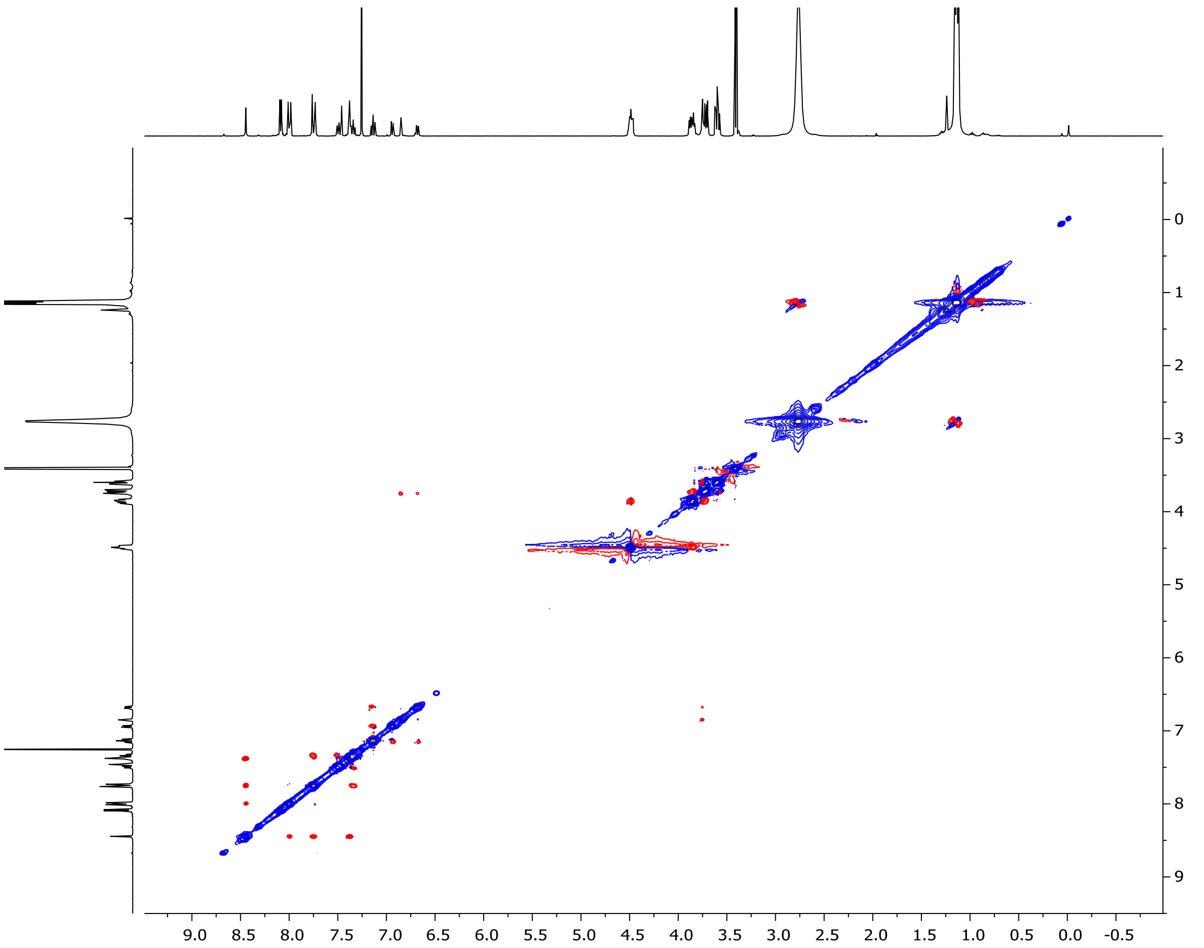

^{1}H-^{1}H COSY NMR(400 MHz, $CDCl_3$ w/ 0.1% TEA)

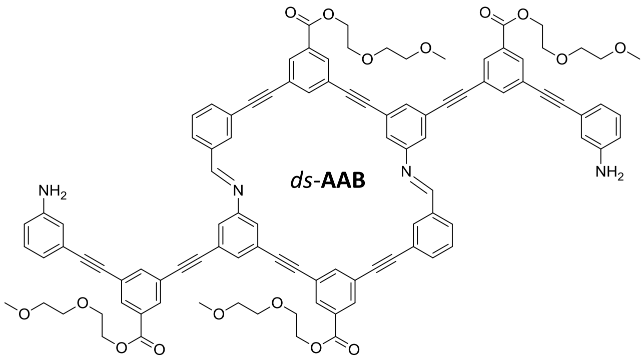

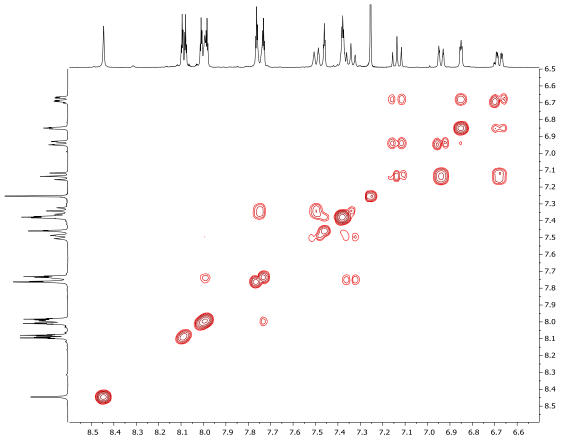

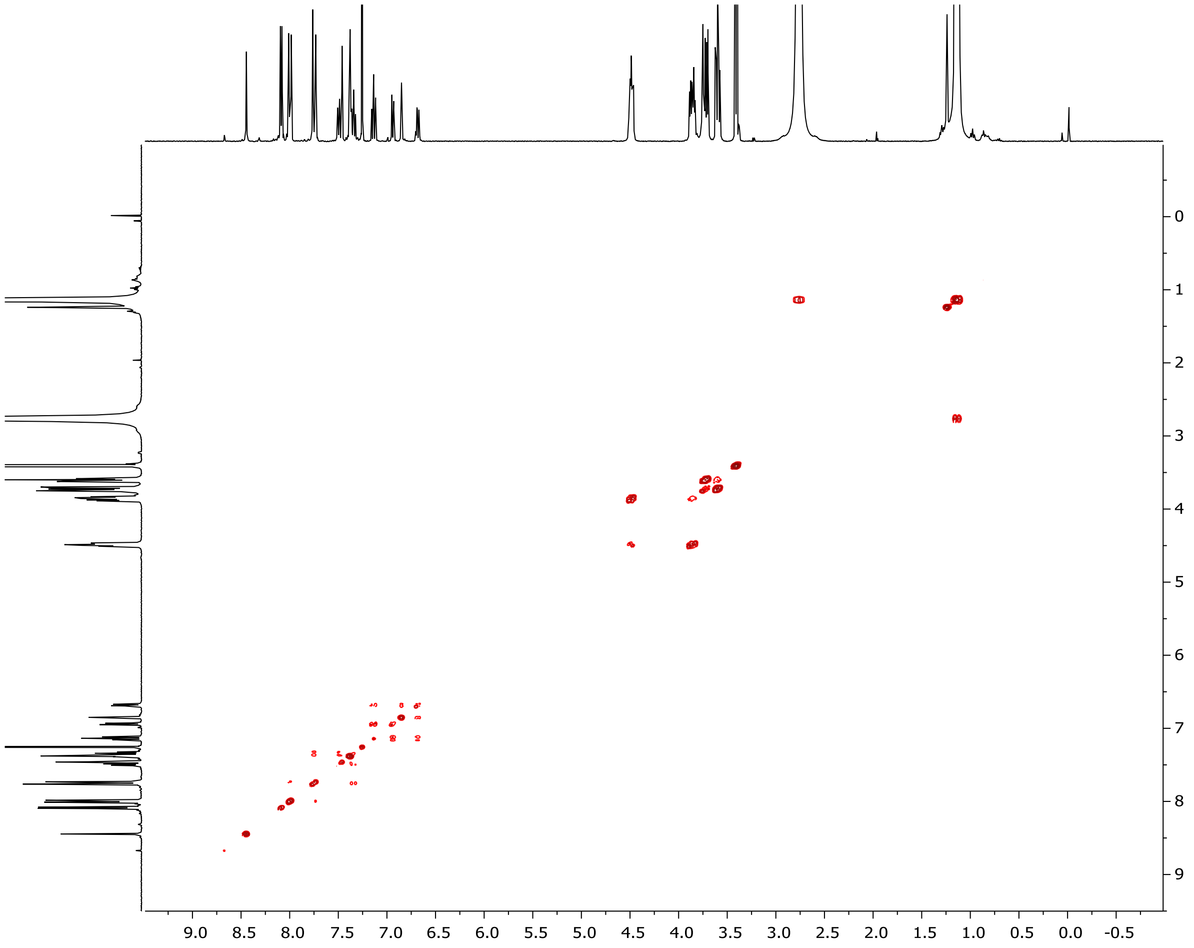

^{1}H NMR (400 MHz, $CDCl_3$ w/ 0.1% TEA)

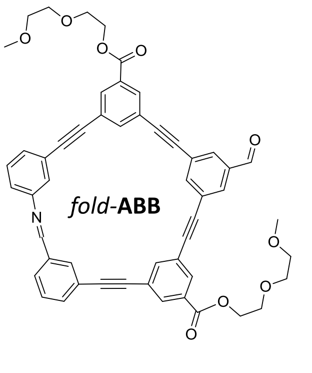

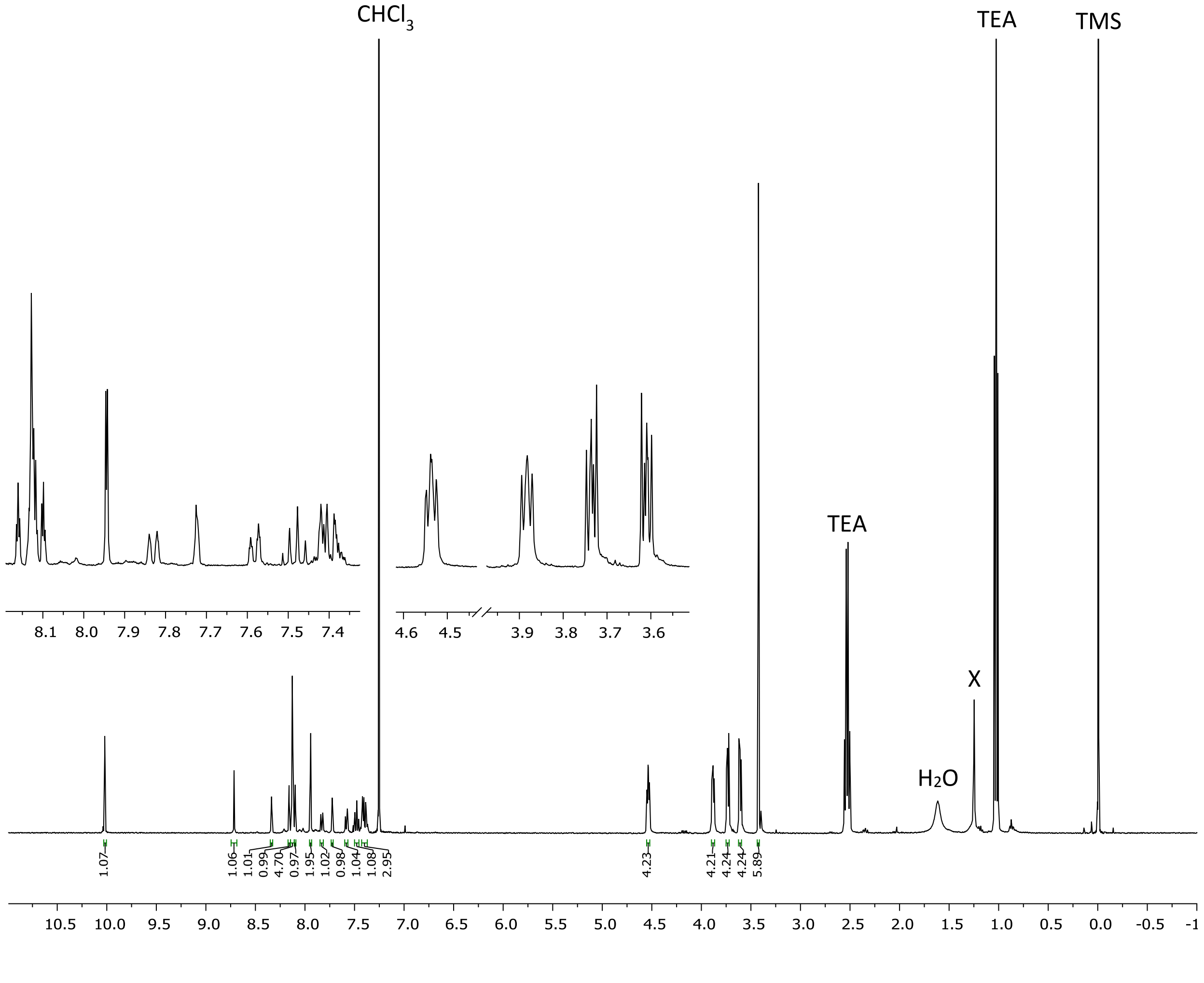

^{13}C NMR (100 MHz, $CDCl_3$ w/ 0.1% TEA)

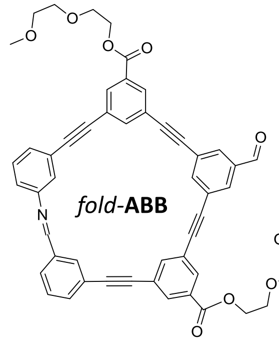

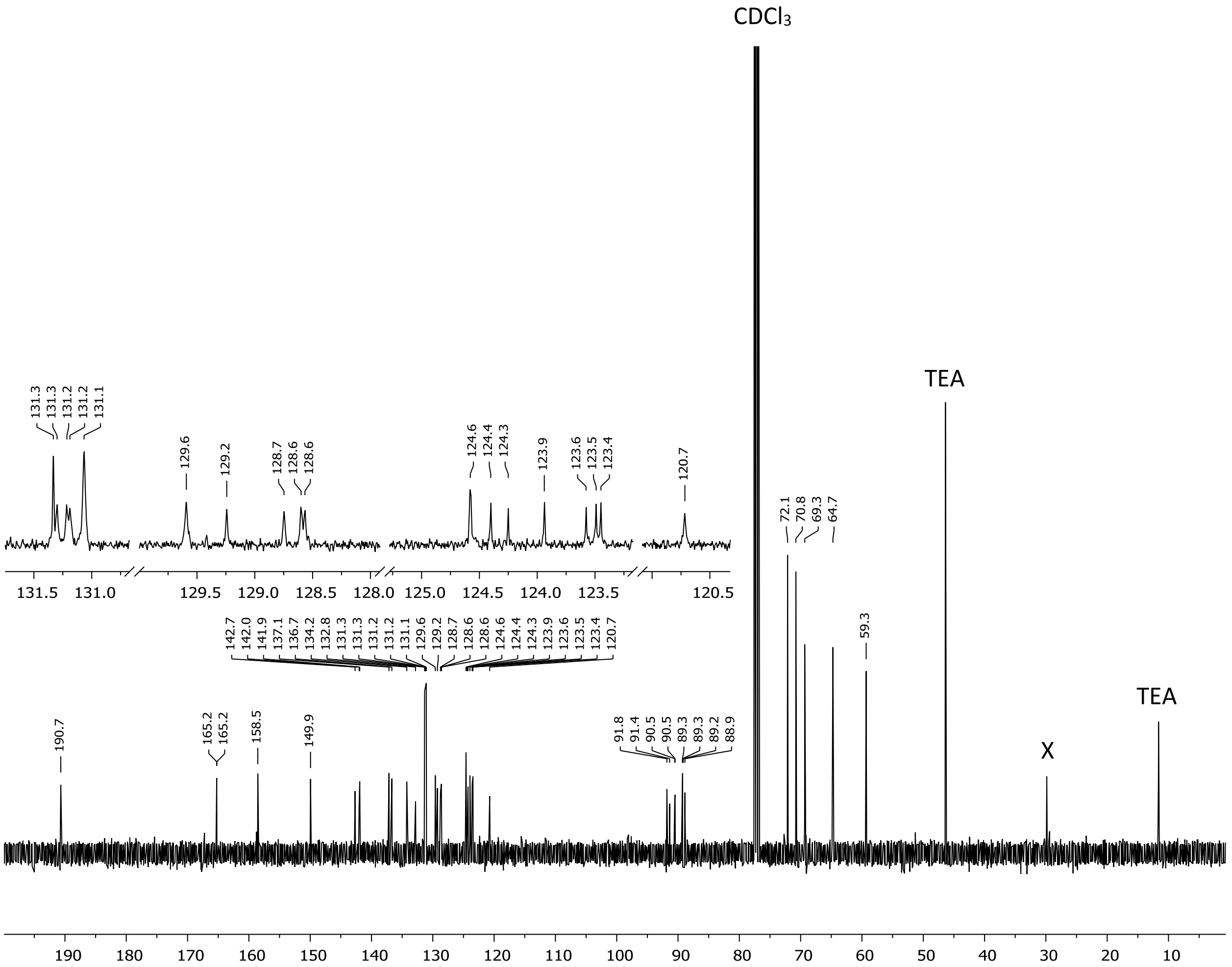

^{1}H-^{1}H ROSEY NMR (400 MHz, $CDCl_3$ w/ 0.1% TEA)

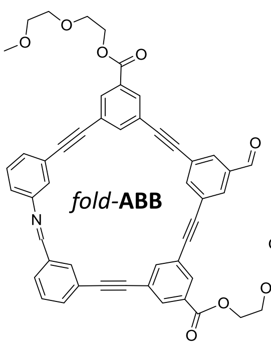

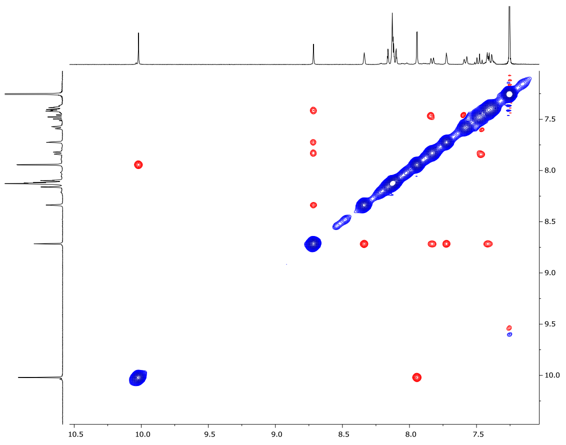

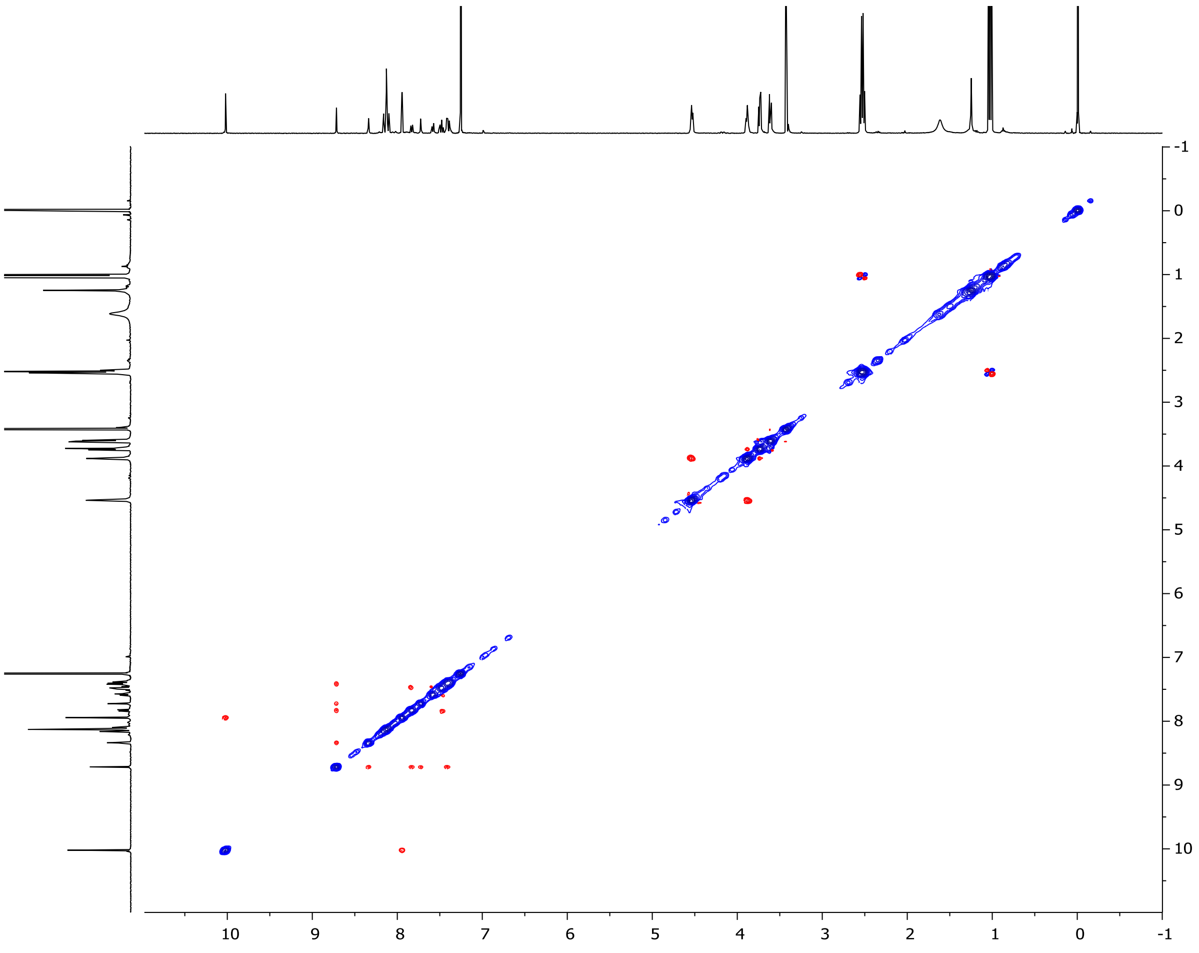

^{1}H-^{1}H COSY NMR (400 MHz, $CDCl_3$ w/ 0.1% TEA)

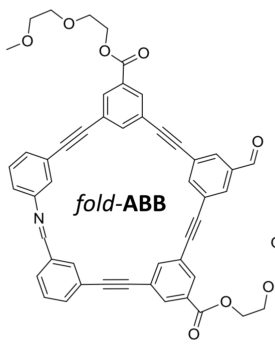

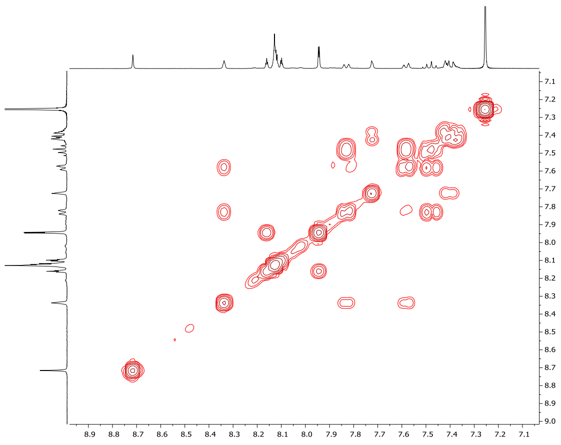

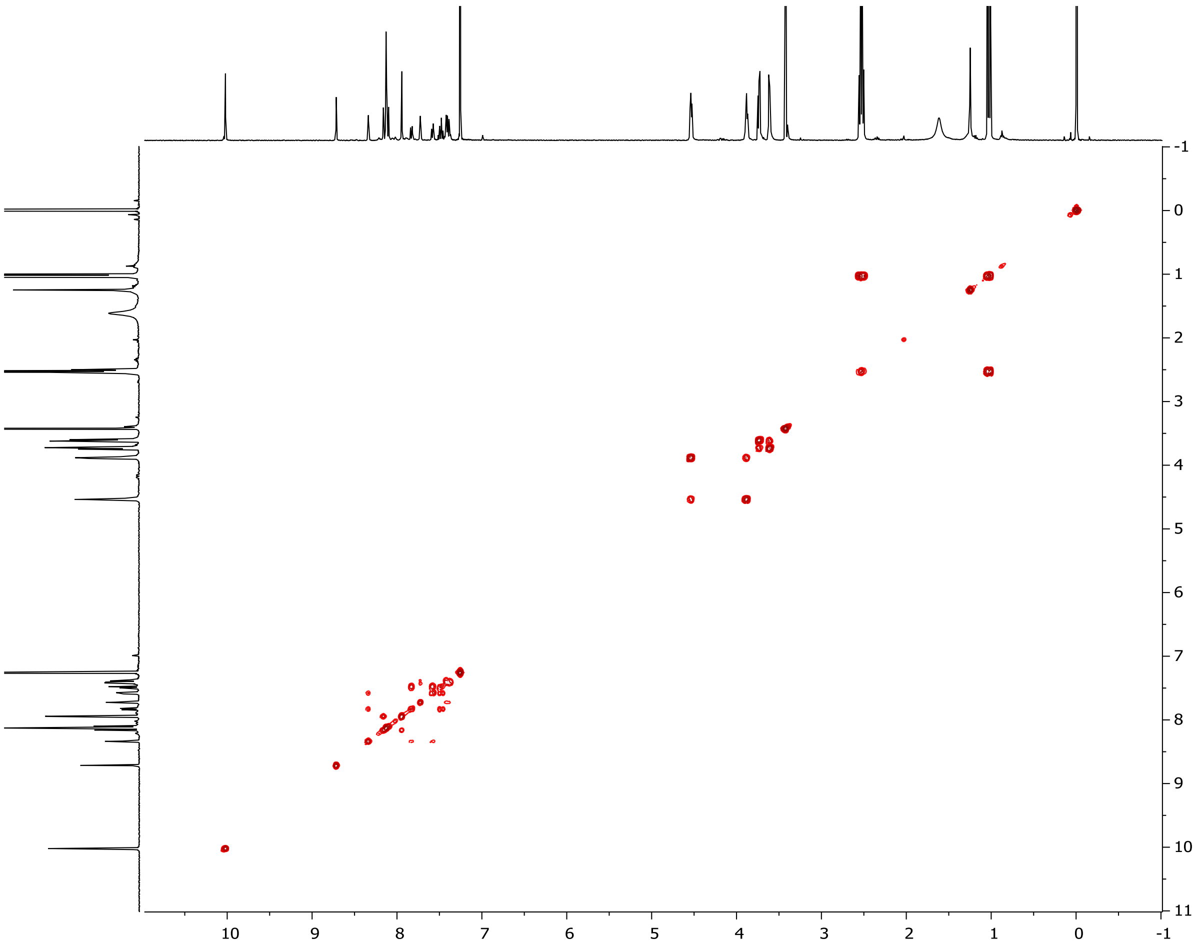

^{1}H NMR (400 MHz, $CDCl_3$ w/ 0.1% TEA)

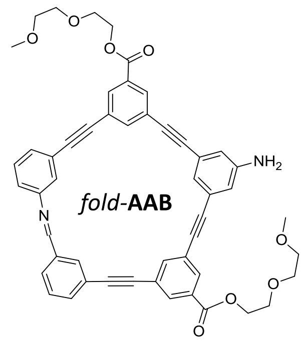

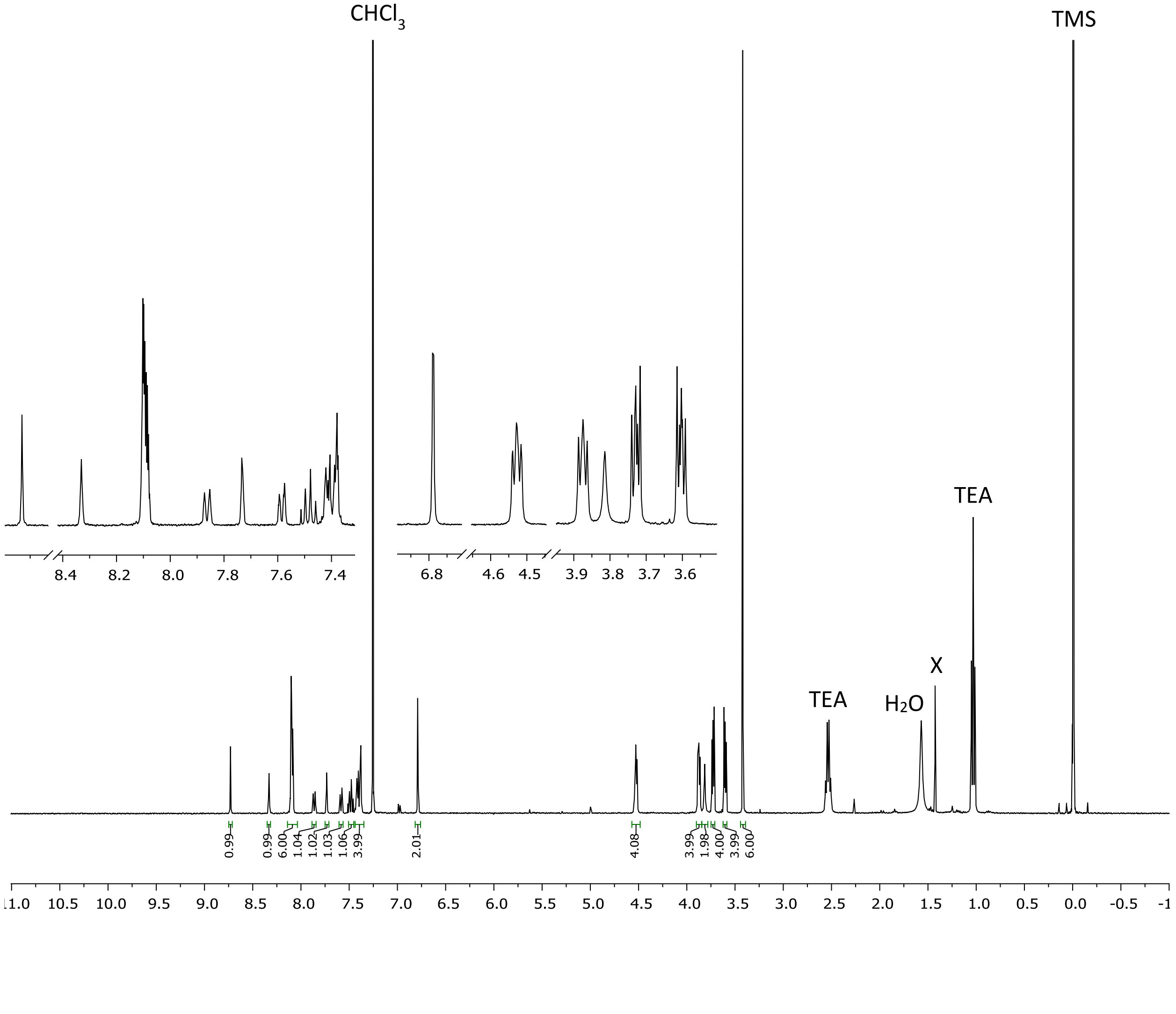

^{13}C NMR (100 MHz, $CDCl_3$ w/ 0.1% TEA)

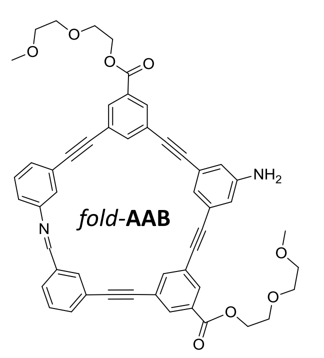

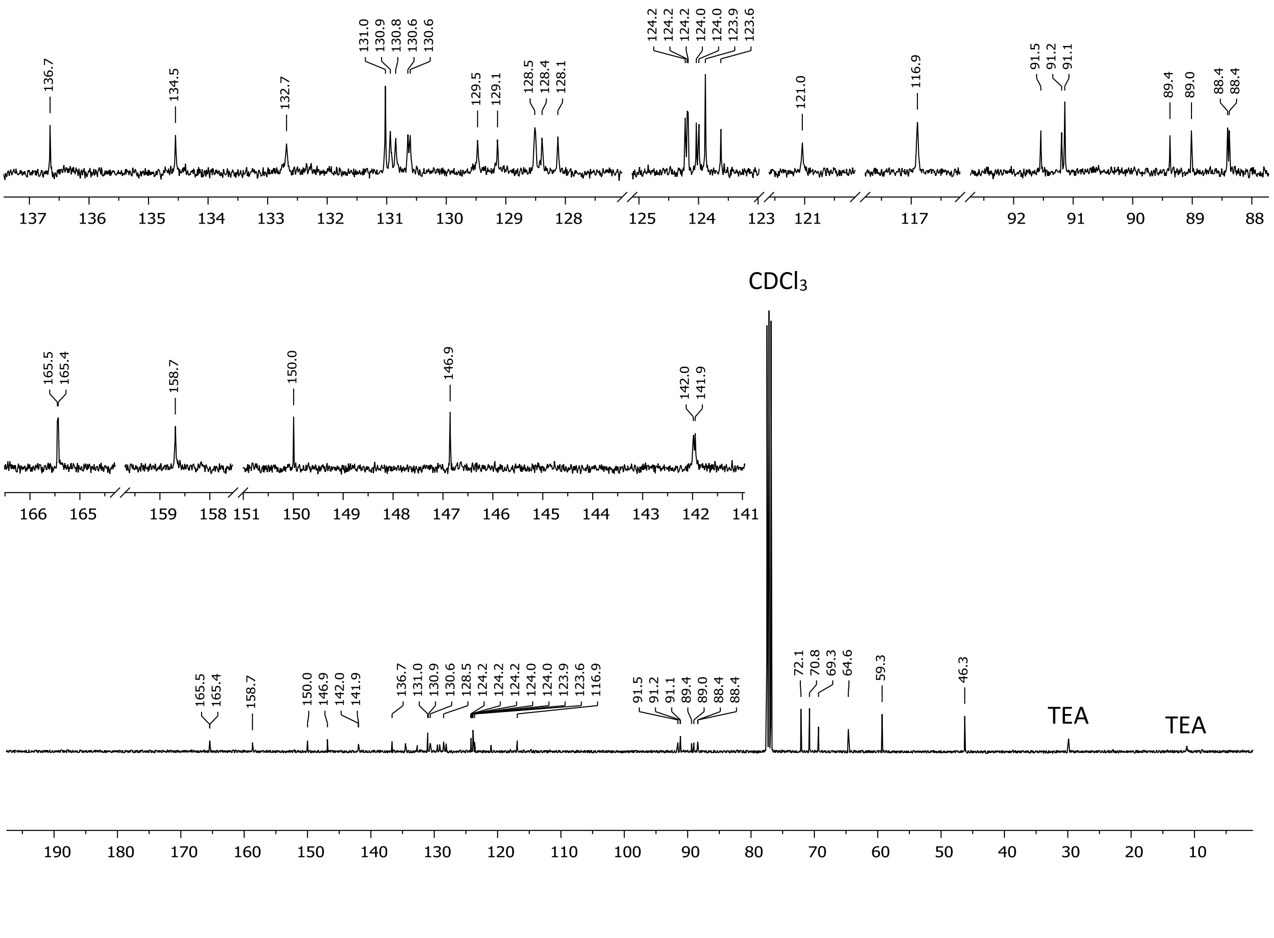

^{1}H-^{1}H ROSEY NMR (400 MHz, $CDCl_3$ w/ 0.1% TEA)

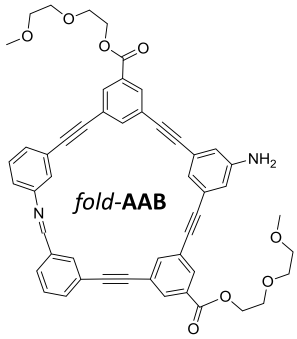

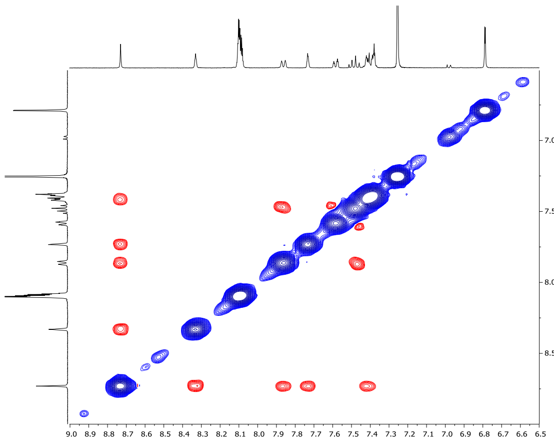

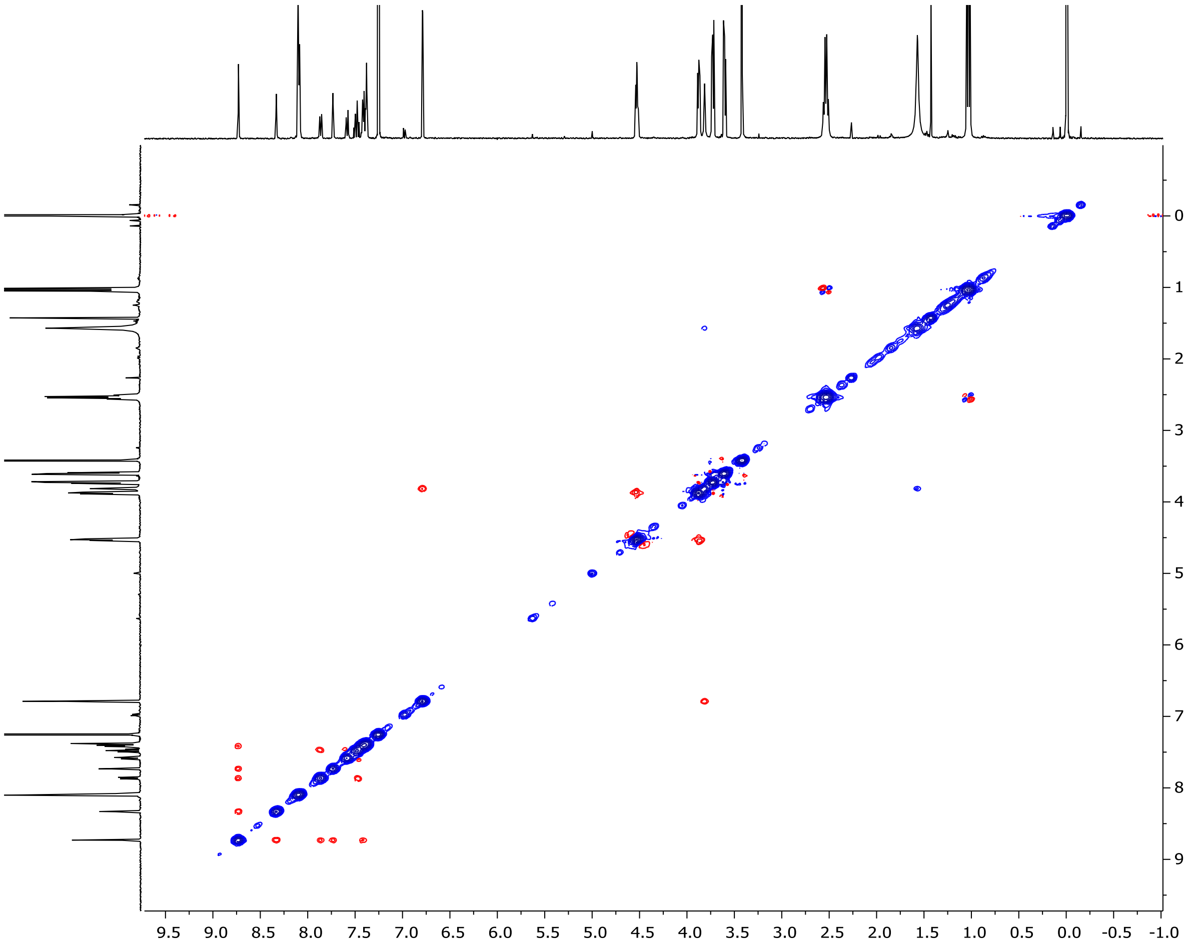

^{1}H-^{1}H COSY NMR (400 MHz, $CDCl_3$ w/ 0.1% TEA)

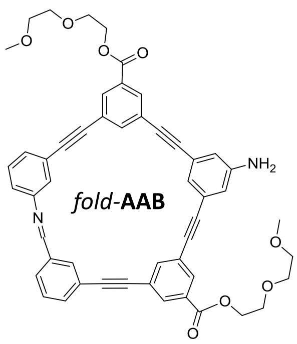

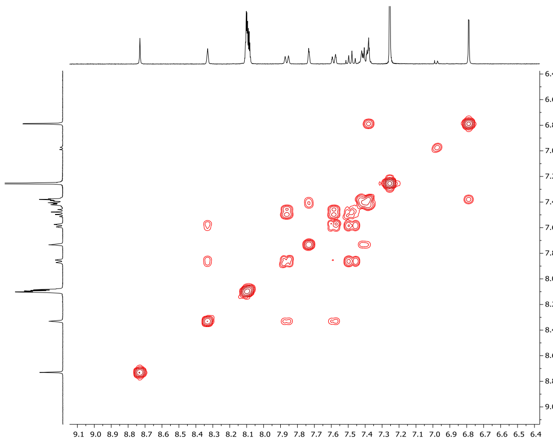

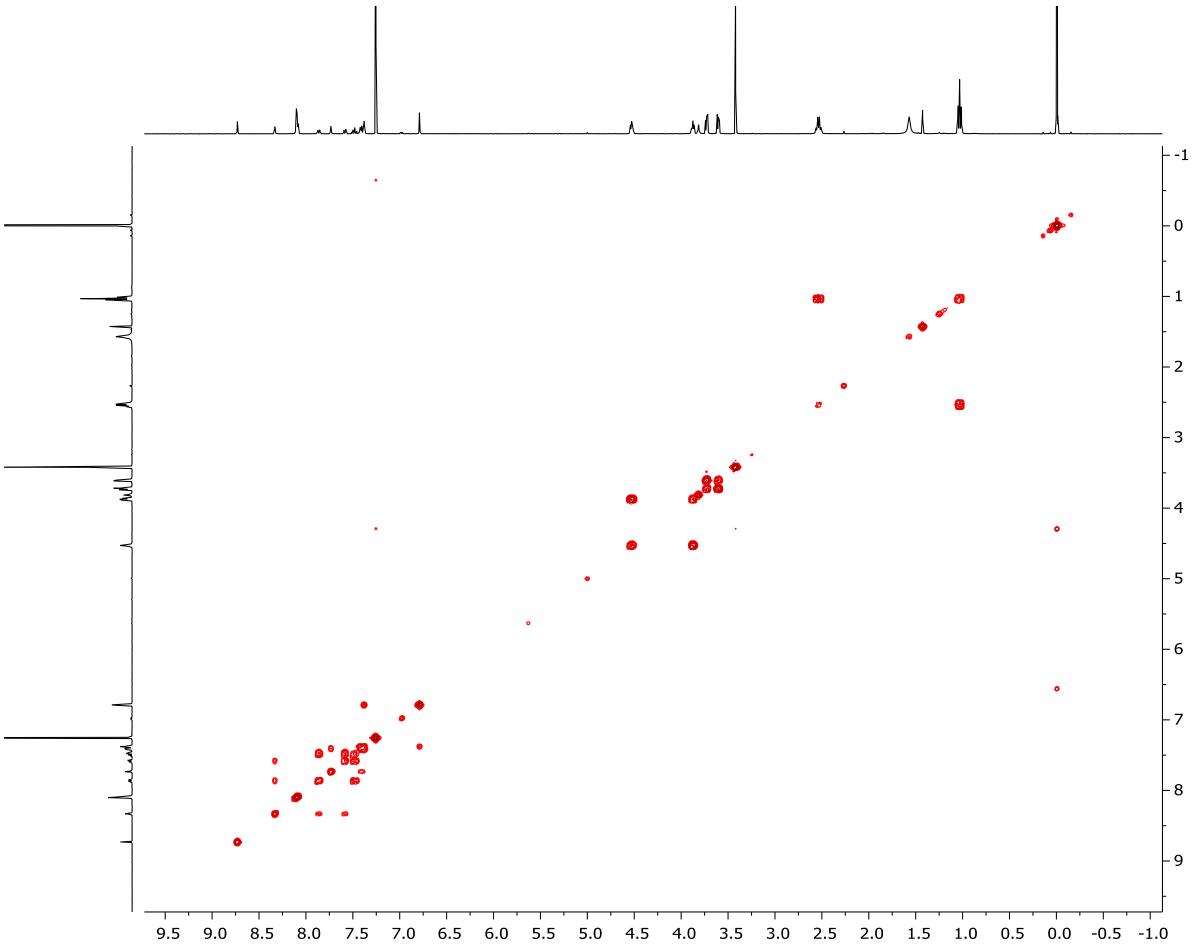

^{1}H NMR (400 MHz, $CDCl_3$ w/ 0.1% TEA, 50 °C)

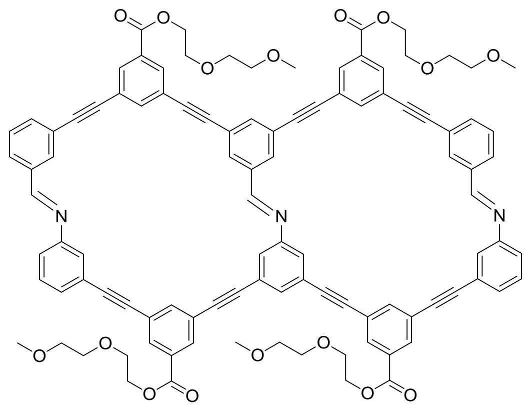

ds-**AAA· BBB**

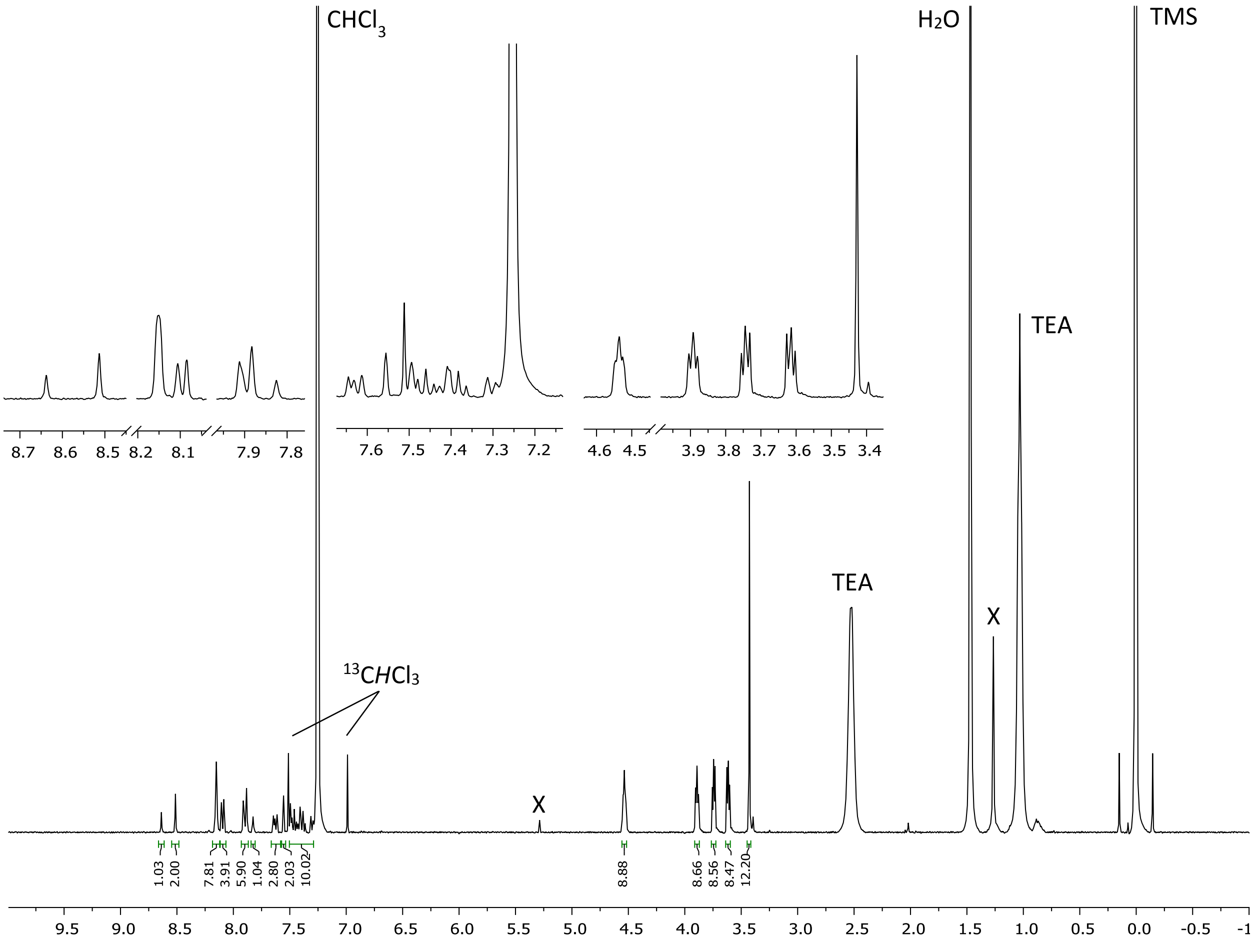

^{1}H NMR (400 MHz, $CDCl_3$ w/ 0.1% TEA, 50 °C)

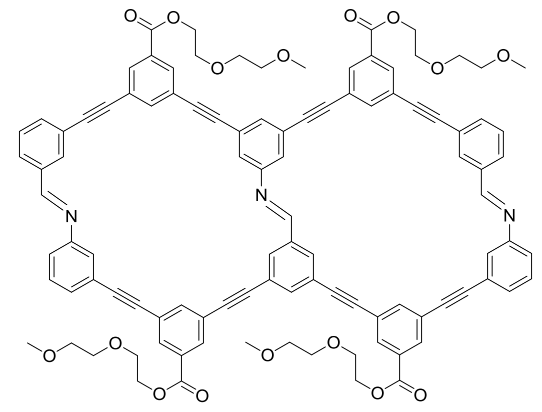

ds-**ABA· BAB**

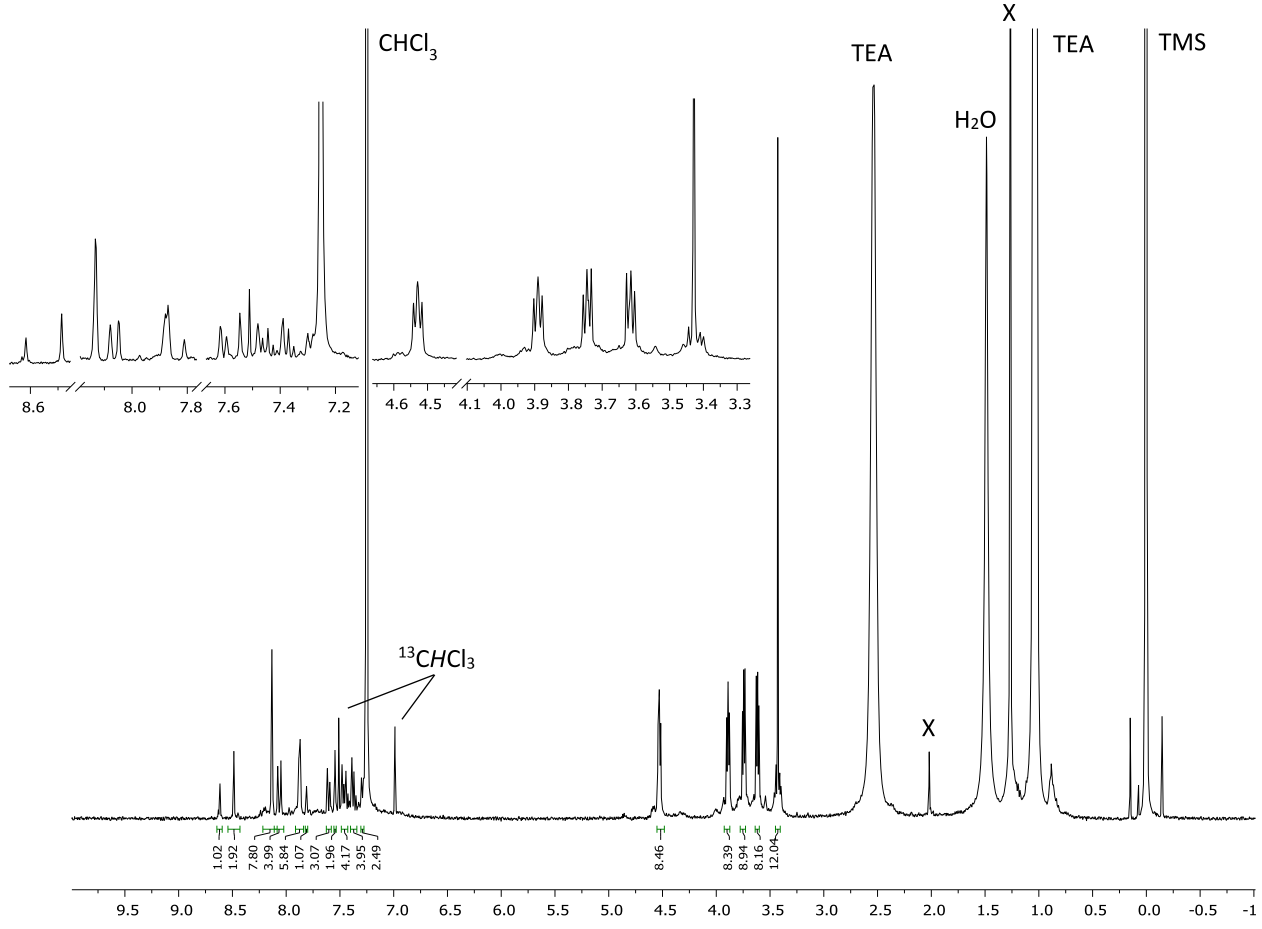

^{1}H NMR (400 MHz, $CDCl_3$ w/ 0.01% TEA, 50 °C)

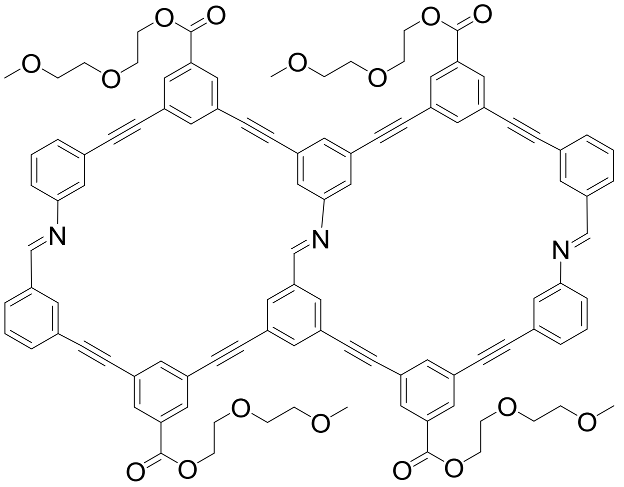

ds-**AAB· ABB**

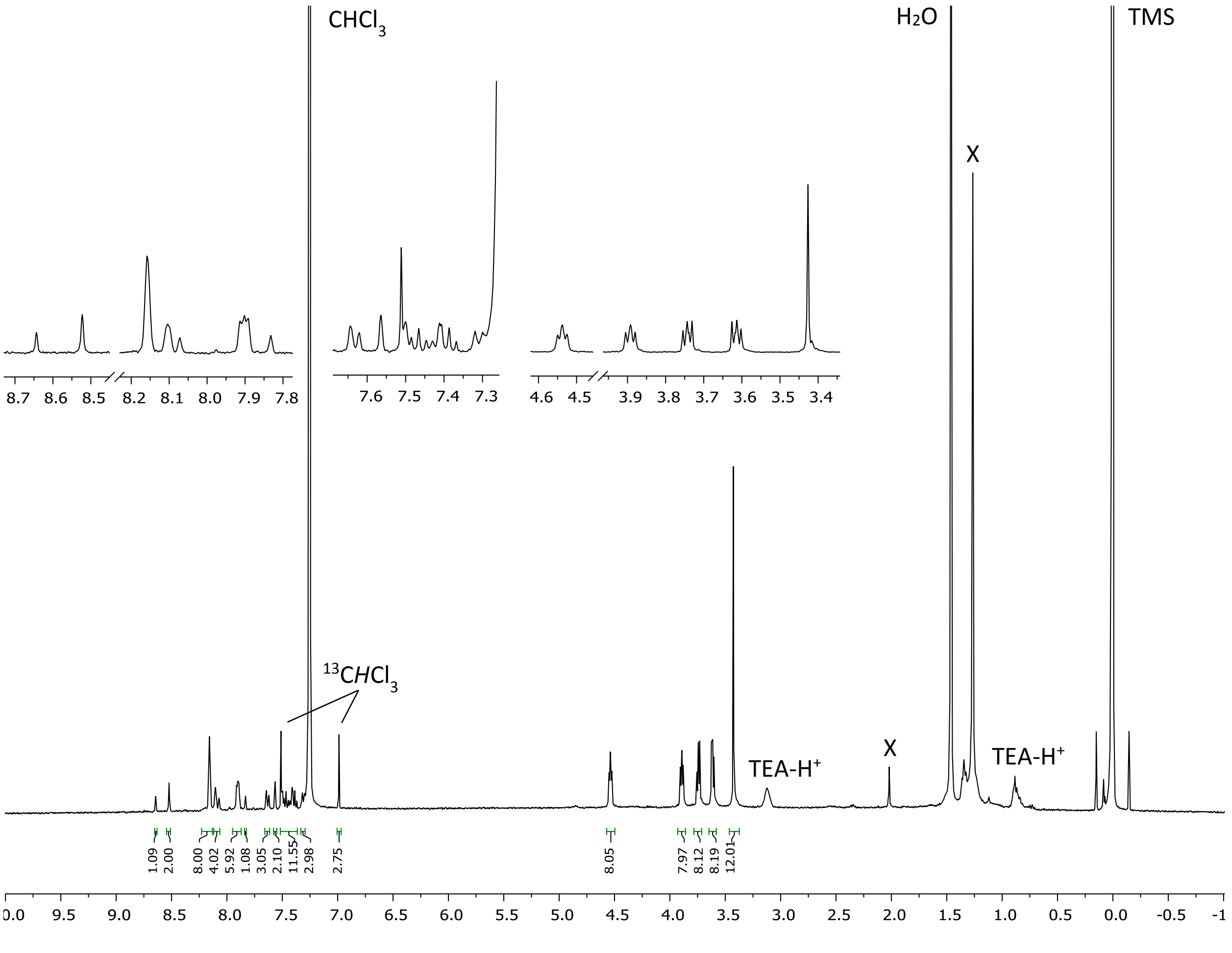

Supplement: Supplementary file 1 — ja2c06268_si_001.pdf [file ja2c06268_si_001.pdf]
